# The evolution of Runx genes II. The C-terminal Groucho recruitment motif is present in both eumetazoans and homoscleromorphs but absent in a haplosclerid demosponge

**DOI:** 10.1186/1756-0500-2-59

**Published:** 2009-04-17

**Authors:** Anthony J Robertson, Claire Larroux, Bernard M Degnan, James A Coffman

**Affiliations:** 1 Mount Desert Island Biological Laboratory, Salisbury Cove, Maine 04672, USA; 2 School of Biological Sciences, University of Queensland, St Lucia, 4072 QLD, Australia

## Abstract

**Background:**

The Runt DNA binding domain (Runx) defines a metazoan family of sequence-specific transcription factors with essential roles in animal ontogeny and stem cell based development. Depending on *cis*-regulatory context, Runx proteins mediate either transcriptional activation or repression. In many contexts Runx-mediated repression is carried out by Groucho/TLE, recruited to the transcriptional complex *via *a C-terminal WRPY sequence motif that is found encoded in all heretofore known Runx genes.

**Findings:**

Full-length Runx genes were identified in the recently sequenced genomes of phylogenetically diverse metazoans, including placozoans and sponges, the most basally branching members of that clade. No sequences with significant similarity to the Runt domain were found in the genome of the choanoflagellate *Monosiga brevicollis*, confirming that Runx is a metazoan apomorphy. A contig assembled from genomic sequences of the haplosclerid demosponge *Amphimedon queenslandica *was used to construct a model of the single Runx gene from that species, *AmqRunx*, the veracity of which was confirmed by expressed sequences. The encoded sequence of the Runx protein OscRunx from the homoscleromorph sponge *Oscarella carmella *was also obtained from assembled ESTs. Remarkably, a syntenic linkage between *Runx *and *Supt3h*, previously reported in vertebrates, is conserved in *A. queenslandica*. Whereas *OscRunx *encodes a C-terminal Groucho-recruitment motif, *AmqRunx *does not, although a *Groucho *homologue is found in the *A. queenslandica *genome.

**Conclusion:**

Our results are consistent with the hypothesis that sponges are paraphyletic, and suggest that Runx-WRPY mediated recruitment of Groucho to *cis*-regulatory sequences originated in the ancestors of eumetazoans following their divergence from demosponges.

## Findings

The Runt domain (Runx) is a highly conserved 128 amino acid sequence motif that defines a metazoan family of sequence-specific DNA binding proteins required for the ontogeny of each of the animal species in which it has been functionally studied, as well as for the regulation of somatic stem cells and development of the lineages to which they give rise [1-4]. Runx genes facilitate developmental coordination of cell proliferation and differentiation [1], integrating the transduction of multiple signalling pathways [2] by nucleating the assembly of signal-responsive *cis*-regulatory modules [5]. Runx genes have only been found in animals [6,7], suggesting that they may have evolved in concert with metazoan systems for developmental signalling.

All heretofore known Runx genes encode proteins that bear at their C-terminus a WRPY sequence motif (or a close variant thereof), which functions to recruit the Groucho/TLE corepressor to the *cis*-regulatory system [8-12]. Runx-WRPY mediated recruitment of Groucho is relatively weak and controlled by *cis*-regulatory sequence context [12,13]. Depending on such context, Runx proteins can also function as Groucho-independent repressors, as well as activators [8,14].

The purpose of this study was to extend our previous investigation of the evolution of Runx genes [6] by analyzing and comparing several new Runx gene sequences collected from recently sequenced genomes of lophotrochozoans and basally branching metazoans (see Additional File 1 for detailed methods). Although cnidarian and sponge Runx genes were described in a recent report [7], that study left open the question of whether the sponge Runx proteins bear a C-terminal Groucho recruitment motif. To address that question we examined Runx-encoding genomic and cDNA sequences from two sponges (*Amphimedon queenslandica *and *Oscarella carmela*), and compared these to Runx sequences collected from a phylogenetically broad sampling of other metazoan genomes, including that of the placozoan *Trichoplax adhaerens *[15].

### Runx is a metazoan synapomorphy that has undergone independent duplications in a subset of triploblast lineages

**Figure 1:**
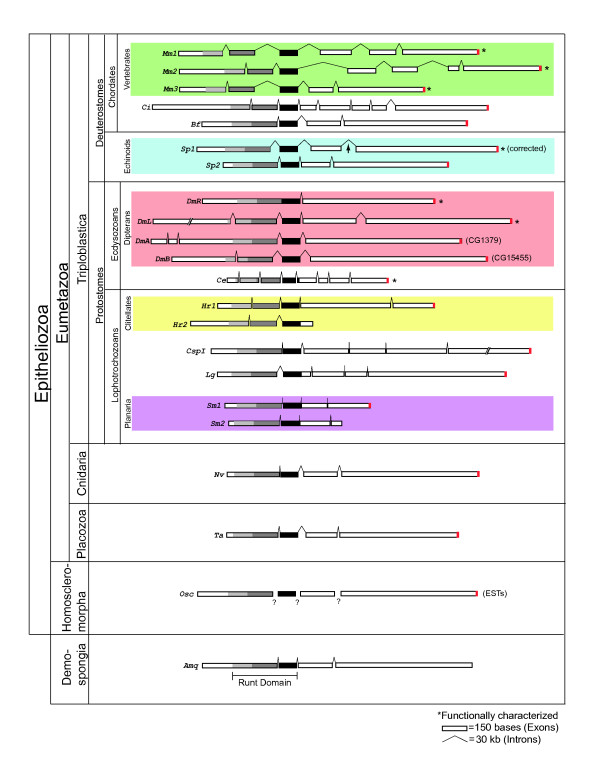
**Schematic structure of Runx genes from the major metazoan clades**. Scale models of Runx genes described previously [6,7] from mouse (*Mus musculus*, *Mm*), sea squirt (*Ciona intestinalis*, *Ci*), fruit fly (*Drosophila melanogaster*, *Dm*), nematode worm (*Caenorhabditis elegans*, *Ce*), and sea anemone (*Nematostella vectensis*, *Nv*) are shown in comparison to new models obtained from various recent genome projects (Table 1). The latter include Runx genes from lancelet (*Branchiostoma floridae*, *Bf*), sea urchin (*Strongylocentrotus purpuratus*, *Sp*, corrected; the arrow points to an intron that was previously missed [6,21]), leech (*Helobdella robusta*, *Hr*), polychaete (*Capitella sp. I*, *CspI*), snail (*Lottia gigantea*) planarian (*Schmidtea mediterranea*, *Sm*), placozoan (*Trichoplax adherens*, *Ta*), and demosponge (*Amphimedon queesnslandica*, *Amq*). The Runt domain is shaded grey and black, with the black box denoting the highly conserved exon encoding its C-terminal end. The C-terminal WRPY Groucho-recruitment motif is shaded Red. A hypothetical model of the homoscleromorph sponge Runx gene (*Oscarella carmela*, *Osc*) is shown; although as yet there is no genomic sequence from which exon-intron structure of this gene can be inferred (as indicated by question marks), the predicted exonic coding sequences containing the Runt domain and C-terminal LWRPY are represented in assembled ESTs.

Figure 1 depicts several representative examples of previously known [6,7] or newly revealed (Table 1) Runx genes from across metazoan phylogeny, clustered according to the phylogenetic topology obtained by Sperling et al. [16]. As recently shown by Sullivan et al. [7], Runx-encoding sequences extend to the base of the metazoan family tree, with single orthologues encoded in the genome of the haplosclerid demosponge *A. queenslandica *and in expressed sequence tags from the homoscleromorph sponge *O. carmela*. Similarly, the anthozoan cnidarian *Nematostella vectensis *and the placozoan *Trichoplax adherens *each have a single Runx gene, as do several triploblast species, including the lancelet *Branchiostoma floridae *and the sea squirt *Ciona intestinalis *among deuterostomes; the nematode *Caenorhabditis elegans *among ecdysozoans; and the polychaete *Capitella sp.I *and the mollusk *Lottia gigantea *among lophotrochozoans. In contrast, vertebrates, sea urchins (*Strongylocentrotus purpuratus*), dipteran insects (*Drosophila melanogaster*), clitellate annelids (*Helobdella robusta*), and planarians (*Schmidtea mediterranea*) each have two or more Runx genes.

**Table 1:** Sources of sequences used in this analysis

*Species*	*Genome Database (URL)*	*Version*	*NCBI Acc. No*.
*S. purpuratus*		2.1	NW_001330224
*B. floridae*		1.0	N.A.
*H. robusta*		1.0	N.A.
*Capitella sp*. I*		1.0	N.A.
*L. gigantean*		1.0	N.A.
*T. adhaerens*		1.0	N.A.
*S. mediterranea*		1.3.14	N.A.
*A. queenslandica*		N.A.	N.A.
O. carmela**		N.A.	N.A.

The table identifies the genome project and assembly version from which each of the new sequences described here was obtained, as well as available NCBI genomic contig reference assemblies. The sequences and links to each locus on the respective genome browsers are provided in Additional File 2. N.A., not available. *Complete gene model obtained from raw contig sequence using GeneScan. **ESTs only.

Comparison of the gene architectures suggests that the primordial *Runx *gene contained three introns, the first of which interrupts the coding sequence of the Runt domain (found in every representative except for the insect *runt *orthologues), the second of which lies at the C-terminal end of the Runt domain (found in all of the representatives except two, *HrRunx2 *and *LgRunx*, both from lophotrochozoans), and the third lying between the two exons that encode the poorly conserved C-terminal sequence of the protein (missing in three of the insect genes and one of the leech genes; Fig. 1). This basic four-exon architecture is displayed by the demosponge, placozoan and anthozoan Runx genes, and among the known triploblast Runx genes, by the two sea urchin paralogues, the single lancelet orthologue, and the two planarian paralogues. Except for the additional intron within the sequence that encodes the N-terminal half of the Runt domain in all the vertebrate paralogues (Fig. 1), the basal architecture is conserved in vertebrate *Runx3*, which supports previous propositions for that gene being the most ancient of the vertebrate paralogues [17]. The additional N-terminal intron in *Runx3*, which is also found in each of the other vertebrate Runx paralogues, is also found in the *C. intestinalis *orthologue (but not in the cephalochordate *B. floridae*), consistent with recent phylogenies that place cephalochordates basal to {urochordates+vertebrates} in the chordate lineage [18].

**Figure 2:**
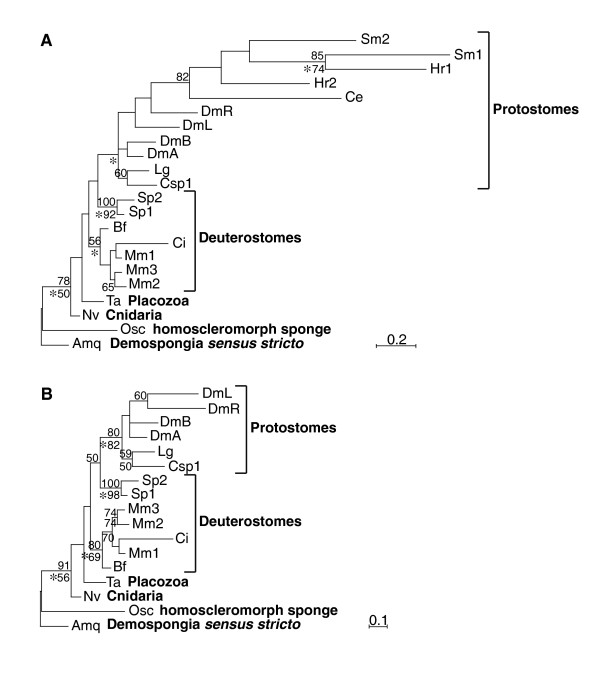
**Bayesian trees of Runx sequences**. In a first analysis (A), all the genes from Figure 1 were included and, in a second analysis (B), long-branched taxa (Runx genes from *S. mediterranea*, *H. robusta*, and *C. elegans*) were excluded from the dataset. The trees were calculated using a multiple sequence alignment of amino acid sequences corresponding to the Runt domain of each species. Percentages of bootstrap support greater than or equal to 50% are indicated above the node for the distance analysis (Phylip 3.6; 1000 replicates) and below the node for the maximum likelihood analysis (Phylip 3.6; 100 replicates). An asterisk under the node indicates a Bayesian posterior probability greater than or equal to 95%. Abbreviations as in Figure 1.

To confirm and extend previous analyses of Runx family relations [6,7], we used our expanded Runx sequence dataset to calculate trees by Bayesian, distance neighbor-joining (NJ), and maximum likelihood (ML) methods. The three trees have slightly different topologies; the Bayesian tree is shown in Figure 2A. All three analyses confidently support the branch separating the two sponge Runx genes from eumetazoan genes. Additionally, the protostome and chordate clades are recovered in all three trees but the positions of cnidarian, placozoan, and echinoderm genes differ between analyses. While only the NJ tree places echinoderms correctly inside a deuterostome clade, this clade also erroneously includes cnidarian and placozoan genes. Bayesian and ML analyses correctly place the latter two genes at the base of the bilaterian clade but wrongly group echinoderm genes with protostome genes. Relationships within the protostomes are unclear and none of the three analyses separates these genes into lophotrochozoan and ecdysozoan clades. This may be due to long-branch attraction between the Runx genes from *S. mediterranea*, *H. robusta*, and *C. elegans*. Thus, these genes were removed in a second set of analyses (Fig. 2B), where a lophotrochozoan clade and a clade comprising the four *D. melanogaster *genes are recovered in all three trees. These analyses suggest that there was only one Runx gene in the lineage between the metazoan and the lophotrochozoan-ecdysozoan last common ancestors. Hence, the multiple Runx genes present in some of the animals in this study are most likely the products of independent duplications within each of the lineages [6] (Fig. 1, colored boxes; note that a second sea urchin Runx gene, *SpRunt-2*, was recently found to be encoded in the sea urchin genome [19,20], in contradiction to several previous reports [1,6,7,21]).

Previous reports have noted the absence of any Runx homologues in sequenced genomes of unicellular organisms [6,7], including the choanoflagellate *M. brevicolis *[22], a member of the Holozoa taxon that is most closely related to Metazoa. We confirmed the absence of a Runx sequence motif in the *M. brevicolis *genome using tBLASTn searches. Thus, the Runt domain appears to have evolved in concert with complex multicellularity in the animal clade. Furthermore, unlike many other metazoan-specific transcription factor classes [23], the Runx gene did not duplicate in early animals, or even within some of the bilaterian lineages.

### *AmqRunx *lacks a Groucho recruitment motif

**Figure 3:**
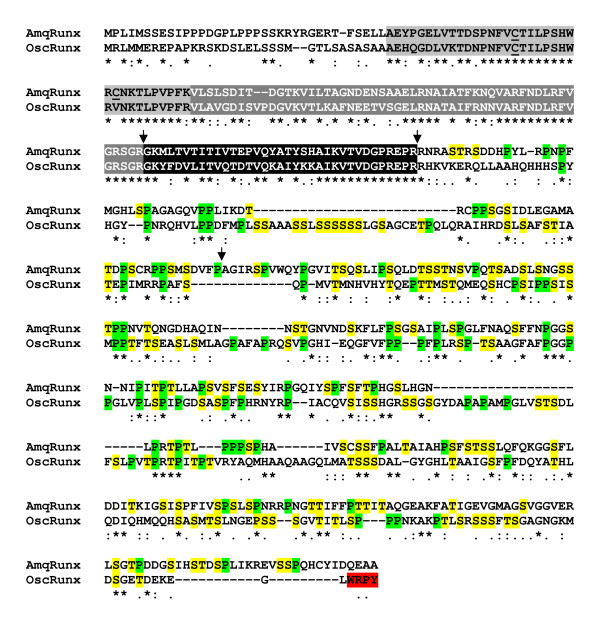
**Alignment of AmqRunx and OscRunx amino acid sequences**. Identities are marked with asterisks, whereas conservative changes are marked with dots. The Runt domains are highlighted in grey and black, for reference to the scheme depicted in Figure 1. The arrows indicate predicted intron positions with respect to the coding sequence of *AmqRunx*. Proline residues in the C-terminal domains are highlighted in green, whereas serines and threonines are highlighted in yellow. The C-terminal WRPY motif in OscRunx is highlighted in red.

As reported previously [7], Runx genes are found in both the haplosclerid demosponge *A. queenslandica *and the homoscleromorph sponge *O. carmela*. Although genome sequence is not yet available for the latter, a sequence encoding a Runx protein was recovered from an assembly of available ESTs. The predicted OscRunx protein terminates with the amino acid sequence WRPY (Fig. 3) [see Additional File 2], the C-terminal Groucho-recruitment motif found encoded in all heretofore known Runx genes (Fig. 1). Note that there are vertebrate splice variants that lack a C-terminal WRPY [24-26], and that one each of the two leech and two planarian paralogues do not appear to terminate in WRPY (Fig. 1) [see Additional File 2]. Thus, some contexts have functional requirements for Runx protein isoforms lacking a C-terminal WRPY. Nevertheless, all of the eumetazoan species depicted in Fig. 1 (as well as the homoscleromorph sponge) encode at least one Runx protein that terminates in WRPY or a close variant thereof.

A genomic sequence contig from *A. queenslandica *was predicted to encode a Runx gene with four exons, displaying an architecture very similar to that of the placozoan and cnidarian genes (Fig. 1) [7]. The predicted coding sequence of *AmqRunx *is 1,566 bp with the Runt domain contained within the first 474 bp. As is typical for Runx proteins, the predicted C-terminal domain of AmqRunx (amino acid residues 159–479) is enriched for proline (12%), serine (16%), and threonine (7%) residues, a PST enrichment similar to that previously reported for the C-terminal domain of NvRunx [7] and that displayed by the C-terminal domain of OscRunx (Fig. 3). Surprisingly however, the C-terminus of AmqRunx does not bear the WRPY motif or any variant thereof (Fig. 3). Furthermore, no open reading frames encoding WRPY were found along the genomic contig in which *AmqRunx *is found. The *A. queenslandica *genome does however encode a *bona fide *Groucho homologue (Additional File 3 and unpublished data), as well as several transcription factors that are predicted to interact with Groucho [12], including a hairy/Hey homologue with a FRPW motif and a number of NK class genes with an engrailed homology 1 (EH-1) motif ([27,28]; BMD, unpublished data).

The lack of a C-terminal WRPY motif in *AmqRunx *was verified by expressed sequence data. Based on alignment with genomic DNA, EST sequence 2941805_1 was found to encode the last 115 bp of the *AmqRunx *coding sequence, the stop codon, and an additional 626 bp of 3' UTR spanning two exons. In order to confirm that this EST was transcribed from *AmqRunx*, oligonucleotide primers – forward primer in the Runt domain and reverse primer in the EST-encoded 3' UTR region – were used to amplify the sequence both from *A. queenslandica *adult and embryonic RNA. An amplicon of the correct size and sequence was obtained (Additional File 4), thus confirming the veracity of the *AmqRunx *gene prediction.

**Figure 4:**
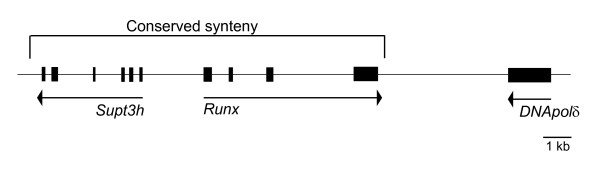
**Schematic of the 20 kb genomic sequence contig bearing *AmqRunx***. Predicted exons are shown as black boxes. The syntenic relationship between *Runx *and *Supt3h *is conserved between demosponge (*A. queenslandica*) and mouse (*Mus musculus*), and is also found (at least) in the genomes of a cnidarian (*N. vectensis*), a basal chordate (*B. floridae*), and a teleost (*Takifugu rubripes*; [29]).

The contig bearing *AmqRunx *contains sequences predictive of additional genes flanking the Runx gene (Fig. 4), which argues against the possibility that the *AmqRunx *gene model is missing a C-terminal exon that might produce alternative splice variants. Moreover, the veracity of the contig assembly is further supported by the remarkable fact that a syntenic relationship between *Runx *and *Supt3h*, previously reported to exist in vertebrates [29] and which we found also to exist in cnidarians (*N. vectensis*), lancelets (*B. floridae*), and polychaetes (*Capitella sp. I*), is conserved in the demosponge (Fig. 4).

**Figure 5:**
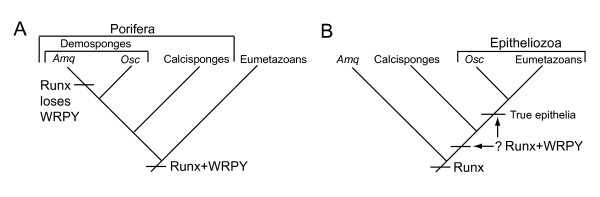
**Scenarios for Runx-WRPY evolution mapped onto alternative metazoan phylogenies**. (A) Conventional phylogeny wherein the demosponge *Amphimedon queenslandica *(*Amq*) and the homoscleromorph sponge *Oscarella carmela *(*Osc*) are both classified as demosponges within the phylum Porifera. This scenario suggests that the WRPY motif was lost in the demosponge sub-lineage leading to *Amq*. (B) Alternative phylogeny wherein sponges are paraphyletic. In this tree homoscleromorphs are a sister group of eumetazoans within Epitheliozoa [16], which would imply that Runx gained the WRPY motif in the ancestors of the latter group following their divergence from demosponges, either prior to or after divergence from calcisponges.

Although homoscleromorph sponges are still commonly grouped with demosponges in the phylum Porifera (Fig. 5A), this classification has been called into question, as has the monophyly of sponges (and hence 'Porifera' as a true phylum) [16]. The fact that *AmqRunx *lacks a C-terminal WRPY motif is consistent with the more recent proposition that sponges are paraphyletic [16,30], with calcisponges and homoscleromorphs branching after demosponges along the lineage leading to eumetazoans (Fig. 5B). The conventional scenario, which holds that sponges are monophyletic (Fig. 5A), would require that several characters held in common between eumetazoans and homoscleromorph sponges (i.e., acrosomes, true epithelia, and a C-terminal WRPY motif linked to Runx) be either convergent homoplasies, or metazoan pleisiomorphies that were all lost in the demosponge lineage leading to *A. queenslandica*. Although it is possible that the loss of multiple characters occurred within the demosponge lineage, it is unlikely that body plan simplification is in itself sufficient to relax the selection pressure for maintaining the Runx-WRPY linkage, as evidenced by its maintenance in placozoans. The more parsimonious scenario is that the C-terminal WRPY motif of Runx proteins, and presumably the consequent recruitment of Groucho to a subset of Runx target *cis*-regulatory modules, originated in eumetazoan ancestors following their divergence from the sponge lineage leading to *A. queenslandica *(Fig. 5B). An interesting possibility is that the Runx associated WRPY motif originated in Epitheliozoa {eumetazoans and homoscleromorphs} [16], which would suggest that Runx-WRPY mediated *cis*-regulatory recruitment of Groucho is functionally linked to the evolution and development of an epithelium. Testing this possibility awaits the sequencing of a calcisponge Runx gene.

## Competing interests

The authors declare that they have no competing interests.

## Authors' contributions

AJR performed BLAST searches, sequence assemblies, alignments, and computational construction of gene models. CL independently verified the *A. queenslandica *contig assembly and Runx gene model, performed the phylogenetic analyses, and obtained the PCR amplicon of *AmqRunx *cDNA. BMD performed some sequence assemblies, provided intellectual guidance and assisted in the writing of the manuscript. JAC performed some of the BLAST searches and sequence alignments, and drafted the manuscript and figures. All authors read and approved the final manuscript.

## Supplementary Material

Additional File 1 **Bioinformatics and Cloning Details**. This file provides a detailed description of the methods used to obtain the Runx gene sequences and phylogenetic trees presented in this paper. Click here for file

Additional File 2 **Sequences of Runx genes listed in Table 1**. This file provides gene, CDS, mRNA, and/or predicted peptide sequences of each of the Runx genes that are described for the first time (or corrected, in the case of *SpRunt-1*) in this report. For the two sea urchin genes, URLs are given to the scaffold coordinates on the SpBase genome browser, as well as to the original genome annotations. For gene sequences obtained from JGI genome projects, links are provided to the scaffold coordinates on the JGI genome browser. Click here for file

Additional File 3 ***AmqGroucho *sequence**. This file provides an *A. queenslandica *genomic trace sequence that encodes peptides homologous to Groucho, identified by tBLASTn using the TLE-domain (pfam03920: TLE_N), and confirmed by reciprocal BLASTx. Click here for file

Additional File 4 ***AmqRunx *cDNA sequence**. This file provides the cDNA sequence of *AmqRunx *containing the N-terminus, Runt domain, predicted C-terminus, and some 3' UTR, obtained by RT-PCR from adult and embryonic RNA. Click here for file
