# Supplementary material for: The evolution of Runx genes II. The C-terminal Groucho recruitment motif is present in both eumetazoans and homoscleromorphs but absent in a haplosclerid demosponge
Source: BMC Res Notes. 2009 Apr 17;2:59. doi: 10.1186/1756-0500-2-59 (PMC2674455; doi:10.1186/1756-0500-2-59)
Supplement: Additional File 1 — Bioinformatics and Cloning Details. This file provides a detailed description of the methods used to obtain the Runx gene sequences and phylogenetic trees presented in this paper. [file 1756-0500-2-59-S1.doc]

**Additional File 1: Bioinformatics and Cloning Details**

## Collection and assembly of Runx genes

Runx genes were identified in several recently sequenced genomes (Table 1, Additional File 2) by tBLASTn searches using the Runt domain from sea urchin SpRunt-1. The assembled genomes of *N. vectensis*, *B. floridae, H. robusta, Capitella sp*. *I*, *L. gigantea*, and *T. adhaerens* were interrogated using the BLAST server of the US Department of Energy Joint Genome Institute ([http://genome.jgi-psf.org](http://genome.jgi-psf.org/)). The computationally generated Runx gene models posted on the JGI browser for *Capitella sp.I* and *T. adhaerens* were incomplete, and additional exons were identified in the raw sequence using the Genescan gene prediction program (<http://genes.mit.edu/GENSCAN.html>), The planaria genome was interrogated using the BLAST server of the *S. mediterranea* genome database (<http://smedgd.neuro.utah.edu/index.html>). WGS Traces from the genome of *A. queenslandica* and ESTs from both *A. queenslandica* and *O. carmela* were searched *via* the BLAST server of the Compagen Comparative Genomics Platform (<http://compagen.zoologie.uni-kiel.de/index.html>), and the sequences so identified were assembled using the Contig Express program in Vector NTI. Exonic sequences within the assembled genomic contig containing *AmqRunx* were identified using Genescan. The *Supt3h* and *DNApolδ* genes on this contig were identified by BLASTx searches at NCBI.

## Phylogenetic analyses

Bayesian, maximum likelihood, and distance neighbor-joining phylogenetic analyses of aligned Runt domains from various metazoan Runx genes were carried out as previously described (Larroux et al., *Mol Biol Evol* 2008, 25:980-996).

## Cloning of *AqRunt* cDNA

Primers were designed to amplify the *AmqRunt* gene region from positions 27 to 1704, spanning from the Runt domain (forward primer: TGATGGTACCAAGGTGATACT) to the EST-encoded 3' UTR region (reverse primer: TGGTAGGATGGAGAGTGATTG). RT-PCR was conducted on adult and embryonic RNA as previously described (Larroux et al., *Evol Dev* 2006, 8:150-173).
