# Supplementary material for: The evolution of Runx genes II. The C-terminal Groucho recruitment motif is present in both eumetazoans and homoscleromorphs but absent in a haplosclerid demosponge
Source: BMC Res Notes. 2009 Apr 17;2:59. doi: 10.1186/1756-0500-2-59 (PMC2674455; doi:10.1186/1756-0500-2-59)
Supplement: Additional File 2 — Sequences of Runx genes listed in Table 1. This file provides gene, CDS, mRNA, and/or predicted peptide sequences of each of the Runx genes that are described for the first time (or corrected, in the case of SpRunt-1) in this report. For the two sea urchin genes, URLs are given to the scaffold coordinates on the SpBase genome browser, as well as to the original genome annotations. For gene sequences obtained from JGI genome projects, links are provided to the scaffold coordinates on the JGI genome browser. [file 1756-0500-2-59-S2.doc]

>SpRunt-1 mRNA (genome scaffold at http://sugp.caltech.edu/cgi-bin/gbrowse/chado2.1/?name=scaffold_v2_57995%3A80309-130187) (annotation at <http://annotation.hgsc.bcm.tmc.edu/Urchin/cgi-bin/pubInsertGene.cgi?gene_id=GLEAN3_06917> and http://annotation.hgsc.bcm.tmc.edu/Urchin/cgi-bin/pubInsertGene.cgi?gene_id=GLEAN3_25612)

CCACGCGTCCGGCCACTTGTCCACATGTATACTGTGGCTCGCTGTATATTCGCACCTACGATTACTATCACAATTTCGGCTCTCATGCTGCAACTACAAGCAGATTTACCGTTTGGGGGACGTTTAAAAACCCTCGCGATTATTTACTAGCGTACATTTGGCTTAGGTTTTTATTTGGAACGCTTATAAACTGGTGACCACAATTTGGTGAACCATTTGCCTTCAATGACACCGTGCGATTTGGTTCAAAATATTATATTTTTGTCCGCATAATAATACTCTCGAGCAGTCTTATCTAATACCACCGCTACTACATTGCTTGCTCCTGATAAACTTTTTTTTTCGGTGTTGCTACCAAGAACGTGTGTGGAACATTTACCAACGCTTTGCGGTTTTGAGGTGTGGTTTGAGTATCTATTCAACATCGCCTGGCCCAGTCAGGGTTTCCGAAAAGAAATCTGAAATCATTTTTTCCCGCCCTTTCATCTCGATACAAACCACTCGCGTGGTAGAAACCCCACTCCGCATCAATGCATATTACTGACGTTAACGTTGATCATCTTCTGTCATCCACTGCTCCCTTGGCGAACCACCCCAGCAAGGACCCGGTACGGAGGAACAACCTACATAACAGTTATAAGATGGCCGAAGGGGGCCAGAGGAATAAGGCCTCATCCGTCTTCAAAGGAGGAGAACGGTCTATTGTGGACGCTTTATCGGAGTATCCCGGGGAGTTGGTGAAGACAGAGAGCCCTAACTTTGCCTGCTCTGTGCTCCCGAATCACTGGAGGTGCAATAAGAGTTTGCCCGTGGCTTTCAAAGTTGTTTCGCTGGGAGAGACCAAAGATGGTACGATGGTGACGATCGCAGCAGGGAACGATGAAAACTACTGCGCTGAATTGAAAAACAACACAGCAGTTATGAAGAACCAAGTGGCTCGTTTTAATGACCTGCGTTTCGTTGGACGATCGGGTCGAGGCAAGAGTTTTACCCTGTCCATCTTCATCTACACCAACCCTCCCCAGATCGCTACCTACAACCGTGCAATAAAGGTCACCGTGGATGGGCCAAGAGAACCCAGACGACCCAAACCCAAGGACCAGGAATCTCGTCTGATGCCCCCTCCCATCATCAACACCGGCCACCCTCATCCCTTCGGTGAGATCAACCCCCATCATCCCAACCATCACATTGGAAGACAGCAGTCGTATCAGAACCAGGGGCGCATGCCCAGATCATACCCTCTCAGTCCTACTTCTGGATCCTATGATAATATTCAGCATCAAGGGCAGGCTTCGAAGCCGTGGTCTTACTACAATCCTTACCAATCATCAGTCGCCCAACTCTCGGACACATCAATCCTCTCAGCACAGATCAAGACTGAACCTACGGAGCTTGCGTTGCTAGGTCAGCAGAACTCTACCCTTCAGCAATATCCCAAGCCTGATTCCCTCTACCCCACCTCCATCACCCGGTCCAGTGAGGTCCAAGACCCCCGCTTCGTCTACCCATCGACTCCGGCCGCGGTCAGCTCGGTATCATTCACGCCTTCTTCCATGTCAGTGCTGTCATCCGGGGTCGAATCACCGCGCACCATCTTGCCAATGACCCCCAATCCGTTCCCTCTGTCATCTCAAGATATCTTCTCAAGCTCCTCCACCGCCACGCCCGTCACTCTAACGTCGCCCCCTTACCTGCCAAACTCACCCCCCTACCCTCTCTACCCGCACCTCTACATGTCGTCTCCGTCGTCGCAAACCTACTACGATAGCTCACATCTACCAATGCTGCCATCGTCAACGAGACCGGAGGACAAGCAAGAGATCAAACATGATAACAGACCTGGGGAAGGCATCCCTCATATCCCTCATCCTGAGATGTCTCTGACGGTGGCTCTTAACTATGGTAGCCATCCTCAAAGCCAGATTGAACTCAATACAGCCAGGACCATGGGACAGAGCATGGGTGGGATGCATAACAGTGGGGTAGCGCTCATGCAGCAACATCGAAACCACAGCCCTGTCACTAACATCTCACCCATGCAACAAAGCATGGGACAAATGAATATCAACACCACCGTCGGACATTTACCCACCGTGGACGACTCAAGAAAAGAGGACGTCTGGCGGCCATATTGAATCCTAGAAGATCGTTGAGAAACTTTTGAGACTGTCTGTGTGAAGGATATGGACTCTATTTAATAGATTTGAAATCAGCGTATTCTCTGCAACACTGCTATGTACCTAGGTATTGATTCAGACTTGATGCAAAGTGGATTGCAGTGATCTGCATAAATGATGTGAGGTTTAACTCCTCAGCTTCTAATCATGTTGACTGGAGAAACACCATCCTGGCTCCTTAAAGTTTTATAATATTCATGATTCATTATTTCAATGTCATTCCATTTCATGTAGTCTCATGCACGTGTTTGTGTGTGTGCGTGTGTGTGAGCTGTATCATATTGCTTCACATTATCATCATCATCATCATCATTCTTGAATGAACAGAGGATTTATGGAGTGTACATAGTGAATTTTATGTACCATATAGTTCAAATGTGTGAGACGAAAGGCATGAATGATATTGCAAGATTCATGTACAGTAACAATTATTTGCAATGCAAGATTCTACACCCCATCTAAAGAAATATAATTCATGTTTTGGATGATTGTATTGTCTATTAAGAGTCTGAAAGGTATGAATTCATACACTCTAACACAGAGTTAACGCCCTCCCGGCTCTCAGCAGGCGATCACATGATAGACGAGCCTGTCGCGAAACAAGAACAAATTAATACTCATTCATTGTAAATGTCGTAGTAGATATTATGAAGCTCACCTTCATGTACATACAGATACAGAAGTTTTCTTTCGTCTTCAAACTTGTAAAAAGAATGAAATGTGTGTTTTCATTATTGTGATAAGTACAGCAATGTTATCAAAGACCCTGTGCAATGAGTGAAGTGGATATTATCGAGACTCTTCATAAGAGTCGAGCGTGTATTTTTTTTAAAGGAAAGAGAACATATTTTGAATTTGTAAAATATGTGAATATTTTGACGATGGGGCTCCATGATGTGTATAAGTTTTTATTTTGATCAGCATTGATGTTACATAGAATGTCCCAAGATTTGTAAGTGTATGCAGTATAAAATATAGATAAGATATCTTAAATGGGGGGGGGGGGGGGGGGAGAAAATAGTAGGTGAGAACTGAAAACATAAACGTGCTTTTTAGCAGGGTAGATTTATGATTTGAGATTATGGTTTGACTATTGCAGTATGCTTGAAGTTTCAACTCGAAGAGGTTTTTGGTGATTTCCTTCCTTTAATTGCGGGAAATTTGTGGGTTTTTCATCTCAAAGGGACAATCTCTTTGTGAGACAACCATAATATATCTTAACTCAATATGTGTGGCAATGTTGGATCAAATTTTTTTTTTTGAGAGAGAGAGAGAATTACATGAAGGTATGTAGTTCTTGAAGTGTGGTACAAAGGTTCAACAAATTTGTTGGTTTTTTTTTTCAAATTGAAAAGATCTGTGTTAATTATTTTGAGATATCTGAAATGTTGATTATTCATATTCATATGCCAGTATACATTATGTATGTGACAAACTTTTCTTTCATATAGAATGGGCCTTTTTGCACAGTGTTACGCCAGCCTTAAATTTAAGTGATATTTTAATGATAATCCATGCTTTTTCAATTTATTCATCAACTTGCTGTCATTCACCGATGAGTTAAAAACTCAAATGTTCATTTTCATGTGGAAAAGTTAATTGTTTTGTAATTTATTCATATTCTTTCATCTTTTCATTACTTTCAATGATATTAATTTAGATCGAAATCAAGGTCGTAAAATGTAATGAGGTTTTCTTCGCCCATCTCTGATATTGTATAGTGCTTTGTAATGTAAACTTGTTCTTATCCATATACAACATCGCTGGACTTGTGTCATATTTCATGCATCAAATTGTAGAAATTAAACTATTATCTCCTTGAGGAACTACATTTATACAATCAAAACCCTTTTCCGTGATCTATAAATTCAAATATTGGAATCCACATATATTGGAACATGAATTAATCCTTAATTTATTGAAACATGAATTAATCATTGGTCAATTCAGCAAACTATTTAAATTTGTAATATATTCCTAACCACATTCTCTTCAGTGAGGCAATAGATATTTATTGGTAGCAAACAGTTATTTATACATCATATTAAACATGCCACCTTTGCCTTTTATAAAATAAATTAATTCATCTGATCATTTTTACAATGTAGTCCCAACATTCCAAAAAGTTTGATATGTTTTGCCCTTGTGTTTTGCGTGCCATTATCAGATGAGAAAATAATCCATTTCCTTTACAGAAGTCACGGATGAGGGATTAATAAAAGAAAAGGAAAATGTCAAATATGTTTCTTAATGTCATTTTAATACTGATGTGTGGGTTGTATTAAACCTACACGTTAGATATGTGATGAATTGCTTTTCTTTGTGTCTTTATTTATTGAAACCTGTGAAAGGTAGTGCAAATTAATTATTATGAGGTCCATAGTAAGGTTGTATTCCATGTGTTAATAATCAGCTTCGTTTTGTTATGTTTGTGATTTTTACAGGGATTAAAACACTCTGTTCACTTTGAA

>Sp-Runt-1 peptide

MHITDVNVDHLLSSTAPLANHPSKDPVRRNNLHNSYKMAEGGQRNKASSVFKGGERSIVDALSEYPGELVKTESPNFACSVLPNHWRCNKSLPVAFKVVSLGETKDGTMVTIAAGNDENYCAELKNNTAVMKNQVARFNDLRFVGRSGRGKSFTLSIFIYTNPPQIATYNRAIKVTVDGPREPRRPKPKDQESRLMPPPIINTGHPHPFGEINPHHPNHHIGRQQSYQNQGRMPRSYPLSPTSGSYDNIQHQGQASKPWSYYNPYQSSVAQLSDTSILSAQIKTEPTELALLGQQNSTLQQYPKPDSLYPTSITRSSEVQDPRFVYPSTPAAVSSVSFTPSSMSVLSSGVESPRTILPMTPNPFPLSSQDIFSSSSTATPVTLTSPPYLPNSPPYPLYPHLYMSSPSSQTYYDSSHLPMLPSSTRPEDKQEIKHDNRPGEGIPHIPHPEMSLTVALNYGSHPQSQIELNTARTMGQSMGGMHNSGVALMQQHRNHSPVTNISPMQQSMGQMNINTTVGHLPTVDDSRKEDVWRPY

>Sp-Runt-2 CDS (genome scaffold at http://sugp.caltech.edu/cgi-bin/gbrowse/chado2.1/?name=scaffold_v2_57995%3A23199-36508) (annotation at http://annotation.hgsc.bcm.tmc.edu/Urchin/cgi-bin/pubInsertGene.cgi?gene_id=GLEAN3_07852)

ATGACAGAGGTGGGCCAGAGGAAGGGTTCGTCCCCCTGTAAGGGAGGTGAACGGTCTCTTGTGGACGCTTTATCGGACTACCCTGGGGAGTTGGTGAAGACAGAGAGCCCTAACTTCATCTGCTCTCTCCTCCCACCCCACTGGAGAAGTAATAAGAGTTTGCCCGTGGCTTTTAAAGTGGTTTCACTTGGTGAGACAAAAGACGGAACGGTTGTGACAATTGGAGCAGGGAATGACGAAAACTGCTGCGCCGAATTGAAAAATAACATAGCAGTCATGAAGAATCAAGTTGCTCGTTTTAATGACTTGCGTTTTGTTGGCAAATCAGGCCGTGGTAAAAGCTTCACGCTGTCCATATTCGTTTACACCAACCCACCTCAGATTGCTACCTGCAACCGTGCTATCAAAGTTACTGTGGATGGGCCTAGACCAAGACGACCAAAGTCAAAGCATGATGATCCTCTGTGCTTCTCATCGATCATTGACACAGGCCACGCCCATTCTTATGGGTTGGTCATCTCTCACCATGACAACCAGATTGGAAGACAGCAGCCGTTTGTGAACCAGGGGTGGATGCCTGGATCTTATCCCCTCACTTCAACTTCTTGTGATAACCAGCCTGAATTTCATACAAATCATTATACACCATCGGTTCAACAGTCAACAGGCAACCCCATCCTTCCCCCACAGATAAAGACGGAACCTCCGGAGCTGGACTTGGTCGCTGTTCAGCAGAATCAACCCCAGTTCATCTACCCGACGCAACCAGGAGACACACCCTTCTATCCTTCACAATCTAGCTCCATGTCGGGAGTGTCACCTGGGGAAGATCCACAGCGTACCCTCTTGCCGATGACACCAAACCAACTCCCCTTGCCATCTCAACCTGCGCAGGATGTCTTCTCTAGCTCCACCGCTATTCCAATGACGCCATCAGCTAACCCAATTTACCCCCACCTGTACATGTCTACTCCTTCATCTCAGACGCACTATGGTAGTGGCTCTATGCTCCCATCGAGAAGACCAGAGGAAAAGCGAGAATTGGAGGATAAGGAAGACACTAGTCATGGCATTTCCGTGGAAATCATCACGAATCTTCCTCATCCCTATCCTGATATGCCTCTAAGTTACGGAGGCCATCCGCAAAACCAGATCGAACTGGATAGAGAGAGGACCACTGGCATTCAGAGCGTTGGAATGGGGCTAATGCAGCAGCATCATCAAGGCAACTCTAACATTGCGCCAATGCAACAAACTGTCCTAGGGCATTTGCCACTTTTGGCCGATTCGAGAAAAGAGGAATTTTGGCGGCCATATTGA

>Sp-Runt-2 peptide

MTEVGQRKGSSPCKGGERSLVDALSDYPGELVKTESPNFICSLLPPHWRSNKSLPVAFKVVSLGETKDGTVVTIGAGNDENCCAELKNNIAVMKNQVARFNDLRFVGKSGRGKSFTLSIFVYTNPPQIATCNRAIKVTVDGPRPRRPKSKHDDPLCFSSIIDTGHAHSYGLVISHHDNQIGRQQPFVNQGWMPGSYPLTSTSCDNQPEFHTNHYTPSVQQSTGNPILPPQIKTEPPELDLVAVQQNQPQFIYPTQPGDTPFYPSQSSSMSGVSPGEDPQRTLLPMTPNQLPLPSQPAQDVFSSSTAIPMTPSANPIYPHLYMSTPSSQTHYGSGSMLPSRRPEEKRELEDKEDTSHGISVEIITNLPHPYPDMPLSYGGHPQNQIELDRERTTGIQSVGMGLMQQHHQGNSNIAPMQQTVLGHLPLLADSRKEEFWRPY

>Bf-Runx gene [Brafl1/scaffold_75:1906020-1923352](http://genome.jgi-psf.org/cgi-bin/browserLoad?db=Brafl1&position=scaffold_75:1906020-1923352)

1923552 ACATCTCGAGGAATCAGGCCAGAGCGGAAGCCTTCAGTTTGACCATATCCCCTAGAGTGAGTGACTGGGT 1923483

1923482 AGTGAGTCACTGAGCTTACCCGGGAGTGGTTGGCGATTACATGCTGAACAAACTCGTTGCGCATCGTGAA 1923413

1923412 ATAACCGCAGCGACGGAAGCGGGCGACACTCAGACCAGTACGCACCAGAGCAGCGACAGCCCGCGCCCTT 1923343

1923342 CCGCGCCTGTGGCCGCAAGCCTTGTAACCGCGACTACTCCGACTCACCCGCTTACTGACATGCTGATTCC 1923273

1923272 CACCCCTTCCACCCTAGACTCCAGGCGGTTCTCGCCGTTCGCGGACCCCGGGAAGATGGGAGACCCTCAC 1923203

1923202 CGCAAGGTGCACCCCCACTTCAAGGGAGACCGAGGCCTGGTGGACGCCTTGGCGGACCACCCTGGCGAAC 1923133

1923132 TAGTCCGTACGGACAGCCCTAACTTCGTCTGCTCCGTCCTACCGTCACACTGGCGCTGCAACAAAACGCT 1923063

1923062 CCCCGTGCCTTTCAAGGTGGTGGCACTGGGGGACATCCCGGACGGGACACTTGTCACCGTCATGGCAGGG 1922993

1922992 AACGACGAGAATTACTCGGCGGAGCTGAGGAACAACCAGGCCGTCATGAAGAACCAGGTGGCCCGATTCA 1922923

1922922 ACGACCTGCGCTTCGTGGGACGCTCTGGACGAGGTAGGACTGTAAAAACTGACGTACATTAAGCAGTGTG 1922853

1922852 AAGGCATGATTTGTACAAAAATCCTCGTGTCCCTGTATGTAGCTTACTGGGTTTTGTAACATCATGGCTC 1922783

1922782 TTGGGCATTTGGCCAAGTTTTACCACCATGAGTACACATACACACTTTGCGGGCGAAATCCAACGGACGC 1922713

1922712 ACCATTGTGTTTTTACCAACTTGAAGTAGTTCTTGTAATCTTTCTACTCTATCCCTTTTATGGTCACCCA 1922643

1922642 CAGCATATTTGGTGGCTAAAAACTCTAGACGATTAACTTCTGACATTTCGTAGTCTTACGTATGTACGAA 1922573

1922572 ACCACTTAGCTGTCGGTGAGGCCTTTTATTTCTCGTGTTGTGCGGGGGCAAGGCCGTGGTTTTTAATCGT 1922503

1922502 GATTGCGTGTAGGAAAGACGTATTATTGGTTTCGGCGATCATGCAAGTTTCAAACCGAAGACAGTTATGC 1922433

1922432 GTGGTATGTTATTAATTAATTGTCTAGGGATAGTATAGATGATTTTTATACAGCCGTATTTCTCTGTTTT 1922363

1922362 TGCCCAACAACGTGCATTGTTTATGTCTAGTCATACGTGGTTAAATACACGTACACAAGGTAGACAGTCG 1922293

1922292 TGTCCTTAATAGACCTCTATGTTCTTCTAACATTCAGTAGTTAGGAATGCCCAACTTCCTCGCAGGAAGG 1922223

1922222 CATGGCTGTTGCGAAAGTCACGATCACGTGAGTTCATTGCCCCTTGTCTCCCTCCCCACCCTTTCCAGGA 1922153

1922152 AAGAGCTTCACTCTGACGATTACCGTGTTTACCAGCCCACCGCAAGTTGCCACCTACCACCGCGCCATAA 1922083

1922082 AAGTCACTGTCGACGGGCCGCGAGAACCAAGAAGTAAGTGCAGTTTTCCATTTTACTACACTCTCTTGTA 1922013

1922012 TAGATTATACTCACAGTGTGGACCACATGGCTACTACTACTGGCCATGCCACGCGGCCAGTGAATGCTAA 1921943

1921942 CCCGCGCTATTTTAAAGCACCGGTGGCCCAGGGTTGGGTTACTCATACCCCATGTGACTTCCTGTGCTCT 1921873

1921872 CGGAGTGTTGTAGAGGGCGCCCTTACACGGTGGTATGCGACCCGGACGTATTGTGTAAACTCAGTGGCTA 1921803

1921802 TGTGCCTGAGAAAGTCGAGAACTGAACTGTGGGCGATATAGGGGAACAAAACATAACAACAACAGGTATC 1921733

1921732 CGTGTAAAGAGAGACGCCCCAGAATCCCACGCGTTGACATTGAGTAACACTGCGGGTACAAGCTGACACG 1921663

1921662 TCAAAGCCCAGTGTACTTTGTTCGTTTGTTGTCACCTTTGTCCCACGTCTACATGCAGACGGCTGGTTTT 1921593

1921592 GAAAACATGTTTGTGAAGAAAGAGGCAAGGTTTCGGGGCTGGGGGGCCGCGTGCAAAGCACATGGCTTGC 1921523

1921522 TAATTTGAATATAATCGCTGGGGATAGTTCACAAAACTTTTTTTGCGGTCGCGTGTACTTTTAGTACTTT 1921453

1921452 CACTTTTTACTCACAAAGCTATAAAATATGCTATTTTGTGGTATCGGATAAACGTTCTGTAGGACACTTT 1921383

1921382 CTTTTTTCGTGCCGCGTTGTTAGATTGAAAGGCTTTCGTAATTTTGCCAGCGATAGAAATGGTTTACGGT 1921313

1921312 TCTTTGGAATCAAATTATGTTGTGCGTCATCAATGAGTTTCACTCGATTCAACTTCAAAAGTGGCGACCG 1921243

1921242 TTACTCGATGAAATTGGCCGTCTTGGGGGATAATTATGACGCAAAGAACAGTAACATTACGTATTTATGT 1921173

1921172 TGTTACACTTAGTCACGAGACACGTAAGATAGTTTTCCGAGCCAAGCTATAGTTTCCTCTTCTTTTATTG 1921103

1921102 TAAACGTGAACCTTTGCCAAGTAGCCGTTTTGTGTTTGCGTAATTCCGGCCTGCTTTGGCCGTTATTCAA 1921033

1921032 ACTTTACTTACGTTATAACTGTTCTTTGTAAGCGATATTGTGTTGTGTCTGAAAAATCCGGTCCTTTATG 1920963

1920962 TTTGAAGCGGACAGGAAGAAAGAAAACACTGACGTTTTTTGCGTGGTTAATATATTCAGATACATCGGTG 1920893

1920892 ATACTCTTTTGACATAGAGCCAACCCTTTCTAACGTTCGCTTATGGCCTACTGATGATTGTTTTTGAGTA 1920823

1920822 CATGTACATTGCTATTTTTTGCTGACCCATATGTAGTTTTCTGTGGGTGTATTTGCGTGTTAACAACGCA 1920753

1920752 AAGAATATGCTGCGGTCGGCAAGGTTTTTTTTTTTGGCCGACTTTCAGGGACGGCAGTGGCCATAAGTCA 1920683

1920682 ATTATTACCCCGTTGTTTTTCCCGCCAATCAGTTTAAAAATAGAGCCAGGACATTAGAAACTGTGCATGT 1920613

1920612 CAACAGTATGACAGCCATGTGATTGTAGCGTGATATTGTGCAGGTGCCAACTTAGAGTGACTTCGTGAAG 1920543

1920542 AGACTTTAACCTTCGCCGGTGCTTTTAACACGAAGTAGTAGCCTTGAGAGAGAAAGAAAGCGGTGTGCGC 1920473

1920472 CGGCCGTTGCCCTCTCGGGCGTGTGTTTTGAAGTCACCTGGCGGTATGTTTACAACTTCGGCTGTGACCA 1920403

1920402 ATAGATTTACTCCAAAACAAACACCAGAGGATTTGTTTGCATGCAGGCGGTTGAGCGAGCGGGGGGCATT 1920333

1920332 CATTCACATGTTTACAGATAGTGTCCTCAACCCCGCTCCCTTGTTGAGTGACATCTGCCCACATCGAGTC 1920263

1920262 TTTTATGGTCCGCTTAGGCGACAGCGCGGTGAAATGTGCACACTCTAGACCTGTGTATTAGTAATGGGTA 1920193

1920192 ATTGTTTCGCCCTTGTTCAAATGTCAAAGAGGGTTTGAAAGTTGAAAAGACGCCCATGGCTAGTAGGTGT 1920123

1920122 GAGATAGTTATTGGGGAGTGAGCTTACACATAGGTGACAACAAGTTTATATAGAGTGACCCGGTAGGTTA 1920053

1920052 TTGTTTTATGGACTTGTCTAGAGCGCCGCCCAAACAATTTCCATTCACTGTATTGAGGGAAACATAGTTT 1919983

1919982 GTGTGGGAGAATATTATTGTATTTGGTAAGGAAAACAAGGGGAAATGACTTTGAAATCTAAATTGAAATA 1919913

1919912 TATTTGGGTTTGATATATTTGAGTAATAGATTTATAAAGAAAATAATTTTCATTTGGAACAAAAAGTTAG 1919843

1919842 AATCATGGATAAAACTTACTTTTTAATATCTAGGAAAAAGTAGCAAATAAAGTAAAAGGATTTTACAATA 1919773

1919772 TAGGCCTAAATGTGTATTTTTTCTTATAAGATTTTCCTCAGCACAGGAACACTAAAAAAGTAAACAGATC 1919703

1919702 CTTCACATTTTCCAAGAGTAGGACTTTCAACCCAGAGCAAAGGTTACTTTTATACAAACAATTGTACAAC 1919633

1919632 AAATACACACACACACACACACATGTACACAGGATTGAGTAAAACACTGCACTGGACCTTTTCTGTTGAT 1919563

1919562 CTTTGTAAAAGAGTAAACAATCCAGTGCTTATCAAGTTTTGGATAATCTACTAATAAAGAAGTTTCTCTC 1919493

1919492 TGCTGTGTCCCTGTAATAAGGTCATAACAGGGTTTTTAATGCTCTAAGGCAAAACAGTCCACCATACTGT 1919423

1919422 AAAGGTAATAAACTACTGGTTACAAAAACACTTTGGGAGTCCACACATTTTGCCAGACCAGGTTGAAACT 1919353

1919352 TGTCTAAAACATGTGCGAAATGTTCAACCTCTATTGTTTGACCTTGTAATCCTAAGGATTTGTTGTCAGC 1919283

1919282 GACAAAGCATCCCTTATTATACCTGCTACAGGTACAGGTGCACCTGTGCCATTAAACTAAGTCATTGTTT 1919213

1919212 TGTTTGTTACTTGATCCCTGCAAATTAATAGTCCAAACTGCCGGCAGAAAACAAGCCATAAAACTGAACA 1919143

1919142 ACTTTGAGGCATTTCAATCTCTTACACAAGGAAAGTCATTTTTTAATTTTTCGAAAAAGTACCCCAACCC 1919073

1919072 CAAGTTTTGAGTCTTGAATTGTTTTCTATTTCTGTCCCCATCCCAATTGAGAGAATTGTCTGAATGAGAG 1919003

1919002 AATTGTCTGTATTGTCAGATCAATGGTTGAAATGTAGTGTAATGATAAAAGGTAGGGTGCACGTGTGCTG 1918933

1918932 CGGCCTTGTTGTGTGTTTAGCCCACAATAGCCCGGTTATTCCAACGACGTACCCATACCGCACGGATAAT 1918863

1918862 GTGTGGACGCAGCCTGCACGTGCCGACCAATGGTGGCTCTTTTGTCTCTGAACAGGTGATAAAGCTGGGT 1918793

1918792 ATTATTCTACCCATAATTGAAACTTCCTTTGTGCGCGGTAGGTGACAGGATTCTACTGGTGTGCAGCAAT 1918723

1918722 ATATCTCCAGCATGGTGGCTGCCGAACAGGTGTAATTGGTGCCCCTGGCCTGATTAAATGCTTCCCTATA 1918653

1918652 GTAAATAGGCCCACCAAGGTGCCATTGTTCCAAGCTTTACATAGGGGGATCTACAATAAGACTTAAAAAC 1918583

1918582 TGACAGATAAGCAGACTGGCCATGGCATGGTGGTCAGGGCAGCTGTACAAATGGCAGTATTCAGACAATA 1918513

1918512 CTGTGTCTTTAGAACCCCCATAACTTTTTATGAGCTCAAACATAGGTGTCACTGTTACAATCATAGATCT 1918443

1918442 GATGGTGGTAGTCAGTTATACTTTCCTTTGTATGGAGAAAGAAATATCAACTCAAATTCTCTTTGACGCG 1918373

1918372 TAAGCCAGGTTTTACGACACAAGGTGTTCTTAAAACTCTTAATACATGTAGGTGTCCAAAATATAAGTAC 1918303

1918302 CCACTGCAGGTGTTGAAAACCTTTAGTGACGCCAACGCCACAAGTCAAGGCTACGATTTGCTTGTCCAAG 1918233

1918232 GTGTGTCTGACAACAGATCATTACCTCAGCAGGTGGTGCAACATGCAACCTTACAATCTACCTGTCTCAG 1918163

1918162 CTCTGCCCTCACCTGTTCTTCGATAGGGGACTAATAGACCTAAGGTTTAATTAACGGTTAGCGCGTGCAT 1918093

1918092 TTACAGCAAAATGTCCAGTGACACCATAATTAATTTCGCCCCGCGTCCTGTGACCATGAAAAGTGGCAGT 1918023

1918022 TTTGAAAGAGGCATCCGTTGTCCACATTTTTCATATCAGAAGACATAAGATGACGATGCAACTAATATCA 1917953

1917952 CATGTGTTCAACAGAATACGATATGTCCTGCCCAAAATACGCTTCAAAACTGTCAGTTTTAAAAAGCTCC 1917883

1917882 AGTTGTCAGTATTTCTCTGTATCGGATTACGGTAACAAGATTATTGGCTGTGCTGTTTTGTTGTCCCTCA 1917813

1917812 CCTGTACTCCTAACTAACATAATCCCCAGAGGGCACCAGGCTGAATAGAACAATAGAGCTGACAGAAACG 1917743

1917742 CTAACATACTGTCACCTTCAGATTTAATCGTCCCAGCGTATCATCTCATTTCTGCTTGTAACTGTCACTT 1917673

1917672 GTGGCCTATTGTGTGCACATTTTGACCACCTCACTAACTTTTGTCCATGCTCTATTGTCTCCCCTCTCTG 1917603

1917602 TAGCCCGCTTCCATACTGGTAGCTGCCCGTATACCGACCTAGGCATGGCCTTAATAGGCAAGTCCTCTTC 1917533

1917532 CACTTATTCTGTCACTGTGTCCTTTTGAAGTTGTTGTCGTCACCCTCCCTTTGTCGTTCTGTCACAGTTT 1917463

1917462 TTTGCCTCCTCCCTTCATTTGATACTTTTGAACTGACATATAAAATATGGCAATGATTTGAAATATATAG 1917393

1917392 AACTGTTTTAAGATCCTTATTGTCATTCGGGTCCATTCCAACTACTATGTAAACTTTGATGCTGGGGAAA 1917323

1917322 ATTCTGCTTCTTTCCACAATAGACATACATACATTGAGAAAACATATTTGTGGTCTTGACAGTATTGGTG 1917253

1917252 GCACGTGTGACACGGGACGTGCTTGCTAATTAGTAGTTCTAATTGACGGGACTTCCTCTCACCTGGACAG 1917183

1917182 GATACCTGGGCACTCCCATCCTGTTGCACCCACAATATGCCCCAATGACCGGAAACACATACCTCCCGAG 1917113

1917112 TACAATTTGTAACACTTCACTACTTTACTTTGGGGCTGGGTCAGAACCTTCTCACGAGGTATTAGTATAG 1917043

1917042 CCCTAAAGCTATGGAAGTAGTTGTCACCTCAAGGCATTGCTGACGTGGTTTATGTACAGGGCATCCAAGG 1916973

1916972 ATACGCCTAGCTGGGTCTGTGGTACACCTGGCTTGTATTGTAACGTTGAAGTAAACAGGTTTATTAGCTA 1916903

1916902 TCTCTCATTAGAAAGGAGACAGGAAGAATGTCGTACATTTTGTCATATTTTGCCAGTTGCTATTGCTGTG 1916833

1916832 TGACGCAGAAGTATACAATATTTTGATGTCAACAAATTGGGGGTTGTGTGCTATCAACTCATGAGGAAGT 1916763

1916762 GTACACATACAACTATTCTCAGTTGCTGCCAAATCCCAGTGACACTAAATTCATACCAAGAATTTGCCAA 1916693

1916692 TGGTACACCAGTGGAATGAAGCCACCACTGTGTAACCACCCCCACCTCACGGCCCTCCCACACACTTTAT 1916623

1916622 TGTCTCCCACAAGTAACATACGCGCTGGGACTGTCGGACCACCACCCCGCAGTATAAAGGCCTTAATCCC 1916553

1916552 ATGGTGGGATGACACTAAGAAGGAAATCGATGTCCCCGCCCGGGGTTTACAGAGCGACGGATCTGTCTTG 1916483

1916482 ATGACCTAAACAAACACCGCATTTGGTAAATCTGGTTTACCTTGTACCTAACGTTCCCGATCCCATAACC 1916413

1916412 CAAGGGGATCAACTTGGCCCTCCCTTCTTCGTGTAAAGACAATCTATGGATTATGATGGCTAAAATACTT 1916343

1916342 TACAATGAGATTATGTGTCAGTCATTAGCTTCTTGAGAATGATGGATCGGCCTCTTGTCGAAAGAACTTT 1916273

1916272 GGAAGTTTCGGGGAATTAAAAGTTTGAAAAATCGAAAGGTCTACCGGAAGGCAGCTCTCGCCATTAAACA 1916203

1916202 TTCATAGTACTTCTGGGAAAGATGCTAAAATCATAATCCTTTCTTGGCTCTCCACTTCTTAAGAGTCTTA 1916133

1916132 TGTTTTATGCCACTTTGGCTAATATTCTGATATTACTGTCAGAATATGATGGATGGTTGTGACGGGAGGT 1916063

1916062 GGATAAAGCATATCACTCTCCACACAAATTAGATCACTCACGCCAGTGTCGGAGGCTGGAAAGACGAGCT 1915993

1915992 GATAGTTTTCACAGTTTGTCATCACTTTAAAATCGAAGGGTCTGACCCGACGACATTAATGACCTTAGAC 1915923

1915922 TGACAGAGTTAGATGCCAATCATACTCTTCCTACAGGTCCACACGGGTGCGCAACTTTGGACTTGGTGAG 1915853

1915852 CTGAAATGGCCAGACAATAAACCAAAATGACAAATCCTCTCTGCAGCAGACTAAGGCTTTAGATTTGGAT 1915783

1915782 TTGACAAAGATCATCATCCATAATGTGCTTATCCAAAGAATGACATAAATAACGACTGTCGCAGCAGAGT 1915713

1915712 TAGACAGGTGTGCTCTGAACACGGCATTAAATAATTGAAGAATTACAACATCGCCATATTAAAGTTGATT 1915643

1915642 ACGTTTAGTGTAATCTACCATGATTATCTGCTTGAAAAAGTGGGTTTATCATTCCTCAAATTTCAGACCA 1915573

1915572 GAACAAAAGAGCTGCAAACGACTGTCAATGTTTCAACTGAAATGATTTTTCTTTTTTTTTGGTCGGAGGG 1915503

1915502 TGACCACCAAAGTCAGTACATAGTCCCTCTGAGTGTAGGTCATGCAATGTACCTATTAGGCTCAACTGCT 1915433

1915432 GCAGCTGTGTGGCTTATAGGCTCATCTTCACAATGGGGACAAAAAGCAGGATTTCCTCGGGCCTTGAAGT 1915363

1915362 TTTAGCCCATTTCTGAGGCTCTGCATTCAGACTTTTGACAGGACCCTCATCGTGGATTACGTGCTCTTCT 1915293

1915292 ACTGGCCGATGATGGATGGCCTGATGCAGGGATCATCTTATCCACCATGCTTCCTAATCGACTTTACTCA 1915223

1915222 AGCCCCTGCAGAACAGTAACTGTAACCCCTGTTTGACAGCGTTACCGTCCAACAAGGACAATTTCTGTAC 1915153

1915152 TTTCTAAGGCCACGATAGCCATTTTCCTCTCAAGTTTGGTCCCAGGAAACACGTAGATTGTCTCCTACAA 1915083

1915082 AAGGATTTGGTCATTTTGCGGAGATCCAGCCACCTGTCTCGTTTTGTGGTTATCGCTACTCCGAGTATGG 1915013

1915012 CACGGAGATGGTACCACCAAAGTGGGGTGTGACAAACAGCTGCTGTCATCCCCATGGATTATTCACCATC 1914943

1914942 TTCTGAACTTACCCTCATTCATTACCCCATTCATTGTTAGCTAAGACTGGGTGGAAAGGTTTCCCCCAGG 1914873

1914872 GTTAATGTGGGGTAGACAGGTAGTTACAACCACCAGGACTTGACTGCTGCTGCGGCTTGTAAAAATGAGC 1914803

1914802 ATCAGCCAGCGCTCCTGACAGCTGAAACCTCGGCAGACTCGCGCCGCCCGCCACCCTGACGCCAACCTCC 1914733

1914732 CCACAACAGAGTCCTTGGGTTATGGTCACGCGAACATGTACGCCCGACCAACGCCACACATCCATTGTGC 1914663

1914662 AGCCAAGATGGAAGGTGTGGTGTCTGGGTGACCGCTGACGGGCGTGCGCTCCAACCTCCAAACCAATTTC 1914593

1914592 AGACCAAATCACTCTGTCTATTTCATGAAGAAATCATGACAGCCATTGTTTGAGACGAGGCAACGCCATT 1914523

1914522 GTCTTTCAGTATCGATCGTCTGCTACAATGACATTATTTCATCCCAAAGACATAACAAAAACGTCTTTAT 1914453

1914452 ATAAATGTGTCTGATGGTTGAAATAGTGACAGTTTGTCCCCCATCTCGACACTTTGAATCTGTCAATCAA 1914383

1914382 CACTGAGGTAACCAGAGGTTGAGCTGGGATCAAATGAGGATAAGGACTTGATTGGCTGGCTGGGGTGAGA 1914313

1914312 TTACAAAGACAAACGAAGGATGATACATTGTGACACAATTCTTGAATAAAAAAGAAAGGACCCGGAATAA 1914243

1914242 AATAAAAAATAAAGAAAACTTTTTTTGTTCATCCAATGGAGCAACATATTGTAAGTGAAGCAAGGTGTGT 1914173

1914172 GTCAAAAAAATTGGCAATTGTTTCTTCTTACAAGAGTCATTTACTGTACACATTTTCAGGTTGATTTAGC 1914103

1914102 TAATTTTCTTGTACCATTCCCCTTGAGAGATTTAACCTTTTACAGCACAGCAGTTAAAAAAAGTAAGTTG 1914033

1914032 ACTTAGAGATGGGTACAACTTTCTAAGTGATAGTTGAAGTCTATAAGCACGCACTCTTGATTTTTTGGCA 1913963

1913962 CAATGACATCATTGAGTACTAGACAGCCACCGTATTTGTATAGAACACATTTACTAACACAAAAATTAGC 1913893

1913892 CATGTAACTTTGAGTCACTCTTTGCAATCAGCATTTAAGCTGTCAGTGCAATCTCAGAAGGGATCTATCA 1913823

1913822 GGCAGTGCTGGCATGAGAGCCGCTGCCTCGCCACTCATCATAATCAGCACGGCTGGGGAATCGCGTCTGC 1913753

1913752 TCGCAGATATCATCAGTACATCGCATGATGTGAACATAATGTGATTTCTGCAGCCCTTTCAACCCTGGGC 1913683

1913682 CTGGGAAGACCTTGTGTGAGTAACACAATGTCGGGAGCTTAACGTTTCGTCGTTGGTGCAATCATTGATT 1913613

1913612 CGGTTGGGCAGCCTCGCGTGAGGAATTAAAATGGGGGTCTCTATCAAACCTCTCCTTGGGCTGCCGGTGG 1913543

1913542 CGGGACTTTTGGTCGTGAAGCGCGCAGCTGAGGTGGACCGATTCTGACAGGCATACACGGGCAAACATAA 1913473

1913472 TAAATTGTGGTAGATATGGATGGTGATTGTCACCGAACACATGCCTATAGTTTGTCATAACTGCATATCC 1913403

1913402 ACCACTTTATGAAATTATGTCAGCACTCAAATGGGGGGTCACATGTGTCAGGGTGTGAATCAATGCTAAT 1913333

1913332 CGCATTCACAAATATATGGATACATCATATTTTCGGTCACCCCTTCCTGTGAAAGTTGCCATTTCCACGG 1913263

1913262 GACCTACGTCAAAAGTTTTCCATAACTGGGTGTTTAAAGGCTCCCCGGGGACCCCTTTTCGAATTAAGGC 1913193

1913192 GCATTCGGAGCAATGAAGTTGGTTACTTCCGCACCGCAGGTATCACTCCTCCGCCAAGGGCACGACAGCC 1913123

1913122 TTTCAATCACCAGTCCATCAAATTCCAACAAAAATGTAAAATAACTTCCAAACGAGATTAACCGGCTGGT 1913053

1913052 GTAAAAGCAGGTCCGTGGTGTCAAATTACCCATGTCGCACACAATATCGTACAATGTTCCGCTGCTCATC 1912983

1912982 CCTACCAGTTATTGATCTCTGATCTGATTTGTCATCTATTTCTGGTGGAAATTTTCTTCAAAGGAGTTGA 1912913

1912912 CTCCAAGTTCAGCTTAAGGCTTTTCCGCTGTGCTAACCTGCAAGCCGAGAAGACGGATTGGCTTGGTTAT 1912843

1912842 TGAATCATTCCTTGTGAATTATGTATAGATGGAATATAATGGTAGTGGGCAAAAGTCACACACTCCTAGA 1912773

1912772 ACAGAGGCCTGGGGTATATTGAATATTTCATTTATATTCTGCATGTATTTTATCCTAACAAGACTTACAC 1912703

1912702 CAAATAAGTAATTTTTGTATTGAAATAAGTAATTTTGCTAATATTGTCTCTTTTGTCAATCGTCGACAAA 1912633

1912632 TGTCAGTCAAGTACATGCCATCCATGTTATGAAAAATGGCACTTAAGTGTCATTTGTTTTGATTATCATG 1912563

1912562 CACTCACTCACAAATAAAATTTGCCTACTTACCAGTCCTCACCCACTGATGGAGAAATGTGAATCTAGAC 1912493

1912492 CAATAATACATTTAATAACTGGGCGCATTATGAAGTTAAAGTTGTTACCGGCGTGACAGTACCTCAAAGC 1912423

1912422 TCTTACGGACATGGCGCGACATTTGACACCAAGTCAAGGAACACGTCAGAAGGAATATAGCTCATATGCG 1912353

1912352 ACAGAAAGCAAAAGTTTACAAAAAGCCATACATTTCAATCACGGTATCGATCGCTCGATGCAACTCCGCA 1912283

1912282 AAACTTTTCCTCAGAATTGAGTTGCCGCCCCTAAATTCACATGCAAATCAAAATTCACTGTCAAAAAAAG 1912213

1912212 GCTTCTTGGTGCATTTAGAAAGTACATACGTTATCTGACATATTCAAGCATCAGATTAATGTTGACTTTT 1912143

1912142 CAGTCCTCAGTGATGATCCCTTTTGTGAGAGATTGAATTTTTTAATAGTAATGAATTTTTTCATGTATGA 1912073

1912072 TATTAATAATAACAATGCAACAATGTCTTGATTGTAACGGAAGCAAATACCAATATGGACATTGTACTAA 1912003

1912002 AGAGGAATATTCTATTTTCTTCAGGGCACCGACAGAAGCTAGAAGAACAGAAGCATGCGCTGTCTTTCTC 1911933

1911932 CGAACGTCTGAGTGAGCTTGGTCTGGAGCGACTCCGACACAGCCACATCGCCCAGCCCCTGCCTCCTGCC 1911863

1911862 ATCCGCACTCCGCTCATAGAGGCGCCGCCCGGTCACCATCCTCCGCCTTCAGCCTACACGCCCGGAAACC 1911793

1911792 CTCAGATTCAAGCCACACAAGGTGGGTCAACATTTATGAAGACACAGTACTAGTACATATAGCAGTCTAA 1911723

1911722 AGATTCTGGCCACTTCAATCTTGGAAAAAGAATAAAAATCATAATCTTTTAGTTTGTTGATTACGACAAG 1911653

1911652 ATTAAGGATTAAGATTCAAAAACATAGTTTTATGCTTCCTGGTTTGCTTGGGGGTTAAAGATTACTACCT 1911583

1911582 ACACATCATGTAACTGTAAACAGCATTCACCAGGCTTTCCCAGAAATCAGAACTGTTTTCCACTAAGTCG 1911513

1911512 CCTCAGGATAGGAAGATTTAGTTCACACACCTTCCCTATTGTTAGCAAGAATGTTATTGGTGAAGGTTAC 1911443

1911442 AGTGCTGGGTGGAGTTCACATAGTTGTGGTGGGGCAAAAGTGGTTATCGCTCCACTGAACTCTGGGTAAC 1911373

1911372 TCATTGCGGTTTACTGGGGAGTTCACCAAAAAAGTTTATACTGCTTTGGCAAAGTTCACAGTAAAATGGC 1911303

1911302 CACACTACGAGTGAAAGCTACGCGTGACTGTGGCAAGCAAGTGGTATGCCAAGGTTCAGGTGAAAACAAT 1911233

1911232 GGAAACAATAAGCATGAGGTGTGAACATGAACGTGACATCCTGTGGGAGTGTCCAGTAGACCAGTACATT 1911163

1911162 TCACACACCCAAGAACAAAAGAGATTTTGAGGTTGACGTTTAAGGTCTCGTGGTAAATGCTGACAACTTA 1911093

1911092 AGTAGGCGGAAAAGAATGCAAAGTAATTGTTCATGAGGAATTCTCAAACAAGCTTCCTCAACATTCCCCT 1911023

1911022 CGAGTCTGACATAGTTCAAGTCATCTTCCTCGATTTTCCTTCAAAACAGCAGCGCCAAGTACCAAGTTCA 1910953

1910952 ATTCTTAACATTAACTTGAAATTCATCCCTTTACTAATTCAATGCAAAACATGACATTATATACGTTAGC 1910883

1910882 TGCAAGCTAAATGTACTTGACAAGTGTAATGCAAGTTGAGCCATTTTTAAGGGAATGTGAGGATTTTTTC 1910813

1910812 TAAGAAACACCACCACATTTAAGGGGACTTTGGCAATGCTCCAACATTCAGACAGGTAGTACACCATTCT 1910743

1910742 TTAAGGGTTGGGCTCAATATGCTATCTAAGGAATGAGGAGAATCCTCTTCGAGTCAAAGGAATGCAGGAT 1910673

1910672 GGAGGGATGGGGAAGGATTAGTGGCATTTTCAGCCACAGTAGATTTATAACAAAGGAATGTTCATCCCCT 1910603

1910602 GGTATAAGGAAGTACACATTTCCATTCACACAGGGACAAAACATGGCTCGCTCCCATTAATAACAGATCA 1910533

1910532 TTGGCAAGATAGCCTACTTTAATGGTTAATGAATGATAAGAAAATGTCCAATGAGGTTCCCAAATTTGGA 1910463

1910462 CAGCGACAAAGGAACTCTAACCCGTCACCATTTACAGAATGGAATTGCATTTTGCAGTTCTTTGGGGAAT 1910393

1910392 GAATAAGATACTAAACAATCTAATCTTTGGGCCTGTATTGGATTTAGTGCTACGCAATTACAGTAATTAA 1910323

1910322 TGTCTTGAGGTTTACCAAACATCATTGTGCACACAAATACGAGGATCTTTAATGTTTATCCACAAATGAG 1910253

1910252 AAGTAGCAACTCATGGAGAAACAGAGGGTCCCTGTGGTGCTCTTGACAAAAGGCCAAACTCTGAAATCAG 1910183

1910182 GTGACTCCAATGGATGGATGCCAATCAGGATCTGCATACACAGTGGAAAAACAAATTGTTGGTCTCCTTC 1910113

1910112 CAAGTTTCCCTAGAACAAACACAATCTAGAACCTGGTTACCAAGAGTTGTGCGGTACACAACCTCGCCGT 1910043

1910042 GTGGCAACATTGTTGAAAGGATTATCGTTTCTGAGTATTAAGTGTCGGCGGCCTCAAACTGGAAAAACGT 1909973

1909972 GCGCCGGCCTGGCGCAGGTGAAACACCGCCATTGTTTTCTTATCGGTTGTGTGCGAAATGCTGAGCCGCC 1909903

1909902 TCGCTGTAACCACAACACAGAGACTTTATCTCCTGGTCGGGAGCAGCCCAGGCTATGTGATACCTGTTGT 1909833

1909832 TTGTATTGCTGCCCACAAAAGGCTCTTGAGGTGGTCATGTTTTCCCTGATCCCATTGTTTGAACATGGGC 1909763

1909762 GGGCGAGCTGTTTGTTCTCGGCACCGGCTTGGCCTTGGTACAGATGTGGAGATTTCCATCCCAATGGAAT 1909693

1909692 GTTGTTGTCTTCTAGTTTCTGACAGGACGAGGGTCTCAATGGGGGTTTAATTTGAAATAACTCTTTTTTA 1909623

1909622 TAATCAAGTGGCATTGAAAAATTAGAGACTCAGCACATTAACATATTCTGAGATCACGGAAATTCCTGAC 1909553

1909552 ATTTGATTCCATCAACAAATTCTTAGATTTTAAAAAAGTCATAAAAAGTCTACTTTAAGTTGCTGAAGTT 1909483

1909482 GAATGTATCTTGTTCACAAGCAAGCCAACATCTTGACACTAGTACTAGTAGGTAAGTCATATCCAGATAC 1909413

1909412 ACTAATACTCCATACTGACCCCCAACCTGTCATCACTGCCAAGGTTTTCCAGCTTCCGTTCAAAGCAAAC 1909343

1909342 ATCCAGCTGGCATCCACGGTGGAGCCAGATGGTGACCTTAGGTGATCAAAACAAGAAGGTTAACCGACAA 1909273

1909272 ACAGCTCTCAACTCTGCCAACCAAAACTTTCACCCTGTATTTTGTAAACACTTTACAACACATGTCTATC 1909203

1909202 ACCTTCGTTGTCACTTCTGACAAATCATATCAAAGATTCAGCCATTGAAGTTGAGATCAATATGTCAATC 1909133

1909132 ATCTGCTGATGTCAAAGCTCTTCTTAAGGTGCATTCTTGCTGAGATTGTGACATATCCGTGGACAAGGCT 1909063

1909062 GTCACAATCTCCTTACCGTACCCTAAGCCTATATCAGACTCCCAGACTAAAGGTTATCACGAAGAGGAGC 1908993

1908992 CGGGGCAATTTTCAGTGTCAGGGTCGTGTCTAACGCTCAAGGGCACAGACCTTGGGGCACATGTGCCACT 1908923

1908922 TTCATACAGAAGTTACCACTCGGTATGTCAATTAGCGAGGACTATCTGACTATGGCAGGGAAAGAATGAT 1908853

1908852 GTGTCTACAGTTAATCCGACACAATAAATGTAACAGGAAGTTGATGACTGTAATGGGATGGGTTATCTCC 1908783

1908782 CCGACTCGGGATCGCTAGACAGGGTAGCGGGTATCGCGACCCCCAATCTGACCTTGTGATGTGATAGATA 1908713

1908712 GGTTTTTGATGGATGTGTCTAATCTTCTCATTTCTCTTATCTTGTCCATGTAGATGGAGCATCCCGGTCG 1908643

1908642 CCCCCGTCGTCCTGGCCGTACCAGCCGTATCAACCCTTCATGGGCGCTCCCATGCCCCCTCCCGCCATGC 1908573

1908572 CACCTCAGACGACGGCAGCGGCGACTATCGAGCGGATTTCTCCCGAGATGGCGGGCCGCCTCCCGACAAC 1908503

1908502 CATGCCCGAGGTCATCTCCCAGAGGTTTCCCGGCGACATTCCGCTCCGCTTCCCAGCCGATTCGCAGTTC 1908433

1908432 TCGTACCCGGACCTGCGGTTTTCCGACCCGAGACTCTCGGACCCCAGACTTCTGTACCCCGCAGCTGGAG 1908363

1908362 CGACCGGGTTCACCGCCTACAGCAGTGGTCCCACCACCACCATGTCCATGCTCCCCACTAGTCTCACCAG 1908293

1908292 CCCCCGGTACCTCCCCATGTCCCCCCCGGGGTTCCCCTCTCTCACAAGTGCCCCGGGCGGATTCGTCACC 1908223

1908222 AGCCCAAACTCCCCTCCCCGGCAGCTTGGGAACTACTCCCCTGGCTCCCCTCCCTACGGCATCTACCACC 1908153

1908152 ACCTCTACGGTGGGTCGTACCAATACCCCATTCTCCCGGGGGGCAGCGGGACGCCGCAGGGGCCAAGGGA 1908083

1908082 ACCCAGCATTCTCGCTGCCACGTCCAACTCTCAGACTCAAAAACCGCAGTTGTTGCCAGCACAGGAAAAG 1908013

1908012 GAAGCGAGCAGCCAACCTGGCAACGGTAACCATGGAAACAGACAGCAAGATGAGCAAATGGACACCCAGC 1907943

1907942 CAGGGGGGTCACCAGAAAAGCGAGAGACAGTCTGGCGCCCGTACTAATTCTGTCATCGCACGTCCAAAAT 1907873

1907872 TGAATTCTCTGACCAAATGATTCCATAAAGCCACGTGGCGCCAAGAGGGAATTCAAACTTCCTTTTTTTA 1907803

1907802 TTGTGCAAAAACCATTTGAGAGTATTATGCCATTGGCAAAACTGTATAGAGCTACTGTATTTATAAAGGC 1907733

1907732 ACAAGATTGGTGGACAGTATAGCACTCGTATCAATGACAAGTACAAGGGTGTCATTGAAGGCAAACTGTC 1907663

1907662 TTTAACGTTAGACTTTACAAAAGAGGCTAGGATCCTCTCAACTGTCAATTTGTCAGTCTGTCGTGTACAG 1907593

1907592 TGTATCAAAATAATTATAGCCTCTACGCCGTGATGATTTCGAACAAGATACCAATCAGAAATTTGGAGGC 1907523

1907522 AGAGAATTTATCGTGTTGTCGTTTTCAATCATCAAATGATTTGTCGAAGACCAAACTGATGTGTGTATGC 1907453

1907452 GTGATTTCTAGACAAAATAAGTAGATGGGTGTAGGGAGCAAGTATAGGTGCATAGAAGGTTTCATCATTG 1907383

1907382 TCACTTATATTTAAGTCATCACTGTCATTAGTAATTATACAAAATTAATGCACAATTGAAGTTTAGCGTC 1907313

1907312 ACTAATTACTCAGTAATTGATGAGATTTGGAACTGACATTTTGGGGTTACATCTTTCACTACTCCTCGCA 1907243

1907242 ATTTTAACATATTAAAATCTTATCTATTTCATGTAACAAGATGATTAAGTGTCCATCCCATAGGAACTCA 1907173

1907172 TTGGAAAAACACTGATCAAAACAATGAAGTTGTTGCAGAGGGAGTGGACTTTAGACAGTTTAGAGTAAGA 1907103

1907102 TTGTTTGTCTTGTCTTTTTAAGATCAAAGTTTGTTTTCTAACCAATAGAACAGTCTTTATTTTAACTGCG 1907033

1907032 ATAATTTTCTTTATCAGTTGAAATGTTTACAGATCCAGACGGTGTGTGGTTTCAATGGGTACCGTGTGGT 1906963

1906962 AGCTACTTTACAAATGCAGTAAACAAATCAATGTTTTGAATGTAGACATGCTAAGTTAAGTCAATGACCT 1906893

1906892 AATAAAAAAACTGGTAAGAGCTGAGATCAAATGCTGTGATATAAAAATATAGAAAACAAACACAAAATAG 1906823

1906822 ACAGACAAGTATTGTACATCATAAGCTTGTTTTAACGACATACATAAATATGAACATCTATCAGTAAGAT 1906753

1906752 TTTAAATTCTTAGAAACCCACTAGATTTAGGTGTTATTGCAGTGTTGTATTGGTTTCTTTGCTGTATCAC 1906683

1906682 TTGGTTATACTAATACCATATACTAAAAATGACTGTCTTTTAAAATGTGTCTGTATTTTCTTTACTTATT 1906613

1906612 ATTAAGCATACATTAAAGCTATTGGTGTGTGCGTGTGTTAAGGAAGAGCACATTCCACTGTTGCATTACC 1906543

1906542 ACTTCCTTCATACCAACCTTACCACCCCTATCTCTGTAAATACTGTGGTCCACTCACCTTATATTCAGAC 1906473

1906472 TGAACCAAACTATCTAGAGGGGGTTCATGTTGAACAAATATTATGTGGCAACGCATCAATGCAATGGGTG 1906403

1906402 AATGTGTCAGATGGTCCGTTCTGTTTTTGTGCAATCTTTCCTTATTCCTTTGACTTATATTCCAGATGTC 1906333

1906332 TTAGTACTTAAGAAACCACATCACAGTATGCTCAAGATGCAGGATTCCCTACTGTCTTTTCTACATAGGA 1906263

1906262 GACCAACTTGCTGTACCAACAATATCTTGGGTGACATAGAGAGTTAAAACTGCTGTATAGTGCACAAAAA 1906193

1906192 CCTTTAGGAAACCTGTATGTTAAATCGATACAGACACTTGTACCGTTACGGTGGTGTACTTTCATATCTT 1906123

1906122 ATATGTTTCATGCTAGACAGAGAGACAAATAAAGCTTTTAAGATATTTTGTTTTCGTTTGCAGTAGAGTC 1906053

1906052 AGAATTGTTTGACCTTCTCAGAATAAAGCAAATATTATGTATACCATGAATGAGCTGTGTTTTCATCTGC 1905983

1905982 CAGTATGTATTGTCTAATTTCTTTCACATCTCAGTTATACATGTAACGTTTGTTCACCTTTATCCGTGGG 1905913

1905912 TAACCAATATCCGTTGCTTTTTAAAACAGGGTATTTGAGGATATTAAGTGGACAGATTGTGTTTTTTAAA 1905843

1905842 TTGCAATATTTTCAAAATATTAC 1905820

>Bf-Runx mRNA

1 CCGCGCCCTTCCGCGCCTGTGGCCGCAAGCCTTGTAACCGCGACTACTCCGACTCACCCGCTTACTGACA 70

71 TGCTGATTCCCACCCCTTCCACCCTAGACTCCAGGCGGTTCTCGCCGTTCGCGGACCCCGGGAAGATGGG 140

141 AGACCCTCACCGCAAGGTGCACCCCCACTTCAAGGGAGACCGAGGCCTGGTGGACGCCTTGGCGGACCAC 210

211 CCTGGCGAACTAGTCCGTACGGACAGCCCTAACTTCGTCTGCTCCGTCCTACCGTCACACTGGCGCTGCA 280

281 ACAAAACGCTCCCCGTGCCTTTCAAGGTGGTGGCACTGGGGGACATCCCGGACGGGACACTTGTCACCGT 350

351 CATGGCAGGGAACGACGAGAATTACTCGGCGGAGCTGAGGAACAACCAGGCCGTCATGAAGAACCAGGTG 420

421 GCCCGATTCAACGACCTGCGCTTCGTGGGACGCTCTGGACGAGGAAAGAGCTTCACTCTGACGATTACCG 490

491 TGTTTACCAGCCCACCGCAAGTTGCCACCTACCACCGCGCCATAAAAGTCACTGTCGACGGGCCGCGAGA 560

561 ACCAAGAAGGCACCGACAGAAGCTAGAAGAACAGAAGCATGCGCTGTCTTTCTCCGAACGTCTGAGTGAG 630

631 CTTGGTCTGGAGCGACTCCGACACAGCCACATCGCCCAGCCCCTGCCTCCTGCCATCCGCACTCCGCTCA 700

701 TAGAGGCGCCGCCCGGTCACCATCCTCCGCCTTCAGCCTACACGCCCGGAAACCCTCAGATTCAAGCCAC 770

771 ACAAGATGGAGCATCCCGGTCGCCCCCGTCGTCCTGGCCGTACCAGCCGTATCAACCCTTCATGGGCGCT 840

841 CCCATGCCCCCTCCCGCCATGCCACCTCAGACGACGGCAGCGGCGACTATCGAGCGGATTTCTCCCGAGA 910

911 TGGCGGGCCGCCTCCCGACAACCATGCCCGAGGTCATCTCCCAGAGGTTTCCCGGCGACATTCCGCTCCG 980

981 CTTCCCAGCCGATTCGCAGTTCTCGTACCCGGACCTGCGGTTTTCCGACCCGAGACTCTCGGACCCCAGA 1050

1051 CTTCTGTACCCCGCAGCTGGAGCGACCGGGTTCACCGCCTACAGCAGTGGTCCCACCACCACCATGTCCA 1120

1121 TGCTCCCCACTAGTCTCACCAGCCCCCGGTACCTCCCCATGTCCCCCCCGGGGTTCCCCTCTCTCACAAG 1190

1191 TGCCCCGGGCGGATTCGTCACCAGCCCAAACTCCCCTCCCCGGCAGCTTGGGAACTACTCCCCTGGCTCC 1260

1261 CCTCCCTACGGCATCTACCACCACCTCTACGGTGGGTCGTACCAATACCCCATTCTCCCGGGGGGCAGCG 1330

1331 GGACGCCGCAGGGGCCAAGGGAACCCAGCATTCTCGCTGCCACGTCCAACTCTCAGACTCAAAAACCGCA 1400

1401 GTTGTTGCCAGCACAGGAAAAGGAAGCGAGCAGCCAACCTGGCAACGGTAACCATGGAAACAGACAGCAA 1470

1471 GATGAGCAAATGGACACCCAGCCAGGGGGGTCACCAGAAAAGCGAGAGACAGTCTGGCGCCCGTACTAAT 1540

1541 TCTGTCATCGCACGTCCAAAATTGAATTCTCTGACCAAATGATTCCATAAAGCCACGTGGCGCCAAGAGG 1610

1611 GAATTCAAACTTCCTTTTTTTATTGTGCAAAAACCATTTGAGAGTATTATGCCATTGGCAAAACTGTATA 1680

1681 GAGCTACTGTATTTATAAAGGCACAAGATTGGTGGACAGTATAGCACTCGTATCAATGACAAGTACAAGG 1750

1751 GTGTCATTGAAGGCAAACTGTCTTTAACGTTAGACTTTACAAAAGAGGCTAGGATCCTCTCAACTGTCAA 1820

1821 TTTGTCAGTCTGTCGTGTACAGTGTATCAAAATAATTATAGCCTCTACGCCGTGATGATTTCGAACAAGA 1890

1891 TACCAATCAGAAATTTGGAGGCAGAGAATTTATCGTGTTGTCGTTTTCAATCATCAAATGATTTGTCGAA 1960

1961 GACCAAACTGATGTGTGTATGCGTGATTTCTAGACAAAATAAGTAGATGGGTGTAGGGAGCAAGTATAGG 2030

2031 TGCATAGAAGGTTTCATCATTGTCACTTATATTTAAGTCATCACTGTCATTAGTAATTATACAAAATTAA 2100

2101 TGCACAATTGAAGTTTAGCGTCACTAATTACTCAGTAATTGATGAGATTTGGAACTGACATTTTGGGGTT 2170

2171 ACATCTTTCACTACTCCTCGCAATTTTAACATATTAAAATCTTATCTATTTCATGTAACAAGATGATTAA 2240

2241 GTGTCCATCCCATAGGAACTCATTGGAAAAACACTGATCAAAACAATGAAGTTGTTGCAGAGGGAGTGGA 2310

2311 CTTTAGACAGTTTAGAGTAAGATTGTTTGTCTTGTCTTTTTAAGATCAAAGTTTGTTTTCTAACCAATAG 2380

2381 AACAGTCTTTATTTTAACTGCGATAATTTTCTTTATCAGTTGAAATGTTTACAGATCCAGACGGTGTGTG 2450

2451 GTTTCAATGGGTACCGTGTGGTAGCTACTTTACAAATGCAGTAAACAAATCAATGTTTTGAATGTAGACA 2520

2521 TGCTAAGTTAAGTCAATGACCTAATAAAAAAACTGGTAAGAGCTGAGATCAAATGCTGTGATATAAAAAT 2590

2591 ATAGAAAACAAACACAAAATAGACAGACAAGTATTGTACATCATAAGCTTGTTTTAACGACATACATAAA 2660

2661 TATGAACATCTATCAGTAAGATTTTAAATTCTTAGAAACCCACTAGATTTAGGTGTTATTGCAGTGTTGT 2730

2731 ATTGGTTTCTTTGCTGTATCACTTGGTTATACTAATACCATATACTAAAAATGACTGTCTTTTAAAATGT 2800

2801 GTCTGTATTTTCTTTACTTATTATTAAGCATACATTAAAGCTATTGGTGTGTGCGTGTGTTAAGGAAGAG 2870

2871 CACATTCCACTGTTGCATTACCACTTCCTTCATACCAACCTTACCACCCCTATCTCTGTAAATACTGTGG 2940

2941 TCCACTCACCTTATATTCAGACTGAACCAAACTATCTAGAGGGGGTTCATGTTGAACAAATATTATGTGG 3010

3011 CAACGCATCAATGCAATGGGTGAATGTGTCAGATGGTCCGTTCTGTTTTTGTGCAATCTTTCCTTATTCC 3080

3081 TTTGACTTATATTCCAGATGTCTTAGTACTTAAGAAACCACATCACAGTATGCTCAAGATGCAGGATTCC 3150

3151 CTACTGTCTTTTCTACATAGGAGACCAACTTGCTGTACCAACAATATCTTGGGTGACATAGAGAGTTAAA 3220

3221 ACTGCTGTATAGTGCACAAAAACCTTTAGGAAACCTGTATGTTAAATCGATACAGACACTTGTACCGTTA 3290

3291 CGGTGGTGTACTTTCATATCTTATATGTTTCATGCTAGACAGAGAGACAAATAAAGCTTTTAAGATATTT 3360

3361 TGTTTTCGTTTGCAGTAGAGTCAGAATTGTTTGACCTTCTCAGAATAAAGCAAAT 3415

>Hr-Runx1 gene [Helro1/scaffold_31:68979-72815](http://genome.jgi-psf.org/cgi-bin/browserLoad?db=Helro1&position=scaffold_31:68979-72815)

68779 GTTTGGGTTTAAATGTTATTTTAATATTTTTATTGCAACAACAACAACAACAACTTCAACAACTACAATA 68848

68849 ACAACAACGACGACAACAACGACGACAACAACAACAACACGACAACTACAACAACACTATCATCGTCATC 68918

68919 GTCGTCACCGTCATCACCAACAACCAAAGCACAACAACATTATCGCAACCTTCATAAACAATGACAATGA 68988

68989 AATACAATCACATCAACAACAACAACATCAACAACAACATCAACAACAACAACGTCAACGACAACATCAA 69058

69059 CAACAACAACATCAACAACAACAACATCAACAATGTATGGATAGCCACAGGTTGTCCCCACATTTTTTGC 69128

69129 ACATCCCTACCAAAACATTGGCGGGCGAATAAATCTCTGCCTAACATGTTTAGGTATATATATGAGAATG 69198

69199 CCTAATTCAGATCAATTAATTGATTGATTGATTGATTGATTGATTGATTGATTGATTGATTGATTGATTG 69268

69269 ATTGATTGATTGATTGATTGATTGATTGATTGATTGATTGATTGAATGGTTGATTGAAATGAATGAATGA 69338

69339 ATGATTGATTAATAATTAATCATTCAATTAATCATTAATTGATAGTGAAATTTGTAATTATTATATGTAT 69408

69409 TGAAAAATTTCTTAATAAAAATTTATCGTGAAATAACTTTCGCCACACGCTCGCAACAAACCAACAACAA 69478

69479 ACATACAGACCCACCCACACACACAGACGCACAGACACACACAAACACATCCCAATCAGACTCTACGACA 69548

69549 CTTCCGGATTGATTAGGGACGGTACGAGAGTGAAGTTATTCGCCGGGAACGAGGATAACTCCATTGCGAA 69618

69619 TCTCCGGAACAACGAATCAACCATCGTAGCCAACAGCGTTGTCTTCAATGATCTAAGATTCATTGGAAGG 69688

69689 AGCGGAAGAGGTGAGGCCTTTTTCCTTAGCATGGCGTCATAATTATAGTCATGCAGTTATCCTTAAAATC 69758

69759 AGGGATTTTACTGTATCGGCGATAGTTTTTCAGTGGTGGGTTTGAAATCGGTTTGGTGATGATAATCGTC 69828

69829 GGTATTTTACACACGGAAAAAAATTGATACTGTCTAATGTTGTTAATGAATGATACTTTCAATCTTCAAA 69898

69899 CAACTTACCGTTACGCAATCATTGGGGATTGTGAGTAATAGTTAGACATACGCGTAGCACTGCTTGAAAG 69968

69969 TACAGAGGTATTCGTGTTAATCGATCTACCGACAATGTCTTAAACGCGAGTATCTGTAACGATAGCGGTA 70038

70039 TTTGAATCGGTATCGGTAATTGAATCAGTATCTGTGTTTGAATCGGTATCTGTGTATGAATAGGTATCTG 70108

70109 TGTATGAATCGGTATCGGTGTTTGAATCGGTATCGGTGTTTGAATAGGTATCGGTATTTGAATCGGTATC 70178

70179 GGTATTTGAATCGGTATTTGAATCGGTATTTGAATCTTTATTGGTCTCGGCAATATGTTTTAAAGGCAGT 70248

70249 ATTGATATCGGCGGTACAATTTTCGAGTTTATTTCCTAGCTTGTAATTTTAATATTACTGTTGTTGTTAT 70318

70319 TGGACATTTTTTGTGTTTAAATACCATGTTATAAATAGCCTACATACAAATACACACACACGTACACACA 70388

70389 TTCACACACACACATCCACACACACACATCCACACACACACATTCACATCCACACATACCGAATGGTAAT 70458

70459 ACAGTACATATATACCCCCTTTAATTTCTTTCCTAATAATCTTCGTAGTTCTTGGATGGTTCCCAGTGGG 70528

70529 ATTACCATAAGCAATTATGGATTCGTGCATACATACAAACATACAAACATACATACAAACATACATACAT 70598

70599 ACATACATACATACATATATACATACATACATACATACATATATACATACATACATACATACATACATAC 70668

70669 ATACATACATACATACATACATACATACATACATACATACATACATACATACATACATATATACATACAT 70738

70739 ACATACATACATACATACATACATACATACATACATACATACATACATACATACATACATACATACATTT 70808

70809 ATTACGAAGTAATTTTAAACCTACGCCAACTTATCACCGACATAATTATTTTTAATTCCCTTGATACCCA 70878

70879 ATGACGTCATCGTCGTCGTCAACATCATCATCATCACAACCATCATCATCACCATCCTCGTCATCATCAT 70948

70949 CATCACAACTATCATTATCATCACAACCTTCATCACAACCATCATCATCATCACGACCATCATCATCATC 71018

71019 ATCATGACCATCATAACCATCATCATCATCACCATAACCATCCCAATCACCATAACCATCATCATCACCA 71088

71089 CAATCATCATCACGACTATCATCATCATCACAATCATCATCATCACCATCATCATCATCATAACCATCAC 71158

71159 AACCACCATCATCATCACCAGGAAAGTTTTTCTTAATCACCATCGTCATAATGAGTGAGCCAATGATTGT 71228

71229 GGCAACCTACTCACACGCCATCAAAGTTACAGTCGATGGACCAAGAGAGCCGAGATGCAAAAGTGACGAT 71298

71299 GATGATGACGATGATGATGATGTTGTTGATGATGATGACGATGTTGTTGTTGTTGTTGATGATGACAATG 71368

71369 TTGTTGTTGTTGATGATGATGATGATGACGATGTTGTTGTTGTCGTTGCTGATGAAGACGTTAACGTTAC 71438

71439 TGTGTTATTATTATTATTATTATTATTGCCGTCGTTGTTGTTGTTGTTCCAGAATTCGACCAGCAGTCCC 71508

71509 CGCCCTTACTTAGCATCTCCTCGCATTGCAAGCAGTTTCGAGATGGCGACTGCAACAACAACAACAACAA 71578

71579 CAACAACAACAACAACAACAATAATAATAATAACAATAATAATAATATGGATCATAATAATAATAACAGC 71648

71649 AGCAACAACAACAACAATAGCAATATTAACAAATCGAACGAGGTACGCAGAAATGTTTGCGCTGTAAAAT 71718

71719 TGGAATTTGACGGTCTCATTAGACAGGATTATAACTTTCACAACAACAACATCAACAACAACAACAGTAA 71788

71789 CAACAATAATAATAATAACAACAACAATATCGTCAGCCACTACAACACTAGCCGAAACAACGACATCAAT 71858

71859 TACAACATCATTGACGACCGCAAAGACATCAGTTACTCCAACATCAACAACTACTACAACAACAACTGCA 71928

71929 ACATCAACTACAGCTACTATTACAACTACAACAACAGCAGCAACAACAACAGCTACAACAACTATTACAA 71998

71999 CAACAACAACAATAACAATAAACATATCGAAGCAATTAACCGACATAGTTTTGCATATTATTTGAGTTTA 72068

72069 ACGCGCGCATGCGCGCTAAAATCCTCCGCGTTGCCGTTCTATTTGGAACTTAATAATTTTAAAGAAATGG 72138

72139 CAACACTGTCATCGTCTACGTCATCATCGTCTACGTCATCATCGTCGTTTCTTTTGGCAAAAATAATGTC 72208

72209 TATAACAACAACAACATCGACATCATCATCATCAACATCAGCAAAATCATTATCATCATCATCAACAACA 72278

72279 ATATCATTATCATCATCATCATCAACAACAACAACAATAACATCATCATCATCATCATCATCGACAACAT 72348

72349 CATCATCAGCAACAGCAGAAGCAGGAGTAGTAGCAGCAGCAACTTCAAAAATATTACCGTCATCCACAAC 72418

72419 ATTATTAAAATCCTCAACAACTCAGACATCAATGTTATCAATATCTAGCATAATATCTGAAAGGAAGATA 72488

72489 ACACCAACAACAACATCATCGTCATCAACAACAACATCATCATCATCATCATCAACAACAACATCGTCAA 72558

72559 CAACAACAGCAAATTACATCAACAGCAGAAACTTAATTATTGTGAATAGGCCAAACCATTCCAATGCAAA 72628

72629 CCTCAACAAGAACATCAACATCAACAACAACATCAACAACAACATCAGCAACAGCATCAACAACAACATC 72698

72699 AACAACACTATCAACAAAAACACAGCAACAACAAACAACTTTGTCGGTGACGTCAGAATAAATTTGAAAA 72768

72769 TTAAAAGCGAACCTAGAGTTAAATTTTCACTCTGGAGACCATACTAAAGAATACAAATCGTTTTGTCATT 72838

72839 TCATTTTCGGTCAAAAAACAAATCGAGTTATATTTCGAAATTGACAATTTTAATAATAATAAAATACACA 72908

72909 CACACGAACACACACACACATTATTTGAGGTTTAAATCTTCGATGAATTCTTTATAGCTGTCAATCAAGA 72978

72979 GACTGTAAAAAGAGAACGGTGAAGATGGCGTTTTATC 73015

>Hr-Runx1 CDS

1 ATGACAATGAAATACAATCACATCAACAACAACAACATCAACAACAACATCAACAACAACAACGTCAACG 70

71 ACAACATCAACAACAACAACATCAACAACAACAACATCAACAATGTATGGATAGCCACAGGTTGTCCCCA 140

141 CATTTTTTGCACATCCCTACCAAAACATTGGCGGGCGAATAAATCTCTGCCTAACATGTTTAGACTCTAC 210

211 GACACTTCCGGATTGATTAGGGACGGTACGAGAGTGAAGTTATTCGCCGGGAACGAGGATAACTCCATTG 280

281 CGAATCTCCGGAACAACGAATCAACCATCGTAGCCAACAGCGTTGTCTTCAATGATCTAAGATTCATTGG 350

351 AAGGAGCGGAAGAGGAAAGTTTTTCTTAATCACCATCGTCATAATGAGTGAGCCAATGATTGTGGCAACC 420

421 TACTCACACGCCATCAAAGTTACAGTCGATGGACCAAGAGAGCCGAGATGCAAAAAATTCGACCAGCAGT 490

491 CCCCGCCCTTACTTAGCATCTCCTCGCATTGCAAGCAGTTTCGAGATGGCGACTGCAACAACAACAACAA 560

561 CAACAACAACAACAACAACAACAATAATAATAATAACAATAATAATAATATGGATCATAATAATAATAAC 630

631 AGCAGCAACAACAACAACAATAGCAATATTAACAAATCGAACGAGGTACGCAGAAATGTTTGCGCTGTAA 700

701 AATTGGAATTTGACGGTCTCATTAGACAGGATTATAACTTTCACAACAACAACATCAACAACAACAACAG 770

771 TAACAACAATAATAATAATAACAACAACAATATCGTCAGCCACTACAACACTAGCCGAAACAACGACATC 840

841 AATTACAACATCATTGACGACCGCAAAGACATCAGTTACTCCAACATCAACAACTACTACAACAACAACT 910

911 GCAACATCAACTACAGCTACTATTACAACTACAACAACAGCAGCAACAACAACAGCTACAACAACTATTA 980

981 CAACAACAACAACAATAACAATAAACATATCGAAGCAATTAACCGACATACAAATTACATCAACAGCAGA 1050

1051 AACTTAATTATTGTGAATAGGCCAAACCATTCCAATGCAAACCTCAACAAGAACATCAACATCAACAACA 1120

1121 ACATCAACAACAACATCAGCAACAGCATCAACAACAACATCAACAACACTATCAACAAAAACACAGCAAC 1190

1191 AACAAACAACTTTGTCGGTGACGTCAGAATAAATTTGAAAATTAAAAGCGAACCTAGAGTTAAATTTTCA 1260

1261 CTCTGGAGACCATACTAA 1278

>Hr-Runx2 gene [Helro1/scaffold_1:10102664-10109794](http://genome.jgi-psf.org/cgi-bin/browserLoad?db=Helro1&position=scaffold_1:10102664-10109794)

10109994 ATAAACTAAAAGGCACATTCAACGAACATAAAATATATATATATATAAACATGCATACGTACAATGTGGG 10109925

10109924 ATTATTTAATCAACAATAACATATGTACGTGACAGTTCCTATACTAATATGAGTTTATATGCGAGTGTGC 10109855

10109854 GCATGTATTCTTGTGTATATATACCGTTTACTTGTGATCTTGGGTTTCAGTTCAGGGAGCGATAGACAAG 10109785

10109784 ATTGGGAGGTTGTGTGTTACTGGATAGGTCTGATTGTGTTTGTTTGTTTGTTCTAATTTTAAAACTTCAT 10109715

10109714 TCATCATTTTATTGGTTGTAAATTAGATTAAAACTCAGTGAGATAGTAACATAATAAACATATTATTAAT 10109645

10109644 AGAATTTATAATAATAATAATAATAATACAAATAATAGTAGCATTGTTGACGACAATAATAAGAACGAGA 10109575

10109574 CGGCGATATTAAATATTCGTTATGTACGTTGCACTTCAAGGCATTTTTTATTGAATCTGTTGATGGTGCT 10109505

10109504 GGCACATTGAATAAATATTTAAACTTCTCATCGATCCATTCATTCATCATTTTTCACATTATTATCATCA 10109435

10109434 TCATCTTCTTCCTACCATCGTCACCAACACCACCATCTCGTAATCATCATTATCACCCTTTTCCATTATC 10109365

10109364 ATCATCATCATCAAGATGAACGACATTTTCTTAGAAGAAGCCACTTTCAATCAAATTTTGTGCGATTCGT 10109295

10109294 CAGACGACGATCATCATCATCATCTCCACTATCATCACCATCATCATTATCTTCCTCCTCCTACCCATCG 10109225

10109224 TCATCATCACCAACATCGAGAAGATTCACCATCATCATCGTCGTTATCATCGTCACCGTCATCATCGTAC 10109155

10109154 AATCATCACCACCATCATATGCAAGAGTTGCTTAATGATGATAGCAATCACTACAAACGTCATCATCATC 10109085

10109084 AACATCATCGTTTGATGAGAACCGGAAGTCCCAATGTTATCTGCTCAGAACTTCCGGAACACTGGAGGTC 10109015

10109014 CAACAAACAACTGCGCTTCAAGTTCAAGGTGGTTGCAATGATGATGATGGTGGTGATGATGATAGTGATA 10108945

10108944 ATGATGGCGAGATGATGGCGATGGTGATGATGGTGGTGGTATTTTTAATCATACAGGCTGTCAAAGAAGT 10108875

10108874 TAACCTATGTACTTGAAATGCTTATTAATTATGTTTACATTTGTAAGATGTTGGCGGTTACTTTGATGAT 10108805

10108804 AATGATGATGATGATGATGATGATGAGGGTGATGATAACATGTAAAAAGCTTTAGTGTTAATATCAAGTT 10108735

10108734 TAAAAGTAATCGTCATTATCAATCAAAAAAGTTTCAACATTCACCACAATCCAAACCGAATTTGAAGGTC 10108665

10108664 GTAATACTAGGCAAGGTCAAAGATGGAACCATGGTAACACTTTCGGCTGCCAATGAGAACAATGAAAAGG 10108595

10108594 CGGAGCTAAGAAATTATGCGGCCTATGTGACCAATCAGGTGGCAGTATTCAATGACCTGAGGTTCATTGG 10108525

10108524 CAGAAGCGGCAGAGGTTGGATGATGATGATGGTGATTATGATGATGTTCTAGATGATTATGGTGATGACG 10108455

10108454 ATGAAGATGATGATGTTTTAGATGATAATGATGATGACGATTATAACGACGATGAAGATGAAGATGAAGA 10108385

10108384 CGATGACGCAGATGATGACATTAAAAATAGTTAGAAACGAGTAAGAGTGAATATGGCAAATGATGATATT 10108315

10108314 AATTGGCTGGTCAAGTTATGAGTGACTGAACGATTAAGATGATGATGTAGCATTTATGAACCTATTCTGA 10108245

10108244 TCATTACGAAATGATGACAAATATTATAACGATGACTATGCAACGGTTATAGAGTTAACGTTACTAACGG 10108175

10108174 TATCAATGACATATGTGGTGATGGTATCTGAATACACTCCTATACACTCCTATACACTCGAGAATGAATT 10108105

10108104 GGAAAACAGTATACTATTCTAACTTTAATAAAATAAATAATGACGACAAAAATTTGGTTAGAAATATTGA 10108035

10108034 GAAATGTAATAAAAAAACTATCCAACGCTAGAAATTCGGTAAATTTTATAAATTATTGCATTAATTAAGG 10107965

10107964 TTTTTTACTAGCATTCACTAATATATATTTATATATTATGTATTATATATATTTATATATATGTATACAC 10107895

10107894 ACGCGCGCGTGCACACACACGTGTATATATGTGTGTGTGTGTATGTGTTTGTGTATGTATGTGTGTTTGT 10107825

10107824 ATGTATGTGTGTGAGCGTGTGTGTCTATGTACATGTGGTAAAAAATAATTACAGTGATGTCTTTGATAGC 10107755

10107754 AGATATATATATATTTCAACGAAATCGGCCATTATTGCATTGTTTGCTCTTAGGGAAATTTTTCACAATC 10107685

10107684 TTCCTCAACTTCCTCATCGTCGTCATCATAACCACTATAATATTTATTGCCATAATGATAATAATAATAA 10107615

10107614 TAATAATCATCATCATCATTATTATCACTATAATCGTCATACCACAGTTATTACCGTCGCCATCATCATC 10107545

10107544 ATCAGCAACAACATTATCAACAACAACATTATCAACAACATCATCATCATCAACAACAACAACATCATCA 10107475

10107474 TCATCATTAACATATTCTTCATCGTCATCAACATCATCATCGGTGGTTTTAAAGATTTGTAGTGGTCTCA 10107405

10107404 AAGTCCATATGTTGCGTAACAGTTCTTGTTGATGTTGTTTGTTGACATTTTCAATCTTGTTCATTATTAT 10107335

10107334 TATTATTATTATTGTTATTATTTATTATTATTATTATTATTATTATTGATATTATCTTTTTATCTTTGTT 10107265

10107264 TGTCATTCATTTAATATTTTTAAACTTTGTTAAAATATTTTAAATAACATTCACTCTGTCATGTGTATGT 10107195

10107194 TGTTGGTACTGATGTTTAATGTATGTTGTTGTTTAGGTTTAAGTTGATTGTGTTGTTGTTTAAGTTATTG 10107125

10107124 TTTATGTTATTGTTAATGTTGTTGTTGTCTATGTTGATGTTGTCTATGTTGATGTTTTTTTATGTTGTTG 10107055

10107054 TTTAGGCTGTTGTTTATGTTGTCGTTTATGTTGTTGTTGTTGTTGTTACAAATGTTAATACTTCTGTTGC 10106985

10106984 TCGCTATTTATAGCATTGTCGTTACGCTTGCTTGTTGTCAATGTTGTTTAATAGGATCTTTCTTAGAAAT 10106915

10106914 TTGTTTTTTCATTTCAACGTTATTATTAATTCGTGCTGTTATTGTTATTGTTGTTGTTGTTATTATTGTT 10106845

10106844 ATTGTTGTTATTTTATGTTATTGTTAGTTGCCACACCTCGTTATTTTAATGTTAAAATATTAGTCACAGG 10106775

10106774 CGCGGTAAGCACAATAACAATAGTAACAATAATAACTCTAATAACATCAATAACAATTATTAATTATAGA 10106705

10106704 ATATAAATAAATGAATAATAATAACTCTAATAACAATTATTAATTATAGAATATAAATAAATGAATATTA 10106635

10106634 TTAATAATAATAATGTGATTTACTTAATATGGTTAAATATGTCTATAGGTACGTGTGTGTATCTGTGTAT 10106565

10106564 ATGTGTTTGTCTGTAAGCGCGCGCGTGTGTATGATTTCATTTATGAAAAAATTAAAATGTTGAAAAATAT 10106495

10106494 ATGGGTTACTTTTTGACCACTGTTGAATTAAAAATATCAAGAAAAAAAGTAGATTGAATAAAAAACATTA 10106425

10106424 AAGTAGATTAAAACCAATGGTAATATAAAGAAGAAAAAATATATTAACTCTTTATTATTATTTATTATTA 10106355

10106354 TTGTTATTAATTTTATTATTATTATTATTAGAGTGTATTTCGTTCTAGAACAATTTTTATACTCTTGATA 10106285

10106284 CTTTTGGTCTGAATAGGTGACCGATAAAGAAACTTTTTTTTCTATCTACCTGTCTAAAGCTCTCTTTTCC 10106215

10106214 TCTTCCTCTCTCTCTCTCTCCCTCTCTCTCTCTCACCCTTTCTATCACACTTTCTCTCTCTTTTTCTCCA 10106145

10106144 TAATACAATATTGATAATAAGTCCATTATTTTTTTTAAATTTAAGTGGAGCATTTTTGGTTAATTGCAAC 10106075

10106074 ATTAATAATTATTATTATTATTATTTATAGCAATAATAATTATTATTGCTATTGTTATTATTATTATTAC 10106005

10106004 TATTATTATTATTGTAATAATAGGAGTTTATTGATGCTAAATATAGCATCTAGGCTGGCTTCCATTCATT 10105935

10105934 GATTGTCTAGTTCTAATTGGGCTAATTAAGGGCATGCTATTAATGTCCTTGAGTTCATAAACGGTTTTAA 10105865

10105864 ATATTATTATTATTATTGATAATACTGTTATTATTAATGACATTATTATTATTATTTTATAAATTGTTAT 10105795

10105794 TTTTTTACGTATTTTATTACTTTTTAAAAATGGTTTTAAATTTGAATATTTTTAAAATGTTTTTTATTAC 10105725

10105724 ACTAGTTAGCGTCAAAAAAGAACCATGCATGTATTAATTAGTATATTTACTGTTACATCAGCTGCTAATA 10105655

10105654 TTGTATCACTTTAACGTTGAAATAATTTTTAAAAGCCATTCTATTATTATTATTATTTTTTGTTATAATA 10105585

10105584 ATAATAATAATAATTATTATTATTATTATTACAACAACAACAACAATACAAATAACAGTTATTATCGTTG 10105515

10105514 TTGTTGATGATGTTGTTAACAACGACATTAATTTTAATGAACAAGTTTTTAGTTCATCGGTATTTGAACG 10105445

10105444 TTGTACCCCATGTTCCGAGTTTTTTATTGAGAAAAAGTGAAATAGAATGAAAATAACAACATTTACTACT 10105375

10105374 ACTACTACTACTACTACTACTACTACTACTACTACTACTACTACTACTACTACAGCTACTACTACTACTA 10105305

10105304 CTACTACAGCTGCTACTACTACTACGGCCACTACTACTACTGCTGCTGCTACTACTACTAATACTGCTAT 10105235

10105234 TGCTACTACTTCAATTCCAACTACTACTACTTTTTACTTCTACTACTACTACCAATACAACAACAACAAC 10105165

10105164 AACTACTACTGCTTCTAATACTACTACTATTACTACAACTACTACAAATGTTACATTAATGCCCTCGGTA 10105095

10105094 TTTCGATAACTCGTTTAAAATAATAATAACCATAATAATAATAATAATAATAAATAATCATAATAATAAT 10105025

10105024 AATCATAATGATAATAGTAACAGGAACAATAACATGATATGAGTGGTATTAACAAAAATGAGAGTTTAGC 10104955

10104954 AGTACTCTTAACATTAGTCACAATTTGCTGCTGTTCGTCTGTGTTTGTGTTGCGGTAACACGTGCGTTTA 10104885

10104884 CGCATATGTGAGTGTGTATGTGTGTGTATGTATGTGTGTGTATGCGTGTTGTTGTGTGTGAATGTGTATG 10104815

10104814 TGTGTGTGTGTGTGTGTATGCGTGTTGTGTGTGAATGTGCTGATAAGAGAACACAGAGGTACATGGTGCT 10104745

10104744 GATGAGTGTATCGTGCTGTTGAGTGAACGAGCATTCTTTAATGTTAAGTCATCATGTTGTGTTGTTGTTT 10104675

10104674 TTTTGTGCTGTTGTATGTTTGTGGTTGTTGTTGTTGTTGATGTTGTAGTTGCGTTATTGTTGTTGTTGTC 10104605

10104604 GTTGTTGTTATTATTTCTGCCTGTCTGTTACGGATTATTATCTCTTGATGTTTTTCTTGTTTACGCTTTT 10104535

10104534 GTTGATGTTGTCGGTGAGTAATTAATTGGTTGAGAAACAACTGCTGTTGCGTTTGTCGTTGTTCAGTCTT 10104465

10104464 GTTCACCTGTCGAAATAGCTAATGCTTACTATTTGTGATGTTATTGTTGTGGTTTTGTTGTTACTGATGA 10104395

10104394 CGTTAATATGTTATGATGTTGATATTTTTAATGATTATGTTGATGCAATTGATGCGAGTGAGTATCTATC 10104325

10104324 GTTGTCGTTCGCCGCTGTTCTCAATCAGCTCTGATTTCTTCTGATGCATCTTTTCAGAATTTGTATTCAT 10104255

10104254 TTATCTGTTCACTGTAGTTCTTTGTTGTTGTTGCCGTTCATTTAAAGTTAGTTTAAATGGCTGTTGTTTT 10104185

10104184 CTGCAGCAATGTTTTTTCCCAATAGTTAGTATAATGATGACGAAGATGACGACGATTACGATGTAAATAA 10104115

10104114 GAAGGTTTTAAGTAACGTGAATGAACTTAAGAACAAACATTAATAAATGAATGAGCGAATGTAAAAATAA 10104045

10104044 ATGGATAATGTATGAATGTATAATGAGAAAAATGCGGGAAATTTTTATATAAACAATGGGGGTAGAGTTT 10103975

10103974 TTTCTCTTTTACTCTCTTCATATCTCTCTTTCTCTTTCTTTTTCTCTTTTTCTGTCTCTCTTTCTTTCTG 10103905

10103904 TTTCTCTCTCTCTCTCTCTCTCTCTCTCTCTCTTTTATATTAACTCTTGTTATTGCATTTTTCAATCTTT 10103835

10103834 CCCTGCTACTCCCAACTTTCAGTTTAGTTGATTAGGTAGATTTGTGTAGCATTTACATTCTGGTATATAA 10103765

10103764 GATTTGTTTGTATCTGTTTCTAGATGTTTGTATATGTTTATGTGTATTTTTTTATGTCTGTATAAGTTTG 10103695

10103694 TATATGTGTTTGTAGATATTTATATATGTTTGTATGTGTTTGGTATGTTTTGTATATGATAGTTTTTATA 10103625

10103624 GTGTTTTGTATATGTTTGTATATATTTTTTACGGGCTTGTCTTTCAAAGGGACTGAAGGGACTGTGTGAT 10103555

10103554 ATTGCGTGCTAGCATGTTTGTGTTTGTATGTGCTCACATGTGTTTGCATGTGCTTGCATAGGTGTGTGTA 10103485

10103484 CTTTCTTGATATGACTAATGTGTTTTTGCGTTCTAAATTAAATTCTTCAAATAGCTGAGGTCTTTTCAGT 10103415

10103414 AAGTTACACGCAAGTCTTTGTGTGTGTTTGTATGTGCGTGTCTGTGTGTTTGTTTGTGTGTGCGTGTTTG 10103345

10103344 TGTGTTTGTGTGTGCGTGTTTGTATGCATGTGTGTACGTGTACGTGGTTTGTTATTATGTCAATGCGTTA 10103275

10103274 CTGTGTGCTTGTCAATATGCACGTGTGAAATTCCAACGTAAAGACGCATGTTACGTTATAAATTATATGC 10103205

10103204 ATTATATTATATAATGTATAAAAAATGAATGTATGAAATGTGCAAATGTATGTGTGTTTGTGTTTTGCTG 10103135

10103134 TTTAGCCGCATTTCGTCCAAATATACACATTACGAACTTATTTATGTTTGCCAGCGTTTATTATATATAG 10103065

10103064 CTAGCCATTGTAAACAATTACTTCACGCATCTACCAATCAACCAACTGACCACCCAGCCAACCAATCAAC 10102995

10102994 CAACTGACCACCCAACTAACCACACAACCAACCAATCAACCAACCAACCACACAACTAACCACACAACCA 10102925

10102924 ACCAACCAATCAACCAACCAACCAGGCAAGCACCTCCACATAACGATCACCATCCACAGCGCGCCTGTCC 10102855

10102854 AGGTAGCCATGTACCTGAACGCTATCAAAGTGACCATCGATGGACCGCGCATGCCCAGAAGTGAAGATAA 10102785

10102784 TGATGATGATGATGATGTTGATGATGATGATTATAATGATGATAATAATGATGATGATATATATATGATA 10102715

10102714 CTGACAATATGTGCATCCACCTATCGTATTAAGATTCATTCTCGTAATTGACCGTTAGAATTTTTTTTTA 10102645

10102644 ATTTTAAATATTTCCGTTTAATTAGTTAATTGAATGAAAGATAAAATTCAATTAACATATTAAACATTAA 10102575

10102574 CATGTTAAAAATTAACATATTCAAAAATAACATGTTTAAAATTAACATATTTAAAATTAGCCTTTTTAAA 10102505

10102504 TTAACATATTCATCCTGCGACTACAGTTTCCAAAGGAAAGA 10102464

>Hr-Runx2 mRNA (partial)

1 GATAGACAAGATTGGGAGATTAAAACTCAGTGAGATAGTAACATAATAAACATATTATTAATAGAATTTA 70

71 TAATAATAATAATAATAATACAAATAATAGTAGCATTGTTGACGACAATAATAAGAACGAGACGGCGATA 140

141 TTAAATATTCGTTATGTACGTTGCACTTCAAGGCATTTTTTATTGAATCTGTTGATGGTGCTGGCACATT 210

211 GAATAAATATTTAAACTTCTCATCGATCCATTCATTCATCATTTTTCACATTATTATCATCATCATCTTC 280

281 TTCCTACCATCGTCACCAACACCACCATCTCGTAATCATCATTATCACCCTTTTCCATTATCATCATCAT 350

351 CATCAAGATGAACGACATTTTCTTAGAAGAAGCCACTTTCAATCAAATTTTGTGCGATTCGTCAGACGAC 420

421 GATCATCATCATCATCTCCACTATCATCACCATCATCATTATCTTCCTCCTCCTACCCATCGTCATCATC 490

491 ACCAACATCGAGAAGATTCACCATCATCATCGTCGTTATCATCGTCACCGTCATCATCGTACAATCATCA 560

561 CCACCATCATATGCAAGAGTTGCTTAATGATGATAGCAATCACTACAAACGTCATCATCATCAACATCAT 630

631 CGTTTGATGAGAACCGGAAGTCCCAATGTTATCTGCTCAGAACTTCCGGAACACTGGAGGTCCAACAAAC 700

701 AACTGCGCTTCAAGTTCAAGGTCGTAATACTAGGCAAGGTCAAAGATGGAACCATGGTAACACTTTCGGC 770

771 TGCCAATGAGAACAATGAAAAGGCGGAGCTAAGAAATTATGCGGCCTATGTGACCAATCAGGTGGCAGTA 840

841 TTCAATGACCTGAGGTTCATTGGCAGAAGCGGCAGAGGCAAGCACCTCCACATAACGATCACCATCCACA 910

911 GCGCGCCTGTCCAGGTAGCCATGTACCTGAACGCTATCAAAGTGACCATCGATGGACCGCGCATGCCCAG 980

981 AAGTGAAGATAATGATGATGATGATGATGTTGATGATGATGATTATAATGATGATAATAATGATGATGAT 1050

1051 ATATATATGATACTGACAATATGTGCATCCACCTATCGTATTAAGATTCATTCTCGTAATTGA 1113

>CspI-Runx gene

GGAATTCAGGGCCCCATTTTTTGATTAGGAACCCATAATTCCCAAAAATACTTTTCTTTC

GGTTTTGGGTTTTTTTTTTTTTTTTTTTTGGCCCAAAAGGAACCGGAAAGGCCCCCCCCC

CCCCCCGCACATTTCACCACCCCCTTACCCCTAATCATGGTGCCATGTACATCACTGCCC

ACTCTATGAAGAACATGTTGTATCCGTGAAATGCATTCTATAAATAACCATTGATTACAT

TGAATTTGAATATGTTCATTGTGCACCTTTTGAACGACTTTTCGATCTCGCAAATCATAT

CGTATGTTTGGTTACACAATTTGTATTCTGCTTGTACATGATTGCATGACATGTACATAT

AGGTATATCACCATTATCTATTTGCTGGTGCTGTACTGTGGATTGCATGTGGATACATGC

ACATATAATTATAACACGTAGGCCTACACACATCCCGTGTCGGCTGAATTATCCTAGAAT

TGCATAACAACAGTGTGGTTTTTTTTTTGTTTTTTTTTTTTGGGGGGGGGGTGTTCTTCA

TGGCAATTGAGGAAATTCACTCGGGTCTCGCGTTGAGTGTGCGGTTGGATCAATTGCGTC

ACAGCAGAGCGTTGATACAAGCTGAGTGCACATGCACCAGAAATGCCATTGAACAAGGCA

GCACATCTGCTGCGCCCCAATGAGTAATTAACCAACGTAAGCCACGATGTCCCATTGCCT

ATACGCCGTAGGGTTGGTGGGCATGGCCTAGTTAATTAATGGATGGATTAAATGTGTGGA

TACTAATTAAATTATACTTCATACTACTTGTACAACTACACATTATTCAATCCTAATCTT

TTGTTTTGCCCATCTTCATCTCATGGGAAGTTCATCCCCCCCACCCCCCCTCACGCGTGC

GTTCTCAATGCTATTTGACTTGCGTCATTGATTTCATTTCGATCCTTGTGGGGAGGGGGG

GTGTCACTGACCGTGCGGAGGAGGAGGAGGCGACTTCCTGTTCATGATCGGTGCTGTTTT

GATATTTCTTGGTCCTAGAAATGCCTTGAATCACTTCATTAATCACTCGAATTTCACGGA

AATGCGCGCGACCGTGAACTCAGTGAAGATACACTTTTATAGATGCCCAACTGAGTGTGA

ATCATCTGTTTACGAGCCAACAAACGAAATGTTAGACCTAACATTTCACTGTTCGCTTGA

ACGCGGTAGTAGTACTCCTACACTGAAATGTCTAATTGCATGAACATTAAACTTGACAAT

CAGGAACTTTTTTTTATTGTGTACTAAGTTTAGATTTTCTTCATTTTGTTTACGATTCGT

TGTATGTAAGTAAATGTGTTTAACGCTAAACACTCGAAGACTCCGCCCCCGCTTCCTGAT

TGGCTGAGACCGGATGAGTCATACTCAGCACCATGCCTGGCCGCATATATAAACCAAACC

ACACTCCAGTTCATTCACAAACCACAGATCATGCGTACTGTTGGCTGGCCTATATTTTGC

TAAACTCTTCTGAAGAGAGAGAAAGAGAAAGAGAGAAAAAACTTCTCTTTTTTTTTATAA

GCTTCAATATATATTTTTTTCTACGTTGTGCTACAACAACCCTCCATGCATCTTCGTTCA

GATCTGAACGGCACGTCGCCGTTGGCCGCTTCCGCCGCCACAGACTCCCCTCCTTCGTCT

CTTCAGACGTCCAAGCACCCTGGACCAGGATTCTCGGCGTCTGCCATGGACATCCTGACG

GGCGAGAGGACCCTGAGCGCCGTCCTGAGTGAGCATCCTGGTGAGCTTGTTCGGACTGGC

AGTCCGAACTTCGTGTGTTCCGTGCTGCCATCGCACTGGCGCTCCAACAAGACCCTGCCT

GTGTCCTTCAAGGTGGTTGCCCTGGGAGAGGTGAAGGATGGCACCAAGGTGACGCTGAAC

GTCGGCAATGATGAGAACTGCTGCGGAGAATTGCGCAACGCTGTCACGTACATGAAGAAC

CACGTCGCTAAGTTCAATGACCTCAGATTCGTGGGACGCAGCGGAAGAGGTGAGCCATCA

TTCCATTAAAGTTTTCATACCCGTTATTTTACGAAGTCATCAAAACTCGCTAGAAAGCCA

TGTAAGACCCATCCTTATAGCTTGCATTTTTGGCTGAGAACTCAAGAGGTTTCCTTAACA

TCCTGAACAGCACCATCCTCATTCATAGCGATGCATCATAACTCACTAAACAGATGTAAG

CAAATAATGAGCATTAATATACACAGGTATTGTGCGTGTTCTTATACGGTCAGATATATG

TGTTATAATAGTAGGCCTGTATACACATCCAATATAGTCCCTAATTTGTTTACCAACGGT

GTTATTGCTGTTATCATGCAACCTTTGATCGAAGAAAGTTCTTGAAATGCTCACGGCATT

TGCGTGCTGAGGTCAGCATTGACTAGATCCGTTATTCGGCATCTGCACTTTAATTATGCT

ATCCGTGTCTACCTTGATGGTGTGCGCCCAGGGGTGGCCCACAAATAGCAGGCTGATGTG

TGCCTCCTCCTCCTCCTAATGACCTCATGCTTTGGGTCTCCCCTCCCTGGCCTCGATCAA

AAGCTCCCAGGGGCGGCGAAAGTACTGGCTGTTGTGTGCTTTCTTCCTTGAACCCTCGAC

CCTATAGACAGATATTCAAATGAAGAAGTCACCTGTGCCCGGTGGCTGTCTTATTCTATT

GAACTCCGCTCACTTCATATAAAATGCTGCATGCAAATGTATATAGTACACGCTTTATAT

GCGAATGAGGAACAAGTGCACCTGAGGTTCGTGAAAGCATAATATGTAAATCAAGTGCAT

ATTAGCAAGAGAGCGCAGTGAGATCAGAATACTGGAACTTTAACCGCCGACCCTAACGTA

ACACATCCCGCATATTACGCGAGAAAATCCCATCCACAAACAGACCGACAGGAGACGAAC

GCATGGCATAACATTTTCATGTCTGTGTGGCTTTCCAGCAGAGGTGGCGAAGTACACGGG

CTTCATGCTAACTACTTTGGGTCAAAGCACTTGAAGCAGTCGTTGACTCACTTAATTCTC

TTATGAAGTTATGGATCAGCTCGTATTTCCAGGATTTCTTTTAATGACGTGTGTCATTTA

TCATCATCGCCGCCATTAAAGTGACATCATCAGATGTTAAGTACCGCAACTCTTTTATTG

TAATGTTAAAGAAGCCACACATGAGCAAGCCACACGCATGCGCAGATCAAAGAAACCCCC

TTTAATTAGCTTGCATTTAATTGATGCCAGTCTGGCGTGCAAGGCCAGTAAGTGCAAGCA

GAAGTCAAACGCACCTAGCTGTATGATTGAGTGGCCTCTGAGTACATTGCATTCTGTGCA

CTTTCAGGAAAGAGCTTCAACCTGACGATCTGTGTGCAGACGAACCCTCCCCAAGTGGCA

ACCTTCCAGAAAGCCATCAAAGTGACCGTCGATGGCCCACGCGAGCCCCGCAGCAAGACT

AGTAAGTACTGTCCATGTCACTCATGCCAGGAATAGTGTATACTATATATGTATACAGTA

TATATACGGTGTACAATGTAGACAAAGAGCTTTGCTTCCGCTGTGTGCCGTTCACTCCTG

CAGCGTTGGGTGCACCGTCAATGTGTTTGCCATATTTTATTGTTGTCCTTGTTGACGTGA

CCCTGTGTTTTTCAACTTCTCTTTTATTACGCGTTTCCTCTGATGACTGAAAATGGCAGC

TCCTTATACAATGCTAGCAGCATCCTCTTCGGGACATGTATTCACACTGATGCTTTTAGA

TCTGTAGCCTTGTACAACCGGACATTGTGACATGTCAAGTGCTGTAGTAGTCGGTGTTAC

ATCTCATGCAGTGTTTTCTCTTATAAGGCGATGGGTCCCTTTTGGACTGATCCTTTAGAT

CATCGCACTATGCCCCAGTCTCCCCCGCAGTGCTTCCTCCACCTGGCTAACCCACGCGTC

GGAAAAACAGCAAACGCCAGGTCTTTTCTTCCACTTGGGGTTGCTGTAACTTAAATAACA

AGTACCTGATCGCATTTGCTCGTTAATTCGCGTAGGCGATTGAAAGACATGAAAGAAAAC

CGCAGCGTCTCGTGCCAATACTGCGACAGCTGTTTCTGTGGTTACGCAACTCAAATATAT

AGCTTTTGAGTCCGAAACAAGGCGCCCCTAATCCAGGGTTAATCCTCACATGACTGCGAT

CTGTGTGACCAATTAAAGCAGGTGCTAGACGCTGCACAAAAACACCTTTATTTTCCTCAA

ACCCACTTTTTGGTAAAACCCAAAACCCGGAGACGGCATGATAATCAAAATCGGAAGTCG

AAGGCACGTGGATTATACTGTTCGAGCAGAGAGAAAAAAAAAAATTATTATTATGGGTTC

TCCCTCTTTATGTAAGATCTAGGTCACACCACGTGCTTCCATTGAAGTCTTACTTCCTTA

ACATTATATTTGATCTCCGTTTTCATTGAGGATTAAGAGCGCTTGTTTGTGTTTACTCAC

CGCCCACGATTGGGGATTCCCACTGAATGTACTATGCAGCCATGGCTTCAGTGAAAAGTC

TCACAACCACGCCAAGAATTTATTACAGCTCTCGATCGATAAGCTAAGACAGATCTCACT

CGGTGGTTACTGGTTTAGACTATAGAACATAATATACATATATAATATGCTATTATTGGT

TCATAATATGTTTTAGCTCAAGGGTTGCAAAGCTATCTGTCATGTCAGAGACTGTAATTA

TCTACACTCACTGCTTTAGCCAACGTTTTATGCGATCCTAACAACACAAGCCTCGTCATA

AAAGACATCAGCTGGGCGCAAGAATTCGAGGGGATTCCCCTTCGCATAGCTCTCTGAATA

AACCTGATGTCAAAGGCCTGAAACTGTAGAAGTTAAATAGCGGAGTTCTTTTCATGTTAT

CACCGTGAATAACCGTTTGATGTGTCAATGTATAGCGGGGGGGGGGGGGGTATGCTTATA

GAGTACATGGTTTATTTATAGGTAGGTAGGTACACGGGAATTAGTTGTTTACAAAATTAA

TTAGTACCGTTAATTTAATGTCCAGAGTTGTTGAAAGCCCAGCCCCTCCCTACGAATCTC

TGAATGGCCAATTCACGACCAATCGTCCCCTTGCGACACGGAAAGGGGAGCATGGCCCTT

CCCTCGATCGCCCGTGGCGGCTTCCCATTCGACCGCAATTGTTTATGCCCGATGGCCCCA

TCGACATCCCTCAGGTATAGGAACACGGTGCCCATCCCTTGGTTGTGATGATTGGTCTTG

CACTTTGACCTCCGCATCACACGTGACATAACAGTGCGCCACCGGAAGCAGCACCGCCAA

ATAAGGACTTAGATGGCTCAGCGTGAAATATCGCCAATCTGAGAGCTTTTATGTCTTCAA

AGGTTATAAATGTCTTGTGTGTCTTGTGTCTTGTGTGTCTTGTGTCTTGTGTGTCTTGTG

TGTCTTGTGTCTTGTGTGTCTTGTGTGTCTTGTGTCTTGTGTGTGCCGGACGAGACTTAA

CCCATTCCCCTAATTATTCTTATATTGACCAAGAATGATGTGTTGTCGTGTCAAATGGTC

ATTGCGGGCGTCGTCATTAAACTGGTTGACTTTATCATTTCATTACTTTATCATTTCATT

ACGTCGACAAATGTATTTGTAGTTTTTTCGTCGAATTAACGGGGAATGCCCTCCAATTGG

CGCTTACATCACACTCACTGAACTCACAGTGGGTTCAAGGCAGTGCATTGTGTACTATAA

AGACGAATCGTAGACCCACTGCATGCAAACTGATACAAATACAATTGAGAGCAAATGTAG

ACATGTGCATTCTTTATAACGTTATTGCTGAGGACTCCCGCTGAGATATGTTATTTGTCG

CAAGCCACTCCTCTGCACGTAAAAGAAGTAAGTTTATTATTCCTTGGGGGAGCTAGTGAT

TCACTCCGTGTCCCAAAGTTATAAACATTCCTTCGCCTTCCGATTTCACTGCATCGTGAC

GTTCTCCCCCTCGCGCACCGGAAGTCCGGTGATCGATGAGGGGGGGGGGACGCGGATCTT

CCTCTCGAGTGGACAGTATGAGTGGACAGGAAGAGATTATCACAACACCGCTGACCAGTG

ACCAGATCATCGCTCGAGGGTCTGGTCAAGTGGCGGAGGCCTTCAAAGCCTCATCACTTG

ACACGCGAAGGACTCCATCCTCAGGCTAAAAAAAAAAGCTGGACAATATGACAGAGGGCG

AATTAAATTGACCTTACATAAGGGGATCTCTATGCATTCACAATATAGGCCTACAACTTA

GATCACTTCTTGTATAATCATCGTTTGTGGCAACCGCGATCATTTAAGAGTCCCCCGTGA

TTATTTAAGGCAGGTGCGAGGTGCCTGCCTGGTACATGTGCCAATCATTAAACCCAAACA

GCTCTTGCATGCCGGGCCTCTTTGTCAATCTAAACAGCATTGCTGCTTGAGGCATCCCAT

GTTTTCGCCCGAGGCTGTCTGCTTTTCCCCGGGATGTCCCACCGTTTATAAAGTGGCGCT

AGCAGCTTCCAGAATTAATTGTCAACTAATGGCGTCTGTGCGGTTGATGATCCCACGGAA

ACAAGTGTGATTCTCGCCACTCGCGACTTAAGTGATGTCTTGGAATGTTGAATGAGCAGA

TAATGGAGTCATTTACGCCGTACTTAATACGACACAGCGCCGTAACGGCCTCGCACCGTC

GCTCAGAGCCCTCGTCAATGGCTCGCTTTGTTCGCCCACAGGATGACACTATGACCCTGG

CTGCTGACTCCCTCTGTCATTATTCCCCTGCCAGCGGGGCTCCGAGTGATCGTATGCACC

CTGTCAATGAGGCTGCAAGGTCCAATTGGTCGCTGCAATGGGCGATGTTTAAGCTTTGAC

CGCAGGCACCGACACCCAATCAGCGTGGTTACGGTGAGGGCACGAGACCCAACCACGGCT

GAGCCCCGGTCGCGGCAGGGAAGGCTGCTGAATTTTAGGGCTCCTGCTTCAGACGCCTCC

TTTCGCGAGCGATTGGCCATGAATAGGCATCGTGTAATGCAGGACAATCGTTCTCTTCAA

AGTGAAAAAAAAAAAGTGAGGTCCAGCATTGTAGTCTGACTGACCAGCAGACAGCGGGGT

ACCATGCGAGGGGATGGATTGCTTTGAAGGGGGCAGCGACAGGGCACAGCGAGTGGCCCC

TCCTTCGCCCCTCCCATCACTATACATAGAACCCTTTGCAGCAGCTTGTGAGCGATGGTG

CCCCATCGTATGCCCTTATCAGTACAATCTGATGGTCATGATTTAAAACAGCGATCTGCG

GCTGTCATTGGAGCCCATTCAGCATGCGGCCACCACGCGCTCGAGTGTGTCTCTTTACGA

TGGCTTGATGGACGCCTGTGAGGCTCACTGCAGGTGCTTCTGACCCGAGGGCAGCCAGCG

AGAGGGAGGCGTCCGTTCTATTCACTTGACAAACGACCCCTTGACTCTCCTTCTCTGTAT

GCAATGGCTAGCCAGCCATTCATCATTGAACCCTCAATGCACCATCTCAATTGATCGAAA

TTTGATATCTGCTCGCATTTAACTCCATAATGAATGTGTCCTTTGCTCTTACAGAACTTC

GGACGGATGACCGCCGCTTGCCTCCCCGCCACAACCCCCTGGAAGGCCACCGCCATTCCC

AAATGTCGTGCCGGCCCCTGGGTGACATTGGCTCCGGTCTCAGCGTCCCAGTCCCGTCCA

TATTGGAACTGGAGAAGTTGCAGAGAGCGACGCAATCGCACAACACTGAAATGGTCACGA

CCTGCGTTCCCAGCTCCATGTACAGAAGCACGCAAGGTGAGCAGGAGAAAGAGACATCGC

ACACACTTCTTATGCACGCACCTGCTATATTTGGCCTATGTTCGATTCGTGTTGATTAGA

TTTTCAGTTTATGCTTTTCTGTGGTGTTAAGTCTAGAATGTACAGTTTCGTCATTTTGTG

TTGTATACTTTGTTTCCATGTTATTATTTCCTTTGTGTTCATTTTGCGTTCGTTGTTGGA

TGTTTACAGACACAAGCGTCACTTACTCTGGAGTTCCAGGGCCCATGCCCCCCTCCGCTG

TGGGGCCCATACCCCCGCAAGGACCCCTGCACCAAACGTGTTCTGGGGACCAGTGGGGTA

GCATGGCCCCACCGAGTTACTCGACCAGCATCCACGGGGACATGACGGCCGGCCACTTCG

GCGGCTGCATGCCCCTCGGTTCGGCGTGTTCATTCAGCCACCCGACCCCTCCGTCGTCGC

ACGGCGAGATGAACACGCCCCCGATGCAGCACCAGCACCCGCAACAGCAGCAGCAGCAGC

CCCTCGGCTTGCACGACCATTCCGCACTGACCTCGCTGACCCCTCAGTCCGTGATCAGTG

ACACTTCCCACATGTCAGGTAGGCCGATGGTAGACGCGTATAGACTAACCTCCTGATTGC

ATAAGGTTGCATAAACAGTCCGACTAACCTGCCTTAAGAATGAAACTATTGACCCACTGT

TGGCGCCGCCACTCGGTGCAATTGATGAGTTACGCATCGAAAAAACCCAGTTTGTTTTAA

CACGTGTTCGCTGTCGGTAAGGCGCTGTACATGTTAATAAGCATGAGTAACAAGGTGAGA

ATTATTCCGCGAAAAGTTTAGTCAAACAGCAGGGAGCATAAAAAGCTGCATCGCCTCGGG

GTTAAGGTTGCTGCGTGGTAGCATGCTAAGCGCTGCAAAATCTTAATGATAGCTTTGACA

GACTCGCTGACTAACTCCCCATGCAGTGAGTAATCCGAGCAAGAAGTTTATCACAAGCGG

CGCTGACGAACGAGGCGGAGAAACGAGCGAGGCACACCGGAAGTGCCCGTCTTCCTGGTC

ATCTCAGGTCGGTCTTTAAGTGGATCATCTTTTAGCGTCAGTGGTCAGAGCCGAAATTCA

TCAAGCGTTATATGACGGCGTCGCTGAAACAATGCACATTGTTCAGAGCGGCTGAAGATC

AAGTTTCTTCGTTCATAAAGTAGTTCAGGCTTTGAATGGAGCTTCCGCTCGACACTCAGA

AATTACACGTTATGTGATCCAGCAACTTTTTTTCCCTTCTTTTTCAATTCTGTGCGGATG

TATTTGGGTAAATGTCTTAAGTGCATGCACTGCTGTTGTAGGATTGCCAGCCTAAGCTGT

TACGTCACTCGATGCTGCAATAGAATAACATGCATTGTATTTTTTTTTTTTTTTTTTTTT

TTTTTGCCAGGGACATAAAAAGTTAGCGAACTTGGAAGTTTGACAAGGGAAACTGCGTTG

TTTTTAAAGTTCGTTACATCGCGATAACCTATGGGCATCTATACACCAGTCATGTTGTTC

AATTGTTTTCAAAATCAACGAGCTCTTTTTTTCTTCGAAACACCGTTACCAATAATAGAA

CCAATTATTAATCCAGTATCGATGGCATGCTTACGCAACCGCATTACGCAACACCGTCGA

TTTGTTTGGGGATGCACGGGTGGCATGCGGCAGGTGCTGCCCCCCCCCCCCCCGGTGCCC

CTGGGTGCTCGGGCGCCCACGCGCCGACCGCCGGTAGCGATGCTCGCTCGCGACCTGAAA

TCGCGGTAATTACCGTTAATTGACAACAAAGAGAAGAGTTGCTTCTGCGTGAGGAGACGG

AAGGATGTGGCGAATCTTTATGCCCGCACCCCCTCAGCCACCCCCTCCTGCCTGCTGCTT

TCTTTGTGGTGCTTGTTTAATGTCGTGTCAATTTTGTCTCTTTGCACTTAATACTTATAA

CAGGCTTATATAACATAAATTGTTAACCTGAATAATATTCAAATTGGCCGGAATTTATTT

ATCGGTTTCCTTGACCTCGTTTCATGTTGTGACCTCCTGTTTTTTCACAGGACTCCTTCA

CGGAGGCAGTCCACCCCGCCACGACCAAGTGGAGAACCTGACCGAGATGGCCCTACTTGG

GGGCACGGAGCTCTGCCCTTCGCAACCCATGCGACTCCCGCACAAAATCGAATCGAACCC

GTCTGACTTGCTGCCTCTGACGCTCTCCTCTCAAATGCCCCCTCGCACCGAGGGCCCGCC

GTCGGAGCTGCCTCTTGACCGACGTCCGCTGCCATCTGACCACCACCCGCGCTCAACGCT

CGACTCTAGCCTGCCCATGGTCATCCCCCGATACTCGAATCTAAGCGCTGAGCTGCGCTT

CAGCGACCCTCCCCGCCACATGCTGCCGCCGTCACCGACCCTGTCCAACTACCCGGTGAC

CAGTGCTAACATGGCGCTGCTTGACCAGGGCCGCTCTCTCTCGCTGACCCCGAGTGGCTA

CTCGCTGCTGTCACCGACGCTGATCGGAAGTAGCCCGAGCGCCAGTTCACTGCCCTCCTA

CTTGGCCACCAGCCCGAGCGCCATGTTCTCCGGCAGCTTCCTCTATCCGTCCTACCAAAC

GACCTTCATCCGCTCGCCGACCGACACCAGTCGCTCGCTGGATCTGATGACGTCATCGGA

GGGCGGTCGTCTTCTTCCCGCCATTTCGCCTCCCCCGCCCCAGCAACTCGGCGCTGAGGA

ACAGGACAAGAAGATCCAGCAGCATATTCCAGAACAGATGATGGTACCGCGTTTGCCACA

AGAGGGCGACCCGTCGGACCCGAGCGCCGTGTGGCGGCCGTATTGACTAACACCCCGCAG

CGCCAACACTTCGAGCTTGGTTTGTGGCTGACATTTTGGGCTGAATTACCGCCGCGCCCC

GATGTCGAGGTGGCTGTAACGTAATCAGCAGATGGCAATTCTGGTAGAATACTCACCTTG

TGGCAACTCAATTGTAAATATAGATGGACACTGGTCAACGCTCTTGACCAAGGTGCGAAA

GCGATGCACTGTCAATGCAAAGGACGAAGAGAGCTCGCGATCTCAACGGGTTTCTGGAGA

TGGAGTGGAGAACCATTTGGCGAATGGTGATCACGTTGTGTTCATAATTAAGGGAATTTA

ACTTAAGATATTTATCCTTATTTTTGATCTCGGTTGAATGTTTTGTAATCGTATTTATAA

GGGAAATACTGGAACCACCTATCAGTAATGCTTATGGTAAATTATATTGCACAAATTGTA

AACTCGTGGGTAAATTGTATGTCATTCTTCTGGAACTATACTGGCATATTCGTAACATTG

CAATCTTGCAGACCATCAAAATGGCATATACCGTACGCAGCTATTCATCAAATGGTTGCC

TTGGATGACACTGCTAATCTGTCATCTAATGGTTTCCTCATTTTTGCTCTCATTTGCATA

ATGTATTCATGGCTACAATCATTCTGCTTTTCCTGGTGCTTGCGTTTTGCCCGTTTCACC

GACTAATTTCCTTTCTCTGGAAACGATGATGTAAATGGTTATTTCATCTTGCCTTCCTAA

GTATTACGTACAATCACAAATATACTTTCACAATCAAATCATTGCATTTTCTCCTCCATT

TCGTGAATTATCAAATTAATTCCCGGGCATTTGACTAGAAAATACAAAACGTTGTTCAGA

TGGGTTTTATTTTTGTCTGTTTGCTTTGACTTCTTTTCAAAAAAAAATTTTTAGATCGGA

TTTTCAATTTTATTTTCAAATTCAAATTCATTTTGTACTGTTCCCAAATTTACATTTATA

TTTTCTCTATCTCCTTGTTTTTATTTTTGCGTTATAATAAAATTTAAAACAAAACAAAAC

AAAAAACGTTATATATATATTTTTATTTTTTTTTTTTTTTTTTTTTCGATTTAAAAGCAT

TTGAATTTTGCCGTCTGGTCAATTGTTTTCACATTAGGTCTGGTACCTTCATCACTTCAT

ATCTTTATATTTCTTTTCCCTAATCTCGTAATATTTTTGGTCGGGGTTTTACTTTGTGAG

AGACTTTTGGCGCTCTTTTATTTTTATTTCTTTGGTGAAGACGAAAAAATGACGTGTGTT

TACTTGAAGGATTGAAAATGAAACATTTTCAAAATAATATACTTCTGTCTGGTGAACTTT

TGTCTTCATATTTTGTGTTCTGAAAAGTCATCGCTGTCTGCATGATGTTATTATCCATTT

CTGTGTATATTTTATCTTGATTTTGTGTATAAAATATACGAAAAAAAGACAATAATTAAA

ATATAAAAAAAACTAATAAAAGAGTATGGAGTACTTTAAACATACCTTGTGTTAATGTTT

TTTTCTTTTGTGTTGCTTGTTCAGAGCATTGAGTGCAGTATGTATAGCCTCTCTTTTATT

ACATATACTTATATAAAGAAGTCCTCCTGTTGGAGCTATATTGTACGATGATTAGAGAAA

GGATTTGAAAGTCGAAAGATAGAAAGACTGGGAATTGGAAGCAGCCGCCACGCAACGGAT

CTTCTACTCCTTCAGTTTGAAAAAGTAATAAATTTCGGGTAAAACGAGAAAGGGATCTTT

ATAATCATCAATACCCCAATTTTTAGTTTGTTAGCAGTGTCAATGTGATGCAGTAGCTTA

TACTGAAAGAAAACAGGGTTGAGCGCCGTATAGAGGTGTGTGCTACATGCATTTACTTTA

CAAAGCATTATCCAAACGTAAAAATGGACATCGATCGATAGTCTTAATTTCATCTTCTAC

AAAGCAATACAAATCGGACGCAATTTGAAATTGATTTCTATATTCTGCATTGTGTACCCG

CGAAGGTCTTTTATTCGCAATCTGAGGCCACACATTGCTATATTTATGCCTCAGCCCCAC

TCAATTGGCGATAACTTAACCGCATCACTCATAATCCACGCAAACCACCCTCTTCGCTTA

AAATAAGCTCCCAGTGTGACGCAAGAGAGTTTTTTGGCGCCAAAAGGTTCTAATTTCTGA

CCAATGATAATGCTCAAGGCCGCCGTCTGCTGTTGATAAACCCTAAATAATAGTTAACAA

GCAGGTTTTTTCGTTAAGATTTCGCAACCTTCACTGCGACACTTTCAACTTCCGGTGTTG

CACCGTGAAATGATAGCCACACTTCCAGGTTATTAGGTGCGAAATTGTCAACGAAAGTGA

ATAACATCCTTCAGAAAAACCACAGAACATAAGATATGAAACCCCCTTGCTTGTGCCTGT

ATACTTTATCAGTACGTATAAATGACACTCGTCTAACATATATCTTTGAATGAAGCAATT

CAAAAGCGCTGTTGACAATCAAAGCATTGAATGTTTTGGACTGGTTC

>CspI-Runx peptide

MHLRSDLNGTSPLAASAATDSPPSSLQTSKHPGPGFSASAMDILTGERTLSAVLSEHPGE

LVRTGSPNFVCSVLPSHWRSNKTLPVSFKVVALGEVKDGTKVTLNVGNDENCCGELRNAV

TYMKNHVAKFNDLRFVGRSGRGKSFNLTICVQTNPPQVATFQKAIKVTVDGPREPRSKTT

GLRVIVCTLSMRLQGPIGRCNGRCLSFDRRHRHPISVVTVRARDPTTAEPRSRQGRLLNF

RAPASDASFRERLAMNRHRVMQDNRSLQKLRTDDRRLPPRHNPLEGHRHSQMSCRPLGDI

GSGLSVPVPSILELEKLQRATQSHNTEMVTTCVPSSMYRSTQDTSVTYSGVPGPMPPSAV

GPIPPQGPLHQTCSGDQWGSMAPPSYSTSIHGDMTAGHFGGCMPLGSACSFSHPTPPSSH

GEMNTPPMQHQHPQQQQQQPLGLHDHSALTSLTPQSVISDTSHMSGLLHGGSPPRHDQVE

NLTEMALLGGTELCPSQPMRLPHKIESNPSDLLPLTLSSQMPPRTEGPPSELPLDRRPLP

SDHHPRSTLDSSLPMVIPRYSNLSAELRFSDPPRHMLPPSPTLSNYPVTSANMALLDQGR

SLSLTPSGYSLLSPTLIGSSPSASSLPSYLATSPSAMFSGSFLYPSYQTTFIRSPTDTSR

SLDLMTSSEGGRLLPAISPPPPQQLGAEEQDKKIQQHIPEQMMVPRLPQEGDPSDPSAVW

RPY

>LgRunx gene (exons 1 and 2) [Lotgi1/sca_31:962949-970688](http://genome.jgi-psf.org/cgi-bin/browserLoad?db=Lotgi1&position=sca_31:962949-970688)

970930 GAAAACAGTAAACAGTTAAAATCAATAATGTGAATATTCATGAGAAGGCAGTCGAGTCTCAGGTTGGCTA 970861

970860 GAGTTAAGTTGAGTCATGAATTATAGGAGTGGCTTATAACTATAACCACATACTTTGGCTCATTCACAAA 970791

970790 ACCACATCAACCAGTTCTCTAACATATCGTTGTGTGGTATAGTAATATTATTTTAACTTCATGCATTTAC 970721

970720 CTACAGATTTGAACGGTTTGACACCGTTCCCAGACGCTCATAGTGCCATGGGAGATGTGTTCCCAGGTGA 970651

970650 CAGACCATTATCTTCCATCCTGGGGGAACATCCTGGAGAATTAGTACGAACTGGTAGTCCAAATTTTGTG 970581

970580 TGTTCTGTCCTGCCATCACATTGGAGATCAAACAAGACGTTACCTGTTGCCTTCAAAGTAGTGTCATTAG 970511

970510 GAGATGTCAAAGATGGAACTAAAGTTATTATACGTGCTGGAAATGATGAGAACTTTTGTGGTGAAATTAG 970441

970440 GAATTATACAGCCTATATGAAGAACAGAGTGGCCAAATTTAATGATTTAAGATTTGTGGGTCGCAGTGGT 970371

970370 CGAGGTAAGTCAGTACAAATCTTTTTCAACTTTTCTCAAACTTTTTCGGTATTCAAACAATAAACAATTT 970301

970300 TTTTGTAAAATGTGTTTACGTATGACAATTGACCTATAATGTGCCGTAAGAGTAAATATACAGTTAGTGT 970231

970230 AACCATGACAGACATATCAAACATTATTTTTTATTTCCTAGTTATTTCTGGCTAGTTTAACATAGTGTAT 970161

970160 AACAACCTGGATGTAATAACATGTATTTTAGTCAAGTTTATTTACCACAACAATAGAGGGTTTATCTTGA 970091

970090 TATTACTGTTATAGACTTATCATACCAATATAATCAATTCTAAACTACGTTGTTAACAGTACACTATACG 970021

970020 TGGGTGTTCCGGTAGTATTTCTCTTTTAGTCATTTAAAAAAAAAAGCATTTTATGTGTACTTAGTGACAA 969951

969950 ATACGCCTTACCTAGCTTATTTTCTCCATTCAAATATAGACATTATTTTAAAGCCCACTCACTGATGTAA 969881

969880 GTGACTTCTCTCAGCCCAAATCCATCAGCCCAAATCCATCAGCCCAAATCCATCAGAAATGGAATATGTA 969811

969810 AAGTTTCTAATTCATATAAATTGATAAATGAATAACACAAGCGTGTCAATGTAAATAATAACTTAAGTGA 969741

969740 TCTAGGGAGTCAGATAAAGTGAGGGAAGAGAAGGGGAGAGTTTACTTATTTCCTGACAGGAATTTAAGTG 969671

969670 AAATGTGTAAAAAGATTACATCATCAATCCAAGACACTGATTTTCATAGTGTTAAGTGTGGTCAATAGAT 969601

969600 TATTAGTTGTTCTTGTAAAATATTATATTTCATATTATGTATATACATGTATTGAATTGTGGTCTGTAGT 969531

969530 AAATTGATCACATTGATATGATAAGTTCTTCTAATATAGATAATAATTTGTACTTGATATGCAAAGTAAT 969461

969460 ATAGCATGATTAAGATGTTTGATATGTAATTCATATCACATTTGTGGCAAATAATTATAGTGTGGTTTAT 969391

969390 GGCACTAGATTATGGGGTCTGTATAGTAAATGTGGTATATAGTAATACATGTGTTTTGAATTGTGGTCTG 969321

969320 TAGTAGTTAAGATATTCCATATGATGTGGTTTGCAGGTAGTAATTTATGATGTATATTTTACAGTGGTCA 969251

969250 ATAAGGTAATAATGTATCTGTGTATTATTTCACAGTGGTCTATAAGGTAATAATGTATCATGTATATTTC 969181

969180 ACAGTGGTCTATAAGGTAATAATGTATCTGTGTATTATTTCACAGTAGTCTATAGGTAATAATGTATGAT 969111

969110 GTATATTTCACATTGGTCAATAGGTAATAATGCATCTGTGTATTATTTCAGAGTGGTCTATAAGGTAATA 969041

969040 ATGTATCATGTATATTTCACAGTGGTCTATAAGTTAATAATGTATCTGTGTATTATTTCAGAGTAGTCTA 968971

968970 TAGGTAATAATGCTGTATATTTCACAGTGGTCAATAGGTAATAATGTATCTGTGTATTATTTCACATTGG 968901

968900 TCTCTCTAGGTTAAAATAATGAATGTTTATATTATACAGTGTTATGATTGTGGTTAGTATTTTCTGTTTT 968831

968830 TTCTGCTCTTTACATGTTTATATTATTTAATGTTATTGCCACTAGATGATCTAAAGTTTAACTAGCTAGA 968761

968760 ATTTGAAAACGTATGATGGGTGAACAGGGTTAAAAACTGACGGTGGAGATCATAGGCAAATCATATGAAA 968691

968690 ATATTGGAATCATGAGATATTTTTTCTCACAGTTCAGAAGGGTCACTTATGTAGAATCTTTGTTTAATTT 968621

968620 TACAATTTTAACTTATAACTTGAACTATCTTACTAAACCTCTTGAGCTTGTTTACACTCCCTAAATGTAT 968551

968550 CTTGAAATTTGCCTTTTAAAGTTCAAACTAATTGAATCAAGAGATTTGATTTAATAAGTGCTGCACCTTA 968481

968480 TATTCAACAATGCTATTGTTTTCACTTGACACAGAAGAATTACCATGCAACAGAACTTATCATTAAAATA 968411

968410 TCAAATAACATCTTTAAATTGAGAATGATGAAAAATGGTGGTTTCAGTGGGGATTTTAATTTTATTTGTT 968341

968340 CATGGTGTAAGATTATGGAGCTAGGAGAATAGAAAGTGTGATCGCCTTCTGGTCTTGTGGCTTAGAAAGA 968271

968270 GACATTTTAACAGAAAGAATTTAATGGTTCATGGTTTGTGTGGTCACCCACTGTGATGATAGTCACACAA 968201

968200 TGCTTTGTATTTTTCTCTTTTTTAATGTTCTTTAATATATAATATATATTGGTTTATTTAAATACTTTTT 968131

968130 TACATATTGAATTTGGTGGAGATCTTCTGAAATTGATAATTTATTCTAGACTTAGGAATCCACATATAAA 968061

968060 ACTTACCATTTCATTATATTTTGTAGAATTGTATAACCAAAGCACAAGTACTGTATATACATGACCATGT 967991

967990 TTTGATGTTCTGTTCGGAATTATTTTCAACGGATTCCATTATTGGCTATGACTTTGAATGAGTAAACTAA 967921

967920 CAGATAGTAATTTGAATTGAATGCGACACACCTACAAATTTTATTTACATAACAGTGAAATGCACAAAAA 967851

967850 ATTCTCCAGTTAAAGGAATCGATTTCTATATCCATTAATGTATCTAAAATATGTCATGAAATAAATTCAA 967781

967780 GCTCTGTAGGTAAAAGTAGCTCATACCCGCTATAGTAAAATATTGAAATCATAAAAAGCAATATGCAACA 967711

967710 AAACTATTCCTAATGTCCTCAAAAAGTATCAATGAAGATTTATAAGATTTGATCAAATATGATATTCTTC 967641

967640 ATTTTCTGAGACACATAAAAATCGAAATAATTTATTCTTTGGAAATTTAACTAGCCTTACGTCTTGTTAT 967571

967570 AGGTTAGATCTTGGCTACACATCCTTTTTATGTGGTGAATGATGCTATCTCATTGAAAACTTGATTATTT 967501

967500 TTTCAAATTAATGAGGAAATCTAAAAGCCATTATAACAACTTTTTGAATTGCTGATTTAATTTAGATAAA 967431

967430 ACTGTGCACATCTATTTCATTTTTTTTTTTGCTTGCTGCAACACCTGCAAATACGTGTTTGCCAAAAGGA 967361

967360 AATCAGTAATGATTAGAATAGATGAAGTGTTTTGGCATCAAACTGGCATACTGTGTAAATATGACAGATA 967291

967290 TTGATACATGCATCACCCGCATAGATTGTGTCTTTGAACACTACTTTTAATCTCAATTCAACACAATTTG 967221

967220 TTTGTCAGCTCAACATTTCTCACTTAAATATCCGCCAACTTGCAGTTCTCAGATAAAAAATGTCAGTAGA 967151

967150 CGAGTGAGGATTAGGTCTAAGTTTTTTCACTGGTTTTGATGTTTTATGTTTTTGTGTTAACTATATTTGA 967081

967080 AGATACAGGACTGACAAAGGTTTTATAAGATTAGCATATTGAAGGGAAGGGATAAAGTGACATCCTTCTT 967011

967010 AACAATAAAGATATCGTTTTTTTATTAGACAACTTTTGATCTAGTTCACATTAAAGCTTGTATTAAATAA 966941

966940 GGTACTGCACACTCTGTCCTTTCATAACCCATTATATATTGTTTTATAATTGCCTTTTGCACCTTAACCA 966871

966870 AATCATATGCATAATTATCTGTGCTGATTATTTCAAGCATACATCTTCTGTACATTGATTTAGGACAAAA 966801

966800 GCAGAACGTTTATAAATGCATTTTGGAAATTGCCATTAATTGCACTGAATTGCCCATTCATCTTAAATTT 966731

966730 AAATAAATTCAATCAAATTATTTCACCTTCAATTAAAGTTTAATATGAATACTAATATGCCTGAAAATTT 966661

966660 TAATTTCATGCATATAAATTTTTATAGATTTGTTGTTATTAGTCATTTACACAACAATTGTTTTTCAAGT 966591

966590 GTTACTGTCACCTCTATTTCCTTCTTACTGTCATAAAAATATGACATGTCAACAAAATCAGTGGACAAAG 966521

966520 ACACTCCACCATTTTTCTTTGACTTGATTGATTAAGGAACTTGTGTGAGGACTGGGCCATTGAAGAAAAA 966451

966450 CCCATGTGTTTAAATTGCCTCCAGTTGATGACTTTGTCTTGCATAACAATAAGATCTTTACAAAGAAAGA 966381

966380 GGTTGACAAAAAATCAGTGAAGACTAACAAAGCAGACCTTTCATACCAGATGTGCTAGAACCCTACACTT 966311

966310 TCTGTTTTATTTTCACTTCAAAGAAGAAAAGTCTTGGCTTTAGATACACAGTAAATAATTGTATTGGTCA 966241

966240 ATATTGTGGATCCACCATCAACCATCTTGAAAAAACCTCCTTAATTAAAACAAACATAGTCAATTTTAGA 966171

966170 GGTTGATGTTGAGCTAGTATGGATACTTGTTAAAATATTTATTAATATAATGAGATATAATGATTGTCCA 966101

966100 CCTGTCACTGTTTTTATATTATTTTAACTAAATATATTCACTGTGTCTGACTGAATATAGTTTTATTTTA 966031

966030 CAAAGATCTCCCGCTTTCTTGTACAATACAAACAATTGCTTGATTCCCTTTTTATGGAATTTTATCAGAT 965961

965960 TAGGATTAGGATTATAATCTAAATTCAAATTTTTTTTCTATTTTGAACACAAGATGATGAAAATGGAGTT 965891

965890 GTTTTGTCTGCATCTTAGAAACACAAAAGGCATTTCTTTAATGATTTTCATTTCTACTGCTATTTTGGTT 965821

965820 GCTTTTTGTTTGTGCAACATTTCTGTAAAAAGATATAGTTGTCTAAATAGTAACATTACATCCACACATT 965751

965750 ACCAATATTATAATGGTGTCATTTATAACTGTTTATTCGGATTTTAATAACCACCTGGACAATCTTCTAC 965681

965680 TTCTAACACTTTTTATAGAATAAAAGGAGAACTGGAGGTTGGATTTTGCTTATCCTTTAAAGAGAAGAAA 965611

965610 ACTATTGTTTTTCCTTGACCAATGCATTTCAGAGTTAATCTGAGATAGTCTCTCATCTAGTAATGGTAAA 965541

965540 AGTTTATGATTCGGTGGAAAGATTCAAAATCAGTGCAGAAAGTTAACACATGTAGTTTCCAGAATGCTTG 965471

965470 TTCTTGTTGGGTATTTGTTTTTGACATTGATTTCAAATGTCATTCATCAAGAGAAAAGATGAGATTTCAA 965401

965400 ATTTTACAATAATAATAGAATCAAGGTTAGAGATTAAAAAACACTGTATTTGTCTGAGTGTAGGTTAATA 965331

965330 ATAGTATAATTAGAGAGAAAATCAATAATATTGTAGGTGTATATAAGTATTGTGAAATATAGAACATATT 965261

965260 AACCTAATACCACACTATCATGGTATTTCAACTAGTATAGGTGGCAGATATCATCTCTAACACAATCTGT 965191

965190 TTGCAGGAATTTCGCAATCTTTCAAATTATGTACAATTGATTATTTTATATGATGAAATTTAAATGTAGG 965121

965120 TGTTAATGCTATAAATTTTTCATATATTAAAGTGAATAACAAATAGAAGTGATTTCTATGGAGTGTTTTG 965051

965050 TTTATAGCTGTCATCTAATGTCATTACATTATTATTGTGATATAATCATTAAGAATTTTGTTGATTTGAA 964981

964980 TTTATGATTTGTGGTTTCAGTTCTTGTACTAGTAATTAGTAGGTCATTCCTGTAGGTGGTGAAATCACTT 964911

964910 AGTTGACTTAGTTGTTTATTTATTGAGCTTTCCAATAGCTTGGAGGTTGTTCAGAATTTATCTAAGATAT 964841

964840 GAATATTGAAGGATTTTTTCTATCTTTTAACAAATGTTGAAGTCAAGAATCTGTTATTTATTAGAAGGTT 964771

964770 AATCTATCTAGGTTCTCACAATATCTTCAAGGATGTTTCAATGAAGCCAATTTGTATGAAAATTAGTCTT 964701

964700 TTCTTTAAGACGATAAAATGGGTGAGAAGATGTTTAAGAGAAGTTAGCATGCAGTAATTATATTTGATAC 964631

964630 ACTTGACATGAATCAATATGCTAGAAGCTGAGGGATGTGACTGCTCAGACTTTTAAGCTAATTAAGACTA 964561

964560 ATTCTTTGTGCCCTCAACATGAGGCCAAGTCCTATTAATTCGACCTTCGACATGGGTGACGACCTTTGAC 964491

964490 TCTTCAATCATAATTGTTGACAGAGGAACAAAGAAAGTAACAAGCTGATCAAGCCTGGTTGGATATTTGT 964421

964420 ACAACTGTTTAGTTTAACAGGCAGTCACTGTCTAATATTTGATATGTCAGTTTTTACATGTAGAATTAAC 964351

964350 ACTGTCTGTGAACCCCAGTTTCTAACAAATCTTTTTATTTCTTGCAAATTCATTTCTAATACTTTAAAAT 964281

964280 ATTTCAAATATCCAACTGAAAATTGAAAGAATTCATAACATAACTGTCTTTTATATTTATGTCCTTCAAA 964211

964210 TAGAAGCTATCTTATCAATTATTCTTATAAATTGAAATTTATGCTGAAATAAAGTTAAAACAAAGCTTTG 964141

964140 AAAGTGACTCTTGGCTTCATCTTTAAAATTATTAAAATTTATGTAAAATTAAGGAAAATCTGTTTCAATT 964071

964070 GTTTTAGAAATTTTGAAAGCTCTTGACTATGATTAAAAAGTATGCAGTTATAAAATTGTTTTTGTGTTAT 964001

964000 CAATACATGTCTAATGCACACTGATCTTTATCTTTTAATAAAAAAAAGAAATCCTGAACAGGTTAGATAA 963931

963930 ATAAATAATTAGATATTGTCTGAAGCTTATAAATAAATTATAAAGAGGCAAGGTGTCTTTGTGATAAAGC 963861

963860 CTAGGTTTGATTGTATTTAAAGGTTAAAGTGATCTTATCATGGTAAAATTAATCAGCATCATCAAACAAT 963791

963790 GTAATGATGAAGGTTAGAGATGATTGAAACCTTGATAAACAATTCATGTTAATTGACCAGCAACAGACTG 963721

963720 TCTTCTGCAGTTTCAGACAAAATATTCTAGTAAAAAAAGAACCTTAGTGAATTGATGTTTCACAAGGAGC 963651

963650 AAACATTACTATGAATCCAAAATAAGAGAAATTTAGTCCTGTCTGCTTTGTGGAGCAACTTTGCTCTTGA 963581

963580 TTCTCTTTACTGTGAGGTTTTTATGTCCTATTGTGATCAAGATAAAACCAGAAGCTGGGAGTACAAATAG 963511

963510 GGGGCAGATTGTAGGGCTTGAATTTCCATTTGCAGAAATAGGTACAAGTCTTTCAAGAGGTTCTCTGTAG 963441

963440 GTGCCTACCCTTACAACAAGTTTAAATATGTTAGATTAGACCTTTTGAGGCTTTTTATATCCTATCTCAT 963371

963370 TACATATTTTATCAGAAAAGTAATGTAAGATGGAGGTACCGAGGTATAATTATATGTATTACATGGAAGC 963301

963300 TATCAAACGGATGATTTGATTGTTTATTCCATTCCACCTTAGTAGTGTTAATATATGCCCAAAGTGTATA 963231

963230 TTATAAATCAGAACAATATTTAAATTTTTGAAGAGTTAAATACTATATTTGAAATAGGTTATTTACTGAC 963161

963160 ATTTTTTGTCAAAAGTTGAAAAGTCAAAATTTTGACTAAACTTTTGATGTGATTTTTTTGTATTATGAGT 963091

963090 TTGAAATTTTCTATGTTTTTCAGGTAAAACATTCACTTTAACTATAACAGTGTCATCAAACCCACCACAA 963021

963020 ATAGCTTCTTATAACAAAGCTATCAAAGTGACTGTAGATGGTCCAAGAGAGCCAAGATGTAAAACAAGTA 962951

962950 AGGATGGATTTAATTTTCCAATCGGTATACTCAAACATTTTTCCAAGGGTATGTTAGGTTTCCATTCATC 962881

962880 TTGATGTTGTCAGCTTTATCATTTCTGCTGCTTTATGTAAGCAATTTTTTAAGTTTATTTTACTTCTTGA 962811

962810 TGTTGTACCCATAGATGGAGCCCAAGCTTTTTAAGGATCTTACAATATTGCTCTCATCTTATTTGTTAAA 962741

962740 ATGTGACTCAAGCCTTCGCCTGAAATTGTTATTCCTAAAACATATTGATAGGGCACCAACCTCC 962677

>LgRunx gene (exons 2-5) [Lotgi1/sca_31:959283-963821](http://genome.jgi-psf.org/cgi-bin/browserLoad?db=Lotgi1&position=sca_31:959283-963821)

963111 TGATTTTTTTGTATTATGAGTTTGAAATTTTCTATGTTTTTCAGGTAAAACATTCACTTTAACTATAACA 963042

963041 GTGTCATCAAACCCACCACAAATAGCTTCTTATAACAAAGCTATCAAAGTGACTGTAGATGGTCCAAGAG 962972

962971 AGCCAAGATGTAAAACAAGTAAGGATGGATTTAATTTTCCAATCGGTATACTCAAACATTTTTCCAAGGG 962902

962901 TATGTTAGGTTTCCATTCATCTTGATGTTGTCAGCTTTATCATTTCTGCTGCTTTATGTAAGCAATTTTT 962832

962831 TAAGTTTATTTTACTTCTTGATGTTGTACCCATAGATGGAGCCCAAGCTTTTTAAGGATCTTACAATATT 962762

962761 GCTCTCATCTTATTTGTTAAAATGTGACTCAAGCCTTCGCCTGAAATTGTTATTCCTAAAACATATTGAT 962692

962691 AGGGCACCAACCTCCCAGTCTCCCATGTATGGTAGTCTTAACATTCTGCTATCAAATTAATTTCTGTCTG 962622

962621 CCGTATATTTAAAATTTATTCAGTTGAACTAGGAAGCTATGTCTTTGGACAGGGAATTAAGGATTGAAAT 962552

962551 TTTTAATTTGTGAATTGGGAACTGAATTTAAAGAAATGTAGAGTGGTATAAAAAAATATCTTATACTGTT 962482

962481 TTGTGAGGCTAGATTAATTGACTCTTGTAGTAGTGAAATGAACAGGTCATGACATATTTGATCTGAACAA 962412

962411 TTAATACTGAGAGAAAAGATGGTTTTTAAGAATTTAAATAGTAAAAAAAACAAAAATGTGACATTGTTAA 962342

962341 TATGATTAGAATGAAACCTCAATGTTATCAAAAGAAATCTTATTATCAAGTTTTTCTTTTAAACAGCTTG 962272

962271 GCATGTTGTCAGTTAAATGATTTTTCATGTCTTTCTACTTATAAAATATTTTATTATTAATCTTTACAAG 962202

962201 TCTTCCAAACTCACTTAACGCATGCCGTTTTTATAACTTCAAATCAAGTATAAATTTTTCAGAATAATTT 962132

962131 TAAGTGTAATGTAATAAATTTATGGTATGAAATGGATTGTTTTCTTTAACCAATGATTACACATCAAATT 962062

962061 TACAAATAATTCAAAACATTATATTTAATGGTTATTTTAAAATAATATTTTGAGCAATAAACTTGATGAA 961992

961991 AAGAAAACTTGATTGTTCTATTTTCACATTATTTTTACATTTTCACAAGTCAGTGGGGAAAAGTGACATT 961922

961921 TGATTAGTGGAAGAGAATAGAGGATAAAAAGTAATTGTGGTTATTGAGAGAGGCTATGATAAGGGTAGCT 961852

961851 GACTAATACTACACAGGAAACTGTCAATTGCCAATCACATCATTTAAACTTGAATTGTTTCACCTGCTTT 961782

961781 ACACTGCAGCTTTATACACGCCCATATATCAATAAAATATGTACCTTAGTATTGATATCATTAATTCTCG 961712

961711 TGTCTAAATACCTTCATGCCATTTGATAAATATAATACTGGATATCATTCTGCCATATTGATATAGTTCT 961642

961641 TTATCATTTTTTACCATTACTGATAGCATTTTAATAATCAGTGTGTATAGAATAATTTCTATATTATAAT 961572

961571 TAAACATAGGAATAATATAGCTTGAAAACACTCATTAATCAAATTCAATCTTGACAAGTTACTTCTTCCA 961502

961501 GACAATTTTAATTAATCAAAGTTGTATGAAGACTGATTTTCATTGTTGTCAAATATCATCTTTTATTTTT 961432

961431 CATATGCTTTCAATATCAGCAGGTATTTTTGTAAATTGTATGTTGTGTTTATATGATTAATGCCATGATT 961362

961361 TGTTTATTTAATCATTCATTTTAAAATCCTCAAAATATAATGAAAAATCATCAAATATGTGACATCCCAA 961292

961291 TATGGCCTTTATTTATTATTTACTTTCGTCTCTAGAACTTATACATACTGATGACAGAAGAATACCACTT 961222

961221 AGACCTGGACCATTAGATTTAAATATTCCAAGAACCACATTATCAGATCCATTAGCTGATCGAAGATTTT 961152

961151 TACATCTAGCAGAATTAGAATTATTACGTCGTGAAACCACACAAGCTTCAGAAATTCAAACAAATTCGAA 961082

961081 TGGTTTACGTTCACAAGCAGGTAAACTCTCGTTTTTGATATTTAGCGTCTAAATAGTCGATTTTATGAAA 961012

961011 AATTGATTTGGAGAGGATTTCAATCTCTTTAAATAAATTTTGTGTATTTAAAATTTTAAGAAATCAATCA 960942

960941 GTAATAACTATGTTTACTTGTATTTTATAGATAAATTATCAGATGCTAACCGAGGGTTGTGGTACGACAC 960872

960871 TTTATCTCATACAACAAAAGATTATAGTGTATCCAGTCATACATCACCTTTACTAAGTCAATCCCAACCT 960802

960801 ATACGAGTCACTCCTTTACCGGGTAGGTTATTGAGTGTTTAATATCTGTACTCATTACCTTTATCATCAA 960732

960731 ATTTTATATTCTAAAAACATGGCTACCCACAAACCATAATTTGACAAATATGGCTTCAGGCAAGGTTATA 960662

960661 CAATGAAGCCTTGATATTTGATGAAAAAAGAGTCATGTGACAGATGACAAAAAATTGTCTATTTGCTTTA 960592

960591 GGTTTTATATTTGCAAAAACAAAATTTCGTTAAATAAGGTAGCTGAGATTAAATTGGAATAAAGAAAAAA 960522

960521 AAACACCATTCTTCATAGATCCATTTGAAACAATGATAGTAAATGAGAAAAATTAGCATGTAAATGTATT 960452

960451 ATTATCTAGTTGCATTTGACAGTAATGTTAGTAAAATGTAATATAGATGTTTCTTTAATGAAACTGGTAT 960382

960381 CAGTTGTTTGCTTCTTTAACTAGTATTTTACCAATAAATTTGCCTTAAAGAAAATGAACTGATGAAGCTT 960312

960311 TTTCTCAGATTACTCAGCTTAAATCATTATCAATACTACCTTAATTAAAGATTGTATCTTTTTTGTGTAA 960242

960241 TTGGATGGTTATTAGAAACCAACATGTTATTATCATATTCTACTCAGAACTCTGATATTTTTTTGTATCC 960172

960171 AAGATAATGTTGATTATTGAACAACTTTAATTTGGTCATTTCAGCACGCCCTACAACTATTGAATCTCGA 960102

960101 GAAGCTGATAGCAGATTATCAGCAGTTCCACCACCCCCAGTCACAGATATCCAGTCTGTAATAACAACTA 960032

960031 GTATTTTACATCCTGACAGACGTCTACCAATATTACCAGACGTGACTAGACCAGATTATTCAGTCTTACA 959962

959961 TGAGTCTAGATTATCACAGTCCCAGTTACCACTAGTCTTACAATCTCAATACACCACATCTGCTGATGTC 959892

959891 CGACTTACAGATCATAGAATGAGTGAACCTATTTATGATACTAGATCATTATTTATACCACCCCAAACCT 959822

959821 TACCCTATACAGCTAGTAGTTCTAATTTATCTATTCTAGAAGAAAGTCGTGCCATATCCACATTACCCAT 959752

959751 ACCAATGACACATGGTAGCTATACTCCAATGTCACCACATGATTTCTTTAGTCGAATTAGTCCACCAAGT 959682

959681 GGTCTAACGTCATCACCATATCTTAGTAGTCCTCCAGCTAGAGTTTTACCTCCGACATTCCTGTATCCAC 959612

959611 ATCTTTATTCATCCAACTCATCTCAATATCAAACACGACTATATTTACCAACTGGGGAGATGAGGACATA 959542

959541 TGAGGTACTTGGACAGAGATCTGGTGAACCTTCTACAAGATTAGAAAAACCAGTTCCTGTTTCCCCAACA 959472

959471 GCTCGCTTAGCTCTAGAAGGTTCTACTTCATTACAAATGAGGGAAGAAGAAATGATGGAAGAGGGAACAT 959402

959401 CATCTTCTGATTCATCAAGGGATATTATACTATCTAGACCACCACAAAGATCATCATCAAAATCTCCACC 959332

959331 AAGACGAATAAATGACCCTGAACATAGTTCTGTGTGGAGACCTTATTGATTGAAGTTTTTAAAATGTTTG 959262

959261 AATCAAATGGAAATAGCAAGTTAGAGGGACTTGTGAGAGCATATCAGTTGTTTTATACTCTGACCAGAGG 959192

959191 TTTTGTTAATTGTTACCAAAGTGCTATAATTATTATATACCAAAGAGAGATTGTGTTAGAACTGTTCAAT 959122

959121 CAGCTATTTCTCTCATTTTGTGAATGTGTCTGAGAAATG 959083

>LgRunx peptide
MHLPTDLNGLTPFPDAHSAMGDVFPGDRPLSSILGEHPGELVRTGSPNFVCSVLPSHWRSNKTLPVAFKVVSLGDVKDGT
KVIIRAGNDENFCGEIRNYTAYMKNRVAKFNDLRFVGRSGRGKTFTLTITVSSNPPQIASYNKAIKVTVDGPREPRCKTS
KDGFNFPIGILKHFSKELIHTDDRRIPLRPGPLDLNIPRTTLSDPLADRRFLHLAELELLRRETTQASEIQTNSNGLRSQ
ADKLSDANRGLWYDTLSHTTKDYSVSSHTSPLLSQSQPIRVTPLPARPTTIESREADSRLSAVPPPPVTDIQSVITTSIL
HPDRRLPILPDVTRPDYSVLHESRLSQSQLPLVLQSQYTTSADVRLTDHRMSEPIYDTRSLFIPPQTLPYTASSSNLSIL
EESRAISTLPIPMTHGSYTPMSPHDFFSRISPPSGLTSSPYLSSPPARVLPPTFLYPHLYSSNSSQYQTRLYLPTGEMRT
YEVLGQRSGEPSTRLEKPVPVSPTARLALEGSTSLQMREEEMMEEGTSSSDSSRDIILSRPPQRSSSKSPPRRINDPEHS
SVWRPY*

>Sm-Runx1 gene

TGATTTTCCATTATTGAAAGTTTCACTCAAAAAAACAGCCGACCAATAGAAATCGAGTCT

GATGCAAATTCAACAGAAATTCAACCTGTGTTTTTTTAATTGGCTAATTTTCCTCACTGC

GGGAGATAAAGTTTAGTGGAAAATCCAGCGATTAATTGTGTCGAACAGCAATCATGAATC

AACGGTTCAACGGTTTCAGCACTGATTTCACCAAATCCAAGTACAAATTTAATAGTTTGT

CGGTTTCAACGGAAAAGATCGTTCCGACTTCCCATCCGTTGGTATTCTGCACGAAGCTAC

CTCAGCATTGGAGATCCAACAAATCTTTGCCGGCATTGTTTCAAGTGATGGTCTATCAAA

GCAACAATCGAGTTCTTCCTATCGGAGACGGACATCGGGTTTGGTTGTCGGCGAGTAATC

CGTCGATGAATAGCGCGGTGTTGAGAAATTTCGAATCGAAACTCGTCAACGGTGAAGCAA

GATTCAATGACTTGAGATTCATCAGTCGCAGTGGAAGAGGTTTGTTGAATTATTTTTTGT

TAGAAATCACTTTATAATCATTGTTGATAACAGGAAAATATTTCGATGTTTATATCCACA

TTGAGTGCAACCCGAAAATCATCGCCGTTTACACAAACGCTATCAAGGTTACAGTCGACG

GTCCTAGAGAGCCCAGAAACAAACACAGTAAACATGTTGTTTATTCAATAAATATAAGCA

TATTGAACTTCATAGAAAACTTTTACTTCACAAAGAACAACATAAAAATTCCCAACTTCA

TTCCAAAATCAGAACCCGACTCGCATTTCTGTTACAGCGGCTCTCCTGGGCTCACTGATC

CTCCGAGCTATTGGAGTTCAACAAAAACCGATACGAAAAATCTTTCAGGTGGATTTTTAA

TCAAATTCTAATTGATAATAATTAATTTTTAATTTCAGAAAATGATTCATTTGAACAGAA

ATCAAACTTGATAATCTCTCCGTCTCTTCTTTATCCATATGGGACGCCCAACGGTTGCCA

TTTCAGTTTACCTCAGACCAAGCAGGACAATTTTTCAATACTACAATTCATGCTGATGGC

AACGACACTTCGAATCGGGCTTCCTTTGGCAGAGATCTACAACTCGCAATCCCATCAAAG

GAGCCCCGTAAAAGAAATCAAAGATTTTGATAAGACCAAGATTTGGAGACCTTTTTAAAT

ATATTTGAGATGGTTATTAATAAAAGAAATGTAATGATGTATTTTCTACAGGGTTTTTAG

CAATAATTTTGTCATAAAATGGAGGGGGGCGTTTAGTCCAAAAGTGACCAGTGGATTTTT

TTCAGATGGAAAACAATCTAAAATGGTATCAAATTATTTTATAGTAATCGGGTATTTTAT

CCAAAAGCCAGGTTGTAGCAAGTGATTTTACCCCCCCCCCCCCCACCCCTTCCTTCCTCG

TTTCCGGGAGTTATTTGCGTAGTCAATTATTAATGCGCATACTTAAAACATATGGACTTC

ATTTTCAGTGTGA

>Sm-Runx2 gene

ATATATATACACACACTGACATACACACATAGACATATACACAACTGTTCTGTTGTTGTC

ATCATTGTACTCGTGTCGCTGTTGTACTAGATTATCATCCGGCTGACCAGCATTTATAAA

TACTCCAATATTTTCATTATTCTCGGAATCAACCACAAAAGTGCTAACAAGTAAGATAAT

TATGATTTTTTAATTTTTAGCGTAATAATTGTTATTGGTTGAAATTTTCCATGACGGTTC

GTGTTATGCTCTCTAGGAATCGAGAACAAATACTGCATGGCATCGGAATTGCTACATGCC

GAGTTTACGCTGAAGAGGATTTTGAAAAATACTGACTCGAGCTTTGTCATTCCTGGCAAT

CCGAACCTTTTATGCACCCAGTTGCCGTCTCATTGGAGAATCAACAAGGCGTTGGTGAAA

ACGTTTAAAGTCTTTTCGTTGTTGCCGGTTGCTGATGGGACCCAGGTAATTCTATCTGCT

GGAAACAATGAGAATGTCTGTGCTGAATTACGCGGCAATCATTCACAGATGAAGAATCAA

TCTGCGATCTTTCAGGATTTGCGATTTCTCGGGAAAAGTGGACGAGGTTATTCTTCTTGT

TAGTCTCTCTCTGTGTGATTATAGGGAAAACTATTAATGATATACTATTGATGGGGAGAA

GGGTTGCACTCAATATTCACCGATTTTTGGTCTAGCACTCCTTGCAACGATAAGTTTTTA

AACTTATTATTATTATACCTCAATTATGTTATTCAAAATGGAGCTCTAATTGCTTCAATA

AAGACATTATTCATCAGGTCGAGATCTATTTCCTTTTTTTCGAAAACAAAATCATTTTGC

TCCCTCCGTCACTTTCCTTTATTATAATTTATTCTCTTATAGAATTTGTTAATAATTTTT

CATCCACTATTAATTTTTTTTATTGATATTAGATTAATATTTGTAAGTATTTCATCTATT

ATTTGTTATTTTTATTAATAATTAATATTAAAATAAATAAACGTGAAAGAACCGTGCTGT

CAGAGCAAAACAGTAAATTTCAACGCAAATTCAGAACGAGGGCACTGAACGAAGTTGGTC

ATGAAGAAGGAGGGGTGAAAGGACGAATGAATTGCCATTATTACTGACGAGGTGGCCTAG

TCAAGGTACAAAAATCAAGTGAATTACTCATTGATCAGCATTCTGATGCGCAAAACATGG

CCGTAGGCTGTCCAAAATTAGACCCAAAATCAGTCCAATCAAATGAGTTATTTGGCTGAA

TTATCATTCTATTTGGTGGAAACTAGAGCACAACTTGTTCAATAGAAATGTCTAAATGTG

GTGCAGCTGGGATGGCTGCTGGCTGTCTATGAATAATGGCAGTGACTGGCACCATCCGTT

ATTTCATCATCAGCATTATTATTATTAGATATAACCCGTTTAAAATGACAACATTTTTCA

TATTTAAATTTCAGCTTTACATTATTTGTTATTTTTACGTCTTAGCGTTCAAATCAAATC

ACAATTCTCTTTTATAAAGCTAATTTTATTTTCAAAATTTTTATTATTAAACATGGAATA

ATTTTATCTAAAAAAACAATTATTAATTAATATTCATTTTTAACTTTAAAAATTTTAATA

AAAAAGTTCTCACAACGTTTTTGGTAAATGTGAAAAGTACACTTGAATACAAAATATTTT

TTAACCGATATCATTAAATTTTTTGAGCGTATCAATTTACATAAAGAATTTGAGTTTCTT

TTACTGAAAACAGCTCAATCTCTCAGTGTTACAGCCAGTGGGACACCGTAGGCAAATAAG

GCAATTGCCTTGGCCCTAGGGCACCACTATCATATTTTGATTATTATTATTATTATTATC

ATTATCATTATTATTATTATTATTATTATCATTATTATTATTATTATTATTATCATTATC

ATCATAATCATCATCATTGACATCATAATCACTCCTAATACACGTGGGAGGACATAGAGT

AACCACCAACGCTGTTATTCCGTATAATTTTTTTACATAATATTGTAAACATTTTAAAAT

ATATGTTGTCAGCATATGATATATTATTTATATTGGCATTTGAGAGGGATTGCATCTGAA

TTTGATATTAATCTTCATTGGCTGTCGTCAACTTTTTGATTCAAATTCTTTAAAAGAAAT

TGTAAAGATTTATTAAATTCAACTTAGGAAAGCGGTTCAATATCACAATCACAATGGAAT

CCTATCCGCCGCAGGTGTCAGTGTATGCTAACGCGATCAAGGTGACAGTTGATGGGCCGC

GGGAACCCAGATCAAATAATGGTATATCATGGCAACAGTGTTCCATTTTGATTGAGCGCA

TTGTCAGGAAGTTCATTGAAAGTTAATAAGCCTATTCGTCAATCTCCGTGTAAATTTCCC

ACATATAGTTCTCCTGTTATTGAAAAGCAGCTCCTTTTGGACGTCATTCAGCAGACCGCC

GACATACGGAGGCTCAATTTAAAACGACTTCATCCGTTATCAGACACAGATATTGAAAAA

TTTATTAATGGTTATTATTATTATTATTACTATTATACTACTAACAATCTAATATATAAA

TTTGCATTTTTAAGCATAGCCATATTTTCCTAGGCACTTGAACCTTTTTTCTATAAATAT

TATCTGTGAAATGATGAATTCCTCATCTCCAAAAGGCTTATATTACCCATACCTAAAAAT

AACAATAAGTGGTTATCAAAAGTATAGTTTTAATGAAATCAGCTTTAATTAATTATTAAC

TATTTGCATCCATTTATTACATACACACTGGTGCTGTGATTGGTTATTTATGAACTGTCA

GGATTTCAAAGTTGTAGAATGTGTGTATACGTTGCGTAGGTACACATTCTGTCATGACCC

CCTCCCTTCCCCATATACACTCGTACGTTAATTGTCCTAAATCCAACTACCTTCTCCATT

TCAGTACGTACGTAATATATGGATGGCCTCTTAGTTAAGTTTACTTATTGTCTGTCAATG

GTCTTTTCTGACATTTCAGAGTCTGAGAAATTAGTGTATTTGAAAAATAGGAAATACTTA

GCAATATTAGATGGCTTATTAGTTACCACATGCAACGTATAATATTAGCAGTTAATGAGT

CCTCGCTTCTTCAATAGGATGAATTTAGATTACATTAAGGTCTATTCAATTTGGAAACTC

AATTATACCGCGTATTTCCTGCTACAATTGCTGCTACAACCAATTCCAGTTTTACGGAAA

GTTCTTTCGATAACAAGAGAAATGGTTTGCCGAATCTGACAAAGACCATACACCAAAGAA

TGCTAGAAACATTCAAACTGTGATAAGGAAAATATAGAACGGAACTCCTGTGGTCAAAAG

AAAGCCAGAAACATTAACACCAAAAATAAATGTATCCCGAGGGCTGATATTATATAGGTT

CTCTTTCAAATCTTTTTCAGTATTAGCTTAAGGAATTTATAACGAATTTCAGCGGTAAAT

TCAATATATTATTATTTATTGTCATCATTACTACTACTTTCCCATTCATTTTTCTTTCCA

GATGTGTACAAACGGCGAAAGAAATGTCTTTCTTTATCTCTGATGACCTCGATAGACGAG

AGGGAATGATTAAGAATTGACAATTGCAATGAGCATATAATAGATATATTTGCAGCCCCT

TATTAGCTCATTCAAATGAATCACATTACCAAAGAGCTTTCAAAAAGAACTGCATAAAGA

TGTCAATGGTGATGCCAATGCTGTAGATAAGATAGAGCAGTTTCTATGCCAAGGGTGTTG

>TaRunx (scaffold_5|3941712|3956798; reverse complement; 15087 bp)

ATGGCTGCAATTAAAAGTTCTAAAAATGAAAGATCTCTTATTGACGCATTGGCGGAATAT

CCGGGAGAATTAGTCAGAACTGACAGTCCCAATTTTGTATGCAGTGTTTTACCATCACAT

TGGCGCTGTAACAAGAGTTTGCCTGTTCCATTTAAAGTCGTTGCACTAGGTTATATGCCT

GATGGCGTGGTAGTATCCTTAGCAGCAGGGAATGATGAAAATTGCTCAGCTGAGTTACGT

AATTCTACTGCGGTCATGAAGAATCAAGTTGCTCGATTTAACGATTTAAGATTCATAGGA

CGATCTGGTCGAGGTAAGTTGATAATGTATTGACTTAAGCGTGCAAAATGAAAGATCTCA

TCTATGGCGCTGGACACAATATAATACAATTTTTGTTCAATATTTAGGATTAGTTGCACT

TATTCTTGTATACAATCGCTATTAACCTGTGTATATAGACATATTTATAGATAGATTAAT

GCACTATATATTAAATTTTGGACAATAATTAGAAGATATAGCTCTTTCATACACAACACA

TGGTTGATTGAACTTAGTCTAAATAGATATAGAATTAGAATAACGAGCTTAGTAAATAAT

TTGATCTAGTTATAAAATAGCTTAACAACATATTTAAAAGGCATATCTTCCACTCTATTT

TTATTTACTCATAATGCATATTTTGAGTACTGCACGAATTTACTCTTTTAAGATTGATTT

ACTATTTTAACGTACTGATATACTATATAGTTAATACTAGTTAGAGCGCGTAGTATCTGT

TAATTTAACGAGTTTTATTCGGGATTAAGCAATCCATTTGTTCTTTTCCCTCCTATTACA

CATATATATACACAGATTCTCACTATGTATATTGATATACATCCGCTCTATTTCCCCCCC

CCCTAATATATGAATTAATTTAGAGCTCAGACGTAGTATGTAAGTAAACTATTATCCAAA

AATTAATTAATTCAAAATCCTTATCCGACTTAGAATTTGCGATTTTTCGGAATTTAATTA

AATCAATAAGAAGATGAAAGAATTTTACTTCGTTCTTTACAATCTTGTAGCGATAAAAGT

GATAGATTACCCATTTTTGAATAGTTTTTGGTAATTTTTTACCATGATTATCCAAGTCAT

TCGAATCTGTTGAATTAATTTAAATCAACGATTATTGTACTAGTACTGTAAAGTGTAAGT

GCATCCTATATGCATCAATATAATTTTTTTTCTGTTTCAGAAAAATCTAAAATTGATATT

ATCTTTTATGCTATCATGTGGTTTTGTAAGATATTTAGCATAGCGCAAAGTAAATTAATC

AACTTAATTTTCATATCTGAAGCTCGAATAATTAGACCTATTTTACATTATAGTATGCTA

TTCCTTCTAGGTTAACTAGTTTAAACTTTTTTTACTGCCATCCCTATACTAAATTCGATC

GATAATTAACAATGCTATATTTCCTTTGAATGATGATAAATTATTACTCTACTCTAACAT

TATTCAAATATAGACATGTTGAAAAACTTCAATGAGTTTGATTTCCTTTAAATGATGATA

AATTATTGCGCCATGCGATCTTAATATAACCAAATATGGATAACTAATTTTAAAAGAAAA

ATTCATCTAAACTAAACCGTATTTACTTTCCTATAAGTGTTCATAAACTATTGTTCCATA

GTATCACTGTTGCCTGAAATACAGACAATTTGAACTTTATTCAAACTTTTTTTATCTATA

TCTTCTAGACCATAATATAGTATTACTTGAGTTTATTCCCATATATCCAAACATCGACAC

CTTTAAAATAATAGTAAATGAATTGCTAATAGCATTAATAGCCTAGTAATCGATTTCTAT

AGTATGACCGAATTTTGATGGTCGTAATTTTATTTTATTTAACTTCGCATTAATCATCAT

AACAATATGTATAGTTAGCAAAATTTTTTTAGAAAATGCTTTCATCAATAATGTACTCAA

TAATAACTTAAACCAAGAATCGCATTCTAGTCCATCAATTTAATCCTTTTAATTGATCAT

TTCTACTTCTTGAGTTTTTGTTAGTCTTACTATAAGGCTTTCTTTTGTTGATCTTGAGTG

TTTCCGAGTTTCAAGCTATACATCAATCGAAGTCGAAAATTTTATACCCGATTGTAGTAG

AAACATTCATGCAAATTCTACGATAGAAACCTTTTTTCGTTAAATTTAAATACCTTAAAG

GGATTCAAATCATTCCATAGTGTTACTTATGCTTGTATTTAGGAATAGTTCCTAAATTAA

GCAATTTCAACTCCGAGTATAGTCAGGATTATTTACTATCTGCGTTATTCTTTTACTTTG

ATCAGTACAACTTATAGCTTTAGCTATAGAGATGAACATTTGTCCTCTTGCTGCAAAATA

ATTACACGCACGATGGTAATAGTCATCGTTTTTGAATCACCCTTGCCTAAAACTAAATGA

TGGCTTTTAAGTTATTCAATCGATTAGCTGATTGATGTATCATTTAATGGAAGGAACTAT

AAACAGATGATTTTATATGTAATCAGTGATCTAAATCGACATTACTGGTATATAGTAAGC

CTATTGATCAAACCAACATATCAATCGCACTATTGATTATTGGACCTCTATATGCAAGGA

TTTTAAAGTAGTAATAAATGAGCGAACAAATTTGAAAATGATTATCGACTTTTATCTTGT

ATTCTTTAGGTAAGTCATTTACGTTGACTATTACTGTCGGCACTAATCCGCCACAAGTTG

CAACATATAATAAAGCAATCAAGATTACAGTTGATGGACCCCGAGAGCCGAGAAGTAAGT

CAATAATGCGATGTTAATTACAGTGTAATACTGATATTATTCAAATATACTTAGATTGCA

TGGTTAGTTTTTCCTTGTTTACGACACTTTCTTCGCTATGAGTATTGAGATTTCTATGAG

ATAGATAGCATTAATTGATTTCTTGTCTTACAGCATGAGGGTGGTATATCAGCTAGAATC

GATCTGAATCGATTTTAGAGATGAGGTAATTGAGTCTTAATACTTATTAATGGTTAACAG

AATGCTTTTTCGTCTTTAAAAGCTATCGTATGTCGAGAGGTATCTGCAGGGAGGTGTCTA

CTTAATGATCTTCACCTCAAAGTTATACTAAAATATGTTCTTGAAATTGTCATAAACGGG

TTGATAACTTCTCCTATCATCTGTAAATAGTCCCAGCAATATCTAAAAGGAGTCAGTATG

ATGTTTTAATAATGCGTGATCACATACAATATTACACCGTGCAATTTCAAATTAAGAATT

ATGAGTAAAAACTGAATCTTATTTCTGTTGTGTCTACGCTAACGTTTATTGAAGTAACAA

TTGTTAACGGTAGTATTATCGGAAACATTGGCCATTTCTATCTTTTACTACATTACTTTA

TCATTGTCTTCTCTACAGTATTACTTCACACATGGTAGATGTTCACAATTTTATTATAGC

AAATCGGTATCAATAGTTTTACATCACTTTCCAAGGGTAATGAATTATTACTATTCATAT

CACTTTAATTTTCGTTAGTCTCACTTTTTTCTTGAAGGAATCAACACGCTAGACCACATT

TACACTTTAAAAGTTACTCATACGTTACTTAATATTAATTCCAATTTCAATTAAATATTC

TCAACATTGGTCTCTTTCATAGTTCAATAATTTAGCCTTCAAGGCTTTGTTAATAAGTTA

GGTTGATATCCGACATGAATGAATGATACGTTATGTAGTAAAAATATTCATGGCCTCCAA

AATGGGCATAACCATATTGACGATACGCCAGAGTAGGCTTTGGCGGTTTCCCGATCTTTC

ATGATTAGCTGTCATACCATTTATTGAATCTGAATATTTCTTGAATAATCATCTTACCGC

AAGTAAGTACGAATATAATAATAATAAAGAAATGAGGAAGGATAATTTTGTTACTCACTA

TGTCTAAAATTGGGCGATACTATTATCAACTGATCGAATAGGTATACAAGCATAAAAAAG

TGAAGAAGAAATGATTATTCTCTCTTCCACTCGCTGACGAAAATGTAACTTATTAATCTC

CATTTTCGTTGAATAATTTTGAAAAGTTATCCTCTCCTCCCACTCTTTCGTCATCTAGTT

AATATCCTTATTAAAATTATAAAAGATGCCTAGTTGCAGTTGTATACAGATTTCCCCTTT

TAATAACTCTAATACAAAAGAAAGTATTAGAAATGACGACCAAGGAATCGTTATTATCCA

CGAATTAGCTCATTTCAATTGGATCAAGAAATGAAGGGGTCGATATGTCAATGGATCAAA

ACACATGTTATTTTTAATGTTGGTCTACTGTTTTAGCCAATACTTTTATTCACTGAATGA

ATAGAGTAGACCAAAACTAAAAGTCTCCATAGTCATTAATCTTCCTCTGTAGTACCATAA

ATAATATCACTGACCCATTTGCCATGACTACCGATTATATTTTGATTCTATTTAGAAATG

GCTTATTTTACTACCTAAGTTTATTAACTTACTTTATTCATTAACTGATTGATTTACGAC

AATTCCATATTACCGCACTGCAGTATCCCAAGCGTGTTTGTATTAATATTATTTATTATA

GATTTTTAATTTGATCCATTTCTTGAATTCTATTTTACGTATCATACTACTGGTATCATT

GATATCAATATGAGAAAATGCATTCCATAGAATGCTAAAGCAAAATGCTATAAAATACAT

CGCAGAAATCATTTCCTGTAGCAAACAATACGTACAACTTAAATTTATAATAACGCCGTA

GGATGGTTTAAACTTAGTCAAGAAAGAAGTCATTTTTTTTAATATCCTCAGGCTGGTCAT

TTTATGAGTCATAATTGCAGCTAAGGCTATGATGAGGTCATTCACCGACAGACAAGACGC

TTAAAAAAAAGAAAATTCTAAGTAAATAAGACCACAACAAATAAAAATTTAATCAAAAAT

TCAGCTACTATTATTGAATGACGTCTATTTCATAGTGAATGTTGATCATTATATCTGCAG

GGTTAATAATTTTATTTTAATCAGAAGGACTAGCATTAATGATAGGATAGCAACACCGTT

GTTTCAATTATTTTATTTCCTAATATTCACAATAAATTTTACAGTTGATTTCGTTTTCCT

TTCTCTATTGCAATAAGATTATAACTTGATTTCATGGTGATGAGTCGGAGATTCTATTAT

GTATAAAATTCCAACTATCCACAGAATGTCAATTGTTTGGTCAGTTGGGGGAAAAAGTAT

TTAATTATCCTTAATTAATAGAATGTGATGACTGTTATCACTTCAAATAGGTTAGACATA

AATTAATTAAGACTTATGGCGTACGTTTGATTCAATTAGAAAGATGGGAAGATTAATCTC

GCCTAAGGTCAAAATCTCCCTTATAATCATCTGTAATTTTAATTTAAATGAATAAATATA

AAATTGATTGTACTGTGGTTACTATGTTGACGTTGGAGATATATTTATAGGCTTTAATAT

CATATCTAGCTAATACAGTATAAGTAACCTGTGAAAGAATTAATCTAGGACTATTATAAC

CACATTTCTTCACCTCAGCAATTGCTCCTTTCTTTTTTTACATGATGACATCGTATACCA

TACTTGACCTCAAACACATGGGATCTATTGTATTTTATCCGAAGGCAATATAACATTTTC

GCATTTAGTTATGGTTAAAAGCTAAATAGAAAGAATATTGATTAAATTAAAATCATGCTC

AATAGCCATTTTTTTCCTTCGTTGATGCTACATTTACCTTATGTATTAACAATTTCTGTA

AAGTCTACCCTATAATCGAAAGTAAACAGGATATTAATGTACAGAATCATGATAATTTAT

ATCGGGTAATTAATTTAGAATTTATGTGTGGTGTTGGAATCTTGCGTTGTGTCTGAAGAT

TTTTATAGCTTCTATCAGTCTCAAGTATGTGGAACAGTGTTTGCCCACTCATGACGCAAA

TTAGGCTTTCTATATTTAATCATATCAACAACTCATTATTCAGGTTCATGGTGACCATAT

TAGATTGATTTTTTAATATGACAATTTAGTTAAATTCTATTCGACTAGATACGTATAAAA

ACGAAATATTCAATTTTGGTATTTATAAATGTCCACTTAGTACACATCAATTGAAACTAC

TCTTTTAGTATTCTATTTCTCTCTATACTGATGTATAAGATCAGAAACTGAGGCTAAAGT

ACAGTTATTTTCCATTATTTTAATTAGAAATACTATTGTCACTAGTTGTAATGAATATTC

TTCCGTGTGGGACAAAAAATTCACTCGAAATTAATTTCTGAAACTAAAAGCCCTAATAGG

GAGATTCTCGTAATAGTAGAAGAATAACCCCAGAAGAATAGGCCAAAAGTATTGAAATGA

AAATGTCTTAGACTTAATCATACGATGAATAAATTGATTAATTGATAGCTGCCCAGGCTA

CATTAAGATTTTTTGAAGCTTATGTTGATACGGATAAAAATACCCAATCCTTTTTATACA

AATGAGCATTTAATAAATAAAAATCTTATTGAATATATTTCACATAGAATGGAATCGTAG

GGCAATTAATGACCGAATTTAATTTGTTTATTTAAGTCTACCTTTATTGCAATTTAATAT

AATTTCGTTCATGACTATATACGGTAGTAGAATATAGGACGATTCATTATAGGGAGTCGA

AGATATACCATAATTAATCATCATACAATATGGTTGATGAAATAGTAGGGCCATTCCTAG

TAATCATAATTATCAATGAAATTTAATTAAATGTAAACTCCGTCAATGGAATTCTTAGCC

AAGAAAGAGAGAAAGAGAGTAATGTATGTCTGATAGTTTTAACTAGTTTATCTAATTAAT

TTTAGTACGTTATTATTACAATGATTTATGATATGCAAATAATTCTATAACGTCAGAAAG

TTGTATATAGCAACTAAATGGAGGAAGAGAATGCACCCAAGTACCGCTCATAGGCACGAA

ACTTTGGCTAGCATTCATTCTTTTCTATTTTAATGAGATTGATCAACTTTTCTAAGGGCA

AGAATTTCTCATTCTGGGTTAATAGTTACGAAAGATAGTGAGTACGTTTATCTATATCTA

CATGATTTTGATTTTTTTATTCTAACTGTAAATTTATTTATCGCCTTCATGGGATATTAA

AAGTAACTATCTAGACCTCGAAAACTCAAAATAGTGATCAGAAAAAGAGAAAAGATATCG

CTATTTTTATAATATCGAATTCTTTTACTGGAATATCTACAGTATAGCACAGACATTAGA

AAGACTGGTTCCAATATAATAAAATGAGTAACTTTCTTCAATTATTTAGTACTACGTTGA

TGATCTGAGTGGTAGTTAGTGTATAGATTAGGCAGGATCAAATTTGAACTCATTGAATAT

TAACATTATCCATACGTAAAATGGGGAGGACGTGTCATGACTCATCGTTGATATAATTTA

TTTTGAAGTATATGTCTGATCAACTTGACTGATTTCAACTTGATTCATGATGAAACTGGT

AGATATGAAAGTCCAATATTAATATTTTAAAGAATCAATTTATTAAAAATTCAATGGCTA

TCCACATGATGAAATACGTCTAAGTTTTAACTTTTTATTTCTAATTGCCTTCGGCAAAGA

GGATCAATACGTAATGACAGCTATAACCCAAAAAGGAGATGATTAAAAAATCAATCCATT

TTCGTATAATTTAATTTGCTGACATTTTCACCTACGATGACGTCAAACTTCTATATAATT

TCATGGTGTAACAATATAATTACACAGTAGAATAGGCAGGATAGATACAACAGTTAACCA

ATGAAGTATCCGTTAAGATATCTGTTGAAATCAGCTTGGGGAAATAATACCAATTAATAC

TCGCGGGAAGTATGGGAGGAAGAAAGATGTTGTATATATTGCTATTTTCTGTCTGACTTG

CTCTTTCCTACTCTTTATCATGAATAACATTAAATACGTTGATATCCTATTTTACAATAT

TTTTATATGGCATAGTGTATAATTTTTAGGCGTCAATTCTAGCACTTTTCCTACATGATA

AAGTACCAATTTTCCTACCAGTAGTCATTTATTTTCAAAGTATTACTTGTCTATATCAAT

AAATAACTTACGCTTTCCTTTCTCTATGATGGGGATAATAGAGATCATGGTTGTAGTTAT

ATTAATTAGATCAATGGTTAATGTTATCTTTGACCGCTAAAAACAATTTTCTTTTTCAAC

TTGATCTTTTTCAACATTTTAATTAATGTTTTATTATTATAATAGACAATAACTATCCAT

ACCAAAAATCTATATTTACTCATGACACCATCAATTGTTCTTGTCCACTAAAAATTTCAA

TACGATGTTGAATTTCGTTAACCACCCGTGTCTTACTACATAATAGCTAGTACAACACTC

TTCCGATTCGCTTAATTCGTTTCCAACCACGATATTTACTTTCAAAGCAGGTGTTTGCAA

AAAATATATAATTAATGTAAATTGCGAGTAACTCCATTTGCGTAATGCATCTTATCCACC

ACTCTTTTATAAACAACAATGACAAATAAAATTCTTCCTAAATTTTTCATACTCTCTCTT

TCTCCCTCACTCTTTCTTTTATTAAAAAGAATTTCCTTATTTTCTATCTTTAGTCCAATA

TTTCTATCTGTAAATATCATAAAATAATGAGTTAATCCTTCCTATCCATCGTAATATAAT

GTTCAATAACAAAAATATCCAATAACAATGATTGTACTATTCCATCGTCTAATCAATACT

CCATAATACTCCTCTACTATCTGTTTGCAATTTCTTAACAAATGATGACACTTTCAAGTA

AGTTTACTTTTCTATTCTTTAGGAATATCTTTTATAATTTAAATTATGTTAAAAAGAGTT

CATAATTCTCTTTCAAAAATGTATAGAATAACGCTTCTGTCTGAATACTATTCCTCCTTT

TTTAAATGTTTACAAATTTGATTAAGAGTAATACTTATACATTATGGTAAACTCTTATGT

TAGACTCTTTGTCAATCCAGCGAAAAGAAGTTGGAGACGTCAGCTACTCATCTTTACATG

TATATTTGTACATATTTTATTACAGATGTCACCTATTTTTAACCTAAAAGCCAATATAGA

TAAAAATTTAATCATAATTTAGCTTACAAATGATCAAAGCCTTTTTAAAAAATGAGAAGG

TAATATTTTATTAGCGCTCTCATAAGCGTTGAAATATAAGACGAATTAGTCATAATAGAA

ATAGTCCAGTTTGTTAAGACTTTGAATATATACCATAGATTTATTGAGACTATATAAATA

TTTTTACCTTTGGTTATGATATAGTAGAGTAATAAAAAATAATTTTAATTTTATTACTTG

GATATAAAAAAGTGATAAGAATAATTTATATCACGAGGATACTTCGTCTGGAATCCCTCT

GGATAGATCAGATAGTATTATTGCCAATATTTGAATATACATATATGGATTTAATTATTT

CGGTCATACGAAAGAATAGCGGATGAAATATGTCCAAATCCTGTTTATTATTTCTACTGT

TAGATATATTCACGTAGCTTTAATTATCCTTTACAGCTATGGATAACCAAAATTGACAAG

ATCTTTGATAACATTATTAACAAGGCTAATAATGGCTCATAATTGTTTTCATCCAAGTTC

TAGTTAAGATTTTACTAAAATCAGATATGTAAGAATACTGGGTAATATTGAAAAGATATT

GGGTGAGAATAATCACAATAAATGAGATCAAATTCATGGTAATCCTGCCCATCTAATGTC

AGCTATATTACCCATGAATATTAAATTTAAATAGATCAATTTTCACATAGAATTAACTTA

ACTAGGATATGATTACTTTTTAATGATGATTATGTAAATCTAAATAAAATACAGACAATA

TTATTGTTCTTTTTCTTTCAAAATTGAGCTGGATATTACTATTATTTTACAATGGCGATC

GCTTTTATTTCTCTATTTTACTGCCCATAGCGGATCTATTAAGATAATAGCGGGATCATA

ATCAGCTACTAGAGTAGTGCATTGGCTGATAGTTAGCTAATTTATTATACTATCATACTG

AAGAATTGCAATAGATGGTAATGGGAGTAATATTGTAGATATCAAGAGAAAGCTGCATAG

AAAATCTAGACAATAGAAATACTACCAACAACCAAAATTGCTTTTGATGAAAAGGGTCTC

ATATCATTATTGTTATTACATCAGCCTACGTTGATATGGCTGTGATTTTTTTATCTTACT

TGATTCCAATCTTGACGTAAGTTTTCTTCCGCTTGGATGTTATTGATTATTTAAATATTA

TTTTACTTTAACTCTTTTGACTGACTATTCAGAAATTAAGCAATTGAGTTATCTCGTATA

ATCACTTATTCTTGCTTGGTGCTAATATTATGAAGAAAAAGGTTTTGTTATACACTATTC

AAAGCTAAAATATCTATGTAGCAACTTTGTCTGTAAGAGAGCGAGATGATCGCCATGAAA

ATGATCAATAAGGTCTATATTAGATCGAATTTTTGAATTAAAAAGTACTATTACCAGTTA

CTACATTCTATAATGCAATGACTAGTTAATAGATATACTTGAAATACTTAGTTAATTTGA

ATTGAGTTAAGTTAATGAATGGATGGATATAAGTAACACTACACAGTATATTAGGATTTC

TACTGCATAATTGAAATTAGGGAGAGTTTAGACTATAAGAGAAATTATATAGTAAAATAT

TGAATGGTATTACTATGCATGTATGTAGATATGAGGAGGTGGGGAAATGATTTAAAAAAT

GCATTGCATTACCGTCATCCTGCTCTATTGACATATTGGGAGGCAAATTTTATCATCCAT

CCTATAATCATTGATATAATATCATTTATGCAGTGTTGCTTTCCTCTTTATTATAACGGG

ATGGATTTCAATCTCATTTACTTATGCGCAAATGTTACTTAGTGTACTATTAGTTGAGAT

AAAATTAAAATAAAATTAATATATTGGTAGCTAAGCAATCTAAAAGTATTTGCTCCCGTT

ATCATCTAGGAATACGTAAATTAAATCTACAAACATACCTTTACCCCCTACAAAGTATAA

CTATACCCGTTAATCTACATATGAATTGGATTACGATTTTAATAGAAATAGATCCAAAAT

AGTTTATACTTTAATCCTAAATTAATAATACAGGATATTTTTAATTACTTAAATATGAGA

AATGAACTATTTAGAAATCAGAAGTACAGTATAAAATATTTAAAATTTGGTCGTGAATAT

TGAACTAGTTTAAATATGTCAAGATGTGGATGTCGAACTATTCATTTTAATTCCCTTTCT

ACTTCTCCCCTTGATGTCTTTTCTATCACTTTGCTTATGCCATTGTCACATTGGCAACAA

TTATAACTATGCCGTCGTTATAAAATTCTTATTTTAATAAGGATAAAAGATATTTAGTAC

AGTATTTCAAATTAGCCTATGTTATATCGATATAAAGGGCTTTTCAACTCAGAACTAATG

AGTACTATAATTGGAATACCGCTTGTGAAATTAGAGTTAAGAAATTAACTTTACTCTTAA

TTATTGCCAACTTTTAACTCTAATTTTCAATTTTATGAATTCTTCTCTTTACGAAATTTT

ATTTACTGAATCATATTGTAATATTGACAGTTCACCAATGACGTAGAATGAAGATGTATT

GATAGAAATAGTAATCGATAAAAGTAACCTTTGCATAATCATCATCATTATCATGAATAA

TTCCCGTTATAAAGTAATGCCAAAGAATAACAATTTACTTATCCATTCGTGACAAAAATG

AACAGCATAGCGAGACAAATTATTTTCAATTTTACATCTATTTAAAAACAGACCAATATT

ATCAAAGTCAACTTAAAGTTTGCTAGTAAATGTATATGTAACTGATTGTTTTCATTTTTG

TCAAATGTTCCCTTTAGGGCATAAAAATAAAAGCTGCCATCAAGATCGAAGAGCGTTTGA

ACATTATCATTTTGCAAATCGTTTTAACAATGGTTTCAATGGTTGGCGCCGTCACTACGA

TCACATGCCAGGATATGACGACATTCAACATTTAAGAATCCATGATAATATGATCCGTAC

GTCAGCATTTACTGCGATCAATCAAGGTAAGAAAACAGTCGATTATTAAAATTGCTTGAC

CTTTACCTTAATGGTTGATGTGGTTAGACAATGCTACGTTGAATGATATATCTGAAAGAA

GATTTGACATAGACTCTTTTATCCCACGATTGATTGACACATCCGGTTAAAAATCGATAC

CAAATGACATACATACTGCAATAAAATCTATTTAAGCTGCTAAAAATTATTTTGGCCTAA

ACAGTTCTAGTCAGTTCAAGGCTGTCTATTTCTATCCCCCTTTCCTCTAAATTGAGTAGG

ATATTCTAAATGATACATTGTATCATTCACACTTTTAGCCCCAATGTGATCATAAAGACA

GATTTTGAAATGATAATGAAATATGGAGTCTACTTATTTTCGTTTCATTAGTTCAATCTC

ATTGTATACCAACATCAAATCGATCAATTAGATTGCCACAGGAGGATATTAATATAAATA

AATTAATACTAGAGTTTAAGTATCGGTAATAGTCAGACAATAGCAAAGTTTAAAATACAA

CAATACCCTCAATTTACTTATTACTATCTGCTAAATTAATAGGTAATACCTATGTAGTGC

AAATAAAATAAAAATAAGATTTATCCTAGTATGAAATATTTAGGAAGTAATATGATTTCT

CATTATGTAACAGAATTATAATTGTAATACTTTAGGCATATTTTAATTTTTTCCATTCTT

TACTTGACAGACAGCTATTTTATCATACATTTTACATTAGTTCCAAGGGTTACTTAGTCA

AATGCTGACATAATATCAGATGTAATGACCTAGAAAGGATGTAAGAATTGAATTTATTTA

TTTATAAATTCTTGACAATAAGATATGAATTCTATTTTTATGATTGAAATTTACTTATCA

TAATTATCATATTCTTTGGTTTATTCATGCAACGATAATATTAATTTCATATAACAATTA

AAAATTTTATGTATTAAAATTCGAAAAAAATTATCTAATGTGTAAATTAAGTAATAATAT

TTAAATATGGAATTTAATCTATAGTATACTCATACAACTTGTTGGGAGAATAATATTGGT

CCTTGTATGACATTTTTTCAGCAAAAATTTCGAAAATTAACGAAAGATTCTTTTAATCCT

AGTTCCAGTAAAAATAGCATTTGAATATGGAATTGCTTCCCTATACTGTATAACATATTA

TAAGAATAATAGTTCTCATTGTCTGACATTTTGGCACTGAAATTAAAAAAATAACTGAAC

GTGTAATTCATTACTAATTCTATTTATGTCTTTTGAATATAGAATTGATTTTCTATAATG

TATAGCCAGTTATGGAAAACGTGTTAGTCACTTTCTGATTTTAGTCTAATTCAGAACAAT

GACAATGTCAAAATTTGTTTTTAGAAAAATTAGAAGCTATAAAGCTTATATCTTTCTTTG

TTACTGATATCATGCAGTAAAGTACCTGGAGTAATTGATATAGTAAATGCAGTTCATATA

TCGGCGTATATAATATTCTAATAATAGAAAAATTTGCATGACTTTTAATAGATTGTAATT

ATACTTGACTATGTAAACTATGTACCAGAAGTCTCAAAATCTATATTATTGTAATATTAG

AACACTAAGAAGCATTACATGATTACATTTGCACGTAATTAGCGTATATAATAATCAAGT

GAAAATGTAATCTTTTATAAGTTATACCAAGTCATATCGTATTTACTTTAAATATAATCT

ACCTAAACGGTCATAATTCCTATATAGAGACGGTTCAAATTCAGTCTATAAGTGTTCTAT

TGCCAAATAATTTAATCCAAATGTTATATTGACACAGATTGAAATTTCCCCATAGCACAG

TTATTAAACTTAGCGAATGAGAATCATAATCTCTTCTATAGATTTTTTCGTATTTTAATA

TTGCATACATGTGATACGCTGGGCTATCCTATTTCTAATATTTATTAAGATATATTCGCA

CGCAGTTTAACAATCTAACAATGCAAATTAATTGCTGATTACGTATTTATAATAAAAATT

CAGATAACTATTCAATAATTACCATTAATCGTGAGGCAGCATGGTTAATTTCCGTCATTT

TGTAATAGATTAAATATATAACAACTACATAGAATAGCCGAAATTCGATAATCACTTTCC

ATTCAATGTATTTATCCAGGTATTGGCAAGCTACCCCATGCAATTCCAATGCCTTCTACA

GAAACAGTTTATAATTCCTTAAGAAATCCAATGGACGTTATGCCATGCCATCCAAATAGC

TTACCACTTAACAAAACAACTCCACCTGGAAGTAAAATATCATTACTACCGCCTCATTAT

CGTGAATCAAATCCTACTACCGTTTCCAATTATTTAAATGGATGTCATTTAAGATCTCCA

ATGAATAATCCTTCACTAATTCGCTTAAATTCTACTTTTATCCCACCACATCAGCAATCT

GGACAATATCAGGCTGACTTAATAAGAACGCCACCTAGGCATGAATACTCACCAGGACAT

ATACCCCTTGTAGCTGCTGGACTTCGTGTAACCAATCCTAATATGACAATGCCACCCTGG

TTAGCCCATATCAAAGCCAATGGTGCTAATATGGCGGCAGTGGCTAACGCCAATAATCCT

CATCAGCTTAATCAAAATCAAACTAGTAATAATGACAATGTATCAGTTCGTAAAATTGAT

CGTGAAAAAATATCAAAGCCCTCTACTACCCCAACTCCTCATGATACAGAAGACGGTAGC

CAACAAGAAAATAAAATAGAATCTGTCTCAAACACTGATAGTGATAATAGCAGGCCATCA

TCATCTTCGTCCAATCAAGGCGAAATCAAGAATAGGAAGAGAAAAGCAGAAAGTCCGGTA

GCTAAGAGTGTTTGGAGACCATATTAG

>TaRunx peptide

MAAIKSSKNERSLIDALAEYPGELVRTDSPNFVCSVLPSHWRCNKSLPVPFKVVALGYMP

DGVVVSLAAGNDENCSAELRNSTAVMKNQVARFNDLRFIGRSGRGKSFTLTITVGTNPPQ

VATYNKAIKITVDGPREPRRHKNKSCHQDRRAFEHYHFANRFNNGFNGWRRHYDHMPGYD

DIQHLRIHDNMIRTSAFTAINQGIGKLPHAIPMPSTETVYNSLRNPMDVMPCHPNSLPLN

KTTPPGSKISLLPPHYRESNPTTVSNYLNGCHLRSPMNNPSLIRLNSTFIPPHQQSGQYQ

ADLIRTPPRHEYSPGHIPLVAAGLRVTNPNMTMPPWLAHIKANGANMAAVANANNPHQLN

QNQTSNNDNVSVRKIDREKISKPSTTPTPHDTEDGSQQENKIESVSNTDSDNSRPSSSSS

NQGEIKNRKRKAESPVAKSVWRPY

>Amq-Runx gene (assembled from traces)

ATGTCTTCTGAATCAATCCCACCTCCTGATGGTCCCCTACCCCCTCCCTCCTCCAAACGCTATCGAGGAGAAAGAACCTTCTCCGAACTATTAGCGGAATACCCTGGAGAACTCGTAACGACCGATAGTCCTAACTTTGTATGTACTATCCTTCCGTCCCACTGGAGATGTAATAAGACCTTGCCCGTACCGTTTAAAGTACTATCGCTCTCCGATATCACTGATGGTACCAAGGTGATACTAACAGCCGGTAACGATGAGAACTCAGCCGCTGAGCTTAGGAACGCCATAGCAACTTTTAAGAACCAAGTGGCTCGGTTTAATGATCTTCGCTTTGTCGGTCGATCGGGAAGAGGTGAGAGGGTGAGGGATGTAGACCATCATGTATTATTTTGTGTTAAGTGGCCTATCATTCATAACCCATTATTGCTTTGACTAGTTGTAATGGGCAGCAATAGATAACTGGCCATTAACATAAGAGGCCATTAAACCACTTACTGTCTGCCCTTTTATTTGAACAAGTTTATAAGCATAATGTACAGTCTAGCTTCTATAATAAGGACACCTTTATAATGGGGACATTTTCTGATGTCCCAATTGGTACAAACCTATACAAAAGTACCTCTGAAATGAGGACACCTCTGTATTAAGGACAGGTAGTGTGGGCCCAATGGTGTCCTTAATAAAGAGGCTACACTGTACATGTATTAAGGACAGGTAGTGTGGGCCCAATGGTGTCCTTAATANAGANGCTACACTNTANATGTANTAAGNACANGTNGTNTGNGNCCAATGTTNACAATATATAAAGTAGTGTTCACACACATTGATTCACACAATAATATTGTAATTNNCTATCATTAATTTATTAATTTTAATATTATAATTACAACATTAAAGCATGTAAGAATTAATAACGAAATAAAAAAATGAAAATTATAATAAATTTATTTTCTATTTAGGTAAAATGCTGACAGTAACAATCACCATAGTAACAGAGCCGGTACAATACGCTACTTACTCTCACGCCATTAAAGTGACAGTGGATGGACCAAGAGAACCAAGACGTAAGACACTGAGAGAGAGAGGGAGAGAGAGGGGGGGGGGGAGAGAGAGGGGGGATGTTAGGTGTATATACATTTTATGTACATGTAATGTGCAGTTGGTAATTTTTTGGTAAGATTAGCGGAAATTTTGGTCGTTGGCAGGTTCTTTTAATTACATGTACATGTACATGTATATATTTAATTTGGTGATTTTTGATGTCTGCATTACTTAATGTCTGTTACTGATTGTCTGCTTTTAAATATTGGAAAGAGATATACATGTACATCTAAAGACTTGAAATCAACAATCGTTCAATGTATATCCTCTACAATGTACATGTACTGTAGCTTATCCTTTACAAGTTCATGTACTGGACTCAGAGGAATTAAGATAATGATACATGAACTGTATGTGTGATCATCATTGATGTACTGTTCTGTATTGTTAGGTGAGATCAGGATGTACTGTGTTCCAATTGTCACCATTATATCATTATCATAATAATGAAATCTTTTGTACTTGTCTCTCTCCCCCTCCCTCCCTCCCTCTCTTGCACACATGAAATGTAAAATGCTTTCATGTTTAATGACCTGTGACTCAAATTGTAAAGAATGACTTTAAATAGCAACACACACACACACACACATGTGTACATGTACAATGTATAATGTATCCTATTGATAGAAAGTGTTTATTAATATTGTAATGTTTATTAAACAACACATTGCTTTATAATGGGAGACAAATCACAGCTACTACAACTAACAGTACATGTAGTGGGGTAGGGCAGGCCTAGTCTACTAGTTCTTTTATACTAATGGTACACTATCACAAGTCCTACATTCAATTCTTGTAGACTGTACGTGGACTTTAAACTGATGGTACACTATCACAAGTCTACATTCAATTGATATAGACTATACATGTANCTTTAANCTGTTGNATGTCCTNCTCNTCTNTTTCAGANNTAAGATATACATGTANNCCTGTCCTTAATACAGAAGTGTCCTCNTTNTTCANGAGGTACTTTGTATACATGTAGGCTTGTCCTCATTATAAAGGGGTCCTCATTATAGAGGTGTTATAAGGTGTCCTGAGATTAGTATTAATAATAAAGTATGTGACAATGTTAGCAGCTTTGATATGACAAGGTATTTCCTAATTCTGTGTACAAGTTTAATTTGAAATGTCACTAATGGCCTCTCACACTTTCTCTCTCTCTCTCTCTCTTTCTCTCTTTGTTTCTAGGTAACAGAGCCTCTACTAGATCAGATGATCATCCTTACCTAAGGCCTAATCCATTTATGGGTCATTTGTCACCAGCTGGAGCGGGTCAGGTCCCTCCATTAATTAAAGACACAAGGTGTCCTCCATCTGGCAGTATAGACCTTGAAGGTGCCATGGCAACCGATCCTTCTTGTAGACCACCTAGTATGTCTGATGTCTTCCCAGCTGGTGAGTATAGGATCATCTCTCTCTCTCTCTCTCTCTCTCTCTCTCTCTCTCTCTCTCTCTNTTANNANANNCTCNTCTCNNNNTCTNTNTNTGNCANTCTGTNTCTACNTGTATCNCACTNTANAATTTNAATCATNTGACATACAGTNACAAGTAATNTTGCTGNCTNGTTNCTATGTACNATTCTCTTACAATTNAAGTGATCTANANNNNCATNCTGTACTNAANTCTTNCTACTCTAAATATTGCTCTTTGNAGTCCNATGNAAATATTGTNATCATTGCNTTNCCCTGTCGNTNTTANTNCTNCCNTCNNTTTCTCTCCTACTTATTAATTTATTTTTGGTATTTTTAGAGTTTCTTTGTTATACCTTATAAGGAACCAGTCAAACTGGTTTTAATTAAACAGGCTTTGAACATAATCTACATCTTGTACGTGTACATTTAGCAGCTTTAGCGTCCATTAATATTATACAGACTAGCCTCTATATAATGGACACTTTGAAAGTGTCTCTGTTATACAGAGGTGTCCTTTATGTGGAGGTTATTTTATACAGNAAGGAATGTAATTGGTACACTAGGTGCTGTCCGTNAAATGGAGGTGTACGCTATAGAGAGTGTCCATTAAGAGAGCTAGGCTATACTAGTCTTGTAGTGAGTGTGTGTGATTTACTATGCTGCCCTCTTGTTATTGTGAGTATTTGACTGTGTCATGGAGTCTGTCCAATTTATGTTAACAAAAGGTTTTGTTTTGAGTGGGCGGGGCACTATACACTAGTCCATTAATATATTGCTACCATAAGAGTGTGTACATGTACATGTACTCTCCAGGTGTACATGTACATGTAAGTGCCCGCCCTTTTCTAATGATAATCGCTAATGCAGTTTGTATAACTACTAGTGAAAGACTAGAGCTACNGGTACATTATCTTTATCCTACTGGTTTACATGTTCATGAAGATAATCATCATNAGGTCACTTACATCTGCTGTACTCTCTCTCTCTCTGTTTCTTTTCTCATATTTATTCTTCTTGTTCTAAATTATCCTCTTTTACTCCTTCCCTTTTACTAAAGGAAGCCATTCTCTCTCCCTCCCTCTCTCTCTCACTCCCCCATAAAGTGAAGCCATCATGAATGCATTAATATACAGTACAGTACTACTGATACCTACTCCATACATTATGGAGGGCCAGCCCTAGTCCTTTAAAAACTACTTAGAGGGCTGGCCTGTGGTTTTGGGTTGACACTCTGAGGTTTTGATCTAGCTCTTCCTCCTCAGGCTCTGTACTGTACCTACACTCCTTCACTAATACTAGCAGCTAGTGTACTGTGTACTACTAACAGACCCTAGTGTACTGTGTACATTGAGCCATGGCTTCTTTTAGTACTGGCATTATATGATTATAATGCCTGTAGAGTGACTTATACCTTTTAATGGTTTCATATTATTGTACTGGAGGCAGTTAGACACTAAATTGTAATCTTGTTAAGTTAAGCCCTTGAACCACTGTGTCTTAGCATGAGGTTTTAGCCTCTCTTAATGGACACTCTCTATAGCATACACCTCCATTTAACGGACAGCACCTAGTGTACCAATTACATTCCTTACTGTATAAAATAACCTCCACATGAAGGACACCTCTGTATAACAGAGACACAGTGTCCATTATAGCAAGGCTAGTCTGTACATGTACATGTATACATGTTAGATTGTGTATGGTTAGTGTGAGACTGTTAGTGGAATATTTGTTTATTTATTAATTATATTTATTAGTTTATAATTCACAAGTGTATTTATCCATTATTAACAGTATCATCAACAATACACCAGCTCAATAAACACCTTCACTGTACATGTACATGTACATGTATAGGGGAAGAGCCTATGACAGTGTGTGTGTTCTTATTATTTATTATTGTAATGTGATACATATAACATATAACATATTGTGGGGTCCCTACCAAACACTACTGTAATGTTAATACCCATTACTAAGTACTGGTGTATACCTGGTACACTGCATTAGGGACATCTTTATAATGAGGACATTTTGTAATGTCCCAATTGGTACAAGCCTGTACAAAAGTACCTCTGAAATGAGGACACCTCTGTATTAAGGACAGGTAGTGTGGGCCCAATGGTGTCCTTAATAAAGAGGCTACACTGTACATGTATTAAGGACAGGTAGTGTGGGCCCAATGGTGTCCTTAATAAAGAGGCTACACTGTACATGTATTAAGGACAGGTAGTGTGGGCCCAATGGTGTCCTTAATAAAGAGGCTACACTGTACATGTATTAAGGACAGGTAGTGTGGGCCCAATGGTGTCCTTAATAAAGAGGCTACACTGTACATGTATTAAGGACAGGTAGTGTGGGCCCAATGGTGTCCTTAATAAAGAGGCTACACTGTACATGTGTATATTATTATTATTGGTCCTGGGGGAGTAACTTGATACATTTATTCACAAACTAATTGTATTCATTCTGTCCAGTAGCAGCTGTCCCTATTTCCCCCTCACNTCTTTCTTTGATGTATTCAAGTGGTTTCATTTTCTCTCTCTTTGTCTTCTCTAGCTTTTCAAACTAACGCTGACGTCCCTCTCCCAGCCTGGCCTCCATCATCTCCTCACATACCAGNGTAACANTTATCAATAATTATTAATAATAATGGAAGAGCTCATGATTTCTACGTACATGTAACATGTATCAGAAAATGTAATNAAAATTGAGAATTTTTACCTTTTCATTTCTGGTGAAATTTGGCCAAAGTCCCTGCGATTTTTGAGCNAACTTTTAACACTTGATATCAGTATCTTAATAGACACTCACTGAACTACATGTACANACACATGTACATATTCATATTCTACCTGNNTATATATANNCCGTATTACAGGTACATGTTCCTTTTTTAGGTATAAGGTCTCCGGTGTGGCAGTATCCAGGTGTCATTACCAGTCAGTCCCTCATTCCCTCTCAATTGGACACTTCAAGTACCAACTCAGTCCCCCAAACCAGTGCCGATAGTTTAAGCAATGGTAGCTCGACTCCGCCCAATGTCACTCAGAATGGTGACCACGCCCAGATTAATAACAGCACCGGGAATGTTAACGACTCCAAATTCCTATTCCCGTCAGGCAGTGCCATCCCACTTTCCCCTGGACTGTTCAACGCTCAGTCGTTCTTTAATCCAGGAGGAAGTAACAATATCCCTATAACACCAACACTACTAGCTCCTTCTGTCTCCTTCTCAGAATCCTACATTAGACCAGGTCAAATCTACTCTCCTTTCTCGTTCACGCCCCACGGCAGTCTCCATGGCAACCTCCCCCGTACCCCCACCCTCCCCCCTCCCTCTCCTCATGCCATTGTTAGTTGCTCCTCTTTCCCTGCTCTGACGGCCATTGCCCACCCCTCCTTCTCCACTAGTAGTCTTCAGTTTCAAAAAGGCGGTTCCTTCCTTGACGACATTACTAAGATCGGCTCCATATCTCCATTCATAGTCTCTCCCTCTCTCTCTCCTAATCGACGTCCAAATGGCACCACTATATTCTTTCCGACGACCATCACAGCTCAAGGTGAAGCTAAGTTCGCTACCATAGGAGAGGTGGGTATGGCCGGGTCTGTGGGCGGAGTCGAGAGGTTATCTGGTACGCCAGACGATGGTAGCATCCATTCTACCGATTCACCGTTGATCAAGAGAGAAGTGTCGTCACCACAACATTGTTACATAGACCAGGAGGCAGCCTAA

>Amq genomic contig with Supt3h, Runx, and DNApolδ (assembled from traces)

TCCTAAGTGATTCTCTTCATTTATAGTCAATCAAGACACAGGTGATATCACTGTTTGTTTATCAAAAGATCTTTGATGCTNTAANGAATTATTTAATAAATTACTGGACTCTCTCATTCATAAAGNAANNGGATGTACGAGTTCATAGCATCAAAAGAACTTGACAAAGAANGATCTGATATTGAAACTGAACNCAGTGATCAATCACATTCACCNCCCACACACACAAAGCCACTTACAACACAANGCCAATCAATAAGANGTTTTACATGAATGGAGATNATAAATATCATTTTNAATGTCCATTTTGCANGCACTGTGACAGAACACAATNTCCTTTTAATAAATGCACAGTACANTAATACNGTTCTATNTACACTGTGCTAGCTGTTTGTATTGACTGATATTGTGAAATTNTTGGAATAAANAACAACTACTTTTTANCACAGACAAATGTTATAATAGATGATGATNATAATAATAATAATAATATTAAACAGAGCGGTTTCTAAACTTTTGCTTCATAAAATGACTCTAAAAATAACAATTAATTAATTTATTAATACTATTACCGCCATACTAACGGTAAAAGGTAGGTGTGGTCCAGTCATACTGCACATGTATCTTCGTATGGCTTCATGTACATGTTCCGGTTGTATGGCAGTGGGCGGGACAGTGAGAGGCGGGGCAACTGGGGGCGGAGTTTGGAGAGGAGGTTCAGTAACTGATGACTATACAAAATAAATNNANACNTATTGTGAATTATTAATAAAACCAAGTGAACCTTTGATTGTCTACTTCTCTTCCTTGATCTTGATTTTGTAATTATGTGAGTGGGCGACGTCATTGTCCCTNNANTNATNNTAATAATAATAATAATAATAATAATAATAATAACTAGAAGAATGTAAAACATTTAAAGGGGCTCAGCCCCCGAGCCATGCTATTGCTGTGCTGTATGACCTTCCTTGCTGCTTGAGAAGTCTTTGGAGAGCAGTTTAGTGTTATATGCAACTTGGTGACCGTTTGCTGCAAATGAGCACAAAGTTGCTGATAGCTGCCATTTTATCATTGTGACCACACCCACTTTCATTGAAGATTAGGCTATTTAACTTTAATAATAGTTAGACGACCCTATCATGCCCATACAGACAGACAGATGTTTTCTCTTCTTAGTAGGACTCACCCAATAATTATAATTACACGTATAACATCAATACATGTACATGTTTGTTTAATGTTTGGTGTCCTCATTATAAAGGTTTCCTTACCTGGTGAGTTTTTAGGTGATCCTGGGGGTGTGGCTTCACCAGTGGGTGGGGTCAGGAATGACGGTGTATTTTCATACGATAGTACTTCTTGAGGTAGTGAAATGGGAGTAGTTGAAGGGGCAGTGCTACCGGTAGGATCAACAATGTAAGATTTTTGTTCTTGCTTGACAATCAGTGACATCTCAACTAACTATGTAACACAACGATACTATAAATAGTAGCATTGCTTCAAATAGAAACGATATACTATAAATAGTAATAGTGCTTAAAACAGAAATGATATGCTATAAATAATAGCAGTGCTTCAAATAGAAATGATATACTATAAATAGTAACAGTGCTTTAAATANAAATGATATACTATAAATAGTAACAGTGCTTTAAATAGAAANGATATACTATAAATAGTAACAGTGCTTCAAATAAAAATGATATACTATAAATAGTAACAGTGCTTTAAATAGAAACAATATACTATAAATAGTAACAGTGCTTTAAATAGAAACGATATACTATAAATAGTAACAGTGCTTTAAATAGAAACAATATACTATAAATAGTAACAGTGCTTTAAATAGAAACGATATACTATAAATAGTAACAGTGCTTTAAGTAGAAACAATATACTATAAATAGTAACAGTGCTTTAAATAGAAACGATATACTATAAATAGTAACAGTGCTTTAAATAGAAAAGATACTTACACTTTGCACTGATCTGTAGGCTATGTAAGCTAATAACTCAATGACAATCTCATGAACTTTAACATCAGCAACCAATGACTGGAGACACAACCAATCTTTGAACTTTTTCTCTAAAGGTAGGAGGAGCTAGTTATTAATGTGGGCGAAGCTAGTTATTAATACATTAATTGAGCCGGTTGACGTACTGTTAACAAATGATGCTCTCCTTGACTGAGTAAAGTCATTGTACTGGGACAAGCTCATTGACTTGGTGTGTTCATCCAACTTCTAATTAATGGATCCACATGCAATAGTAATAATAATAATAATAATAATAATAATAATAATAGTAATAATAAGTTGTGAAAAATACCAAAGCCATTTTTTGGAAAAAATCATGCATGATTTTTTCCAAAAGAGTGGCTCCGAAATCACCCCAAATTTTTATCACATTTTCTGATACATATTGATGTCCTTATGCCGTGTAGAAAGTNTNTGAGTCGATTCCGACAANANAAATTTTTAAGTTACAGCTATTTTAAAAAATCGGCCAATTTTTGAAAAATACCCAAGGCTATAGCCCATGGTTTTTTCCAAAAAGTGGCTCCGAAATCACCCCAAATTTTTATCACATTTTCTAATACATATTGATGTCCTTATGCTGTGTAGAAAGTTTGAGTCGATTCCAACAAAAAATTTTCAAGTTACGGCCATTTTAAGAAATTAGCAATACATTAGAGAATAAACTACATGTAGCTGACCTGCTGATGTTCAATGTCATAATCTTTGTATTGCAATAATTCTACAAATAAACAAACAAATAATTAATCACTGAAGGACAGGTAGTGTGGACCCACAGGCTTGTACCAATTGGGACTTTACAACTACATGTACATGAATATCCCTAAGTGTGTGTGTGTGTGTGTGTGTGTGTGTGTGCGTGTGTGTGTGTGTGTGTGTGCGTGTGTGTGTGTGTGTGTGTGTGTGTGTGTACATTGTATTGCTTCCTCCATATTACCAGCCCATTATTATTATAATTATTATTCTGTTAAAGGTTTCCTATACCCACCAGGTGTCCACCAATAACCACCGCACACACACACCATACCCAGTACAGCCAGTACTACAGTGTTCATCTACAGTATTATGTACAGTATTCATGTACAGTATTAATGTACAGTATTAATGTTNNNGTATTAATGTACAGTATTAAGTGACTGGTGTATTATCCAGTGGTTTCATGTTCCATTAGGTGTACATTACATGTTGCTAAGGTTCCTGTCTCTCCATTGGATACATGTCCTGATGTGTACATGTACTTCCCCTCAGCTCAATTTCCTATTCTACCACCACATGTCTCTATGTGTACATTGTACTGTCTGTGTGTGTGTGTGTCTGGCTAGACGGACTGTATATAAGGAACAGCTGGTCTGTTTAATAATTAATTATTGGTTTAAGATTCCCAACCCATTGCCAATGAAGACACCTGCTCACTGACCATCACATGAGACACAATAATAATAATAATAATGATAATAATAATAATAATAATGATAATAATAATAATAATAATGATAATAATAATAGAAAATGATAATAATAATAATGATGAAATATAAAATTACATGTACATGTACATGTATACATAACGGTTTAATATTCCATTGGTTCAGTACATTAAATTTAATGCAATTCTATTCCAACAAGTACTCATGCAGTAGAGTCTTGATACCTCACATACATGTACATGTTCATGTACTGGACATGTGCTGTACTATACTGTCCACTTCACTCAGTCTCCAGTCCAGACAACACCAGTATACTTATATACATGTACCAGTACTGTACTGTACTGTACACTTACCATCTTGTTCCTTTTCTGTTAGGAAGTCAAGGGAGGCTCTCAGTGCCCTATAAGAAGAAACAGCTTTAGCATTGGGTTCTGAAGAGAGAGAGAGAGGGAGAGAGAGAGAGAGGGAGGGAGGGAGAGAGGGAGAGAGAGAAGGAGGGAGAGAGAGAGGGAGGGAGAGAGAGGGGGGAGGGAGGGAAGGAGGGAGAGGGAGGAGAAAGCTCATTCAAGCATAACGTACAGTATATTTTCTACCTTGTGTCTCATCCTCAAGGTCTTCTTCATCTGGTAAGTTGCTGATCTGTGAAAAAGCTTTCCTCTTTTTCAAATAATTGACAGCTGCTTGGAGCTTTTGCTAAAAAAGAAAACAAAACAATAAGAAATAAATAACAACAACAACAACAACAACAATAATAATAATAATACAGTATAGCGGAAAATTTTCATGGCCCAAAAATTTTGCAGACTTGTTCAAAAATCAATTTCGTGGCTTACTTTTCGGCCATCTTGAACTGTATTAAATCATTTTCAAGGATTTTATTTTCATGGATTTTGAAAAACGCAAAATCCGCGAAAATTTTGGGCCATGAAAATTTTCTGCTATACAGTAATAACATCATTATTACTTCTCTCTCCCTCACCTTGTCTCTCCTCATTATAAACAATATGTCTGACAAGAGGACACTCTTTCCATTCTTCCTAGACACCATCTTCACAGAATCACTGATCTAATGGATACATGAACAAAATTAACATATAATTACATGCAGCTGCATCATTCCCTCTCTCTCCCTCTTCTTCCCCCCTCTCTCTCTCTTTCCCTCCCTCACCAGTTCAGTTATTTCTTGATATATTATTTCCTCAACGAGTTGAACTGACTCTACATTAGACTGCTTAATGTCACCAAACGCATGCCTAACAATTAATAATAATAATAATAATATTAAAAGCGAATTCATTTATAACTAATAATTAACTAGTTTTCAATTTTATTTTTCTTCTATCACTAAATTAGAGTCGATCGTTACAAATAGATATTACATATAAAGTTACATGTACATGTACATGATGATGCTAAATGATGTAAAATGATGTCACTACTATCTAATGATATACCAGAACTAGAGATGTGGACGTATACATGAAGATGTACTTAATTAATAGTTAATAATAATAATTATTGTTGTTATTGTAATGATATTATGTTTTAAATTCAATTATCATAACATNATATTAACAATATTATTAATCTTTATTGATTTAGCAAATCATGAAACATTACCATGTGAAAGATTCACTTCACAAACCAACAGCTGCAGAACAGACGATAATACACATGTACATGTGTTCATGAAATGTATTATTATTATAATTAGATTATAGTAAAATTAATTGAATTAGTGAACCACTCTCTTGAGATATTGACAGATAATGCAATGATGGGATAGTTCAGACCACGCCCCTCACCCCACCCACCACTTACATCATACCAGATATCTCAGAATGAAACATGTTGACATACTACACTTGTAAAATAGTGTAAACTAGAAGGAACTAGGGAGGGAGGGAGGGAGGGAGGGAGGGAGGGAGGGAGGGAGGGAGGGAGGGAGAGAGGGAGAGAGAGAGAGAGAGGGAGAGAGAGATTAGTAATCATATCAGTACATGTAGGTACATTATACAGTGAAGTGGGTGTATTTCGTCTGGAAAGTGCTCTTTCTAAACAAGATTATTAGGATTAGATATTTTAGTTTGCTTTGCTTTTGAGCAGCCATTTTGTACAGGTGAATAACTGTTTTCATTTGGAAAAAAACATCATGTATTAGTTTCGCAATGAGAACAAAAATAACACAATTAAACACTGACGGAATACACCTCCTATACAGTACATAACAATAATTCTTCATTCTAAGTGTAGGGATAATTAGCTTGACAAGTGGTCTGATTATCGTAAAAAGAATAAACTAGCTTACACCTGGCAATACACTACTAATTGAGGACACTCAATAATTTGTATAAGCACAGGCTGAAGCTGCCATGGGAGAATAACAAAGCTGAGAAAGAATTTAAGGAATTTAATTATTAAAAATGAGTAAGGCAACTAACAGGACAGTACGGGGATGTGTGGTCTGCGTTAGAAAGAAAAGGAGACAAATAAGAACCTAATTGTGAGAAAAATAGAGAGAGAAAATGCATTTTTAAGTTTCATTTTCACTTAAAACATCTCAAAAACTTACAAGAAAATTTCGAGAAAAGAATAAAATGGCGGGCGATGCCTCACGTGATCCACTAATTAACTAATCAGAGGTCACGTGATATATATGAATATAATTAAAATACGTGAAGCCAAAAAGAATAAAAAATGAGTTCGTCGGCGAGTGAAATGATGTGATTCTTTAATACAGGACTACTGCAGTACAATATAGTGTGTGTGTGTATATATCTTCTCTCCTGCTCTTCTAAGGCTCCTCTGGTCCTTTCTCTTTTACTAAACGACTACTGAAAACTCTTAGATTCTCTTCATTTCTCTCTCTCTCCGTCCACTCCCCCTCTCCTCCTCTCCCTCGACTACAATTCACTGAACAACGTGGANTATTATAAGAACCAAAGGCGCCATCATTCTAAGACCAGCTAAGCCCAGAGCTCGTTTACAACCTACAACTGATACAACAGCAACTCAACACTCACTCTAAACCTTTCCAAGGTAAGCGTGGAGCAGGATAGAGCATAATGTANTGTATTTCTTTGCTCACTCGCTAGCTTCACAAANTCACCTCAACCTCCGTCTCTCAGTCTTCNAGAAGATCCCCCTTCTCCCACAATAGCNATAACTCTAACTTCATTTACTAAAGGTAAGGATATTCTTATACATGATTTACTTAAACTCTCTCTCTCTCTCATGTTAGATAATAATCATTCATCATACACTAGTAATACACTAATGCCTTTAATCATGTCTTCTGAATCAATCCCACCTCCTGATGGTCCCCTACCCCCTCCCTCCTCCAAACGCTATCGAGGAGAAAGAACCTTCTCCGAACTATTAGCGGAATACCCTGGAGAACTCGTAACGACCGATAGTCCTAACTTTGTATGTACTATCCTTCCGTCCCACTGGAGATGTAATAAGACCTTGCCCGTACCGTTTAAAGTACTATCGCTCTCCGATATCACTGATGGTACCAAGGTGATACTAACAGCCGGTAACGATGAGAACTCAGCCGCTGAGCTTAGGAACGCCATAGCAACTTTTAAGAACCAAGTGGCTCGGTTTAATGATCTTCGCTTTGTCGGTCGATCGGGAAGAGGTGAGAGGGTGAGGGATGTAGACCATCATGTATTATTTTGTGTTAAGTGGCCTATCATTCATAACCCATTATTGCTTTGACTAGTTGTAATGGGCAGCAATAGATAACTGGCCATTAACATAAGAGGCCATTAAACCACTTACTGTCTGCCCTTTTATTTGAACAAGTTTATAAGCATAATGTACAGTCTAGCTTCTATAATAAGGACACCTTTATAATGGGGACATTTTCTGATGTCCCAATTGGTACAAACCTATACAAAAGTACCTCTGAAATGAGGACACCTCTGTATTAAGGACAGGTAGTGTGGGCCCAATGGTGTCCTTAATAAAGAGGCTACACTGTACATGTATTAAGGACAGGTAGTGTGGGCCCAATGGTGTCCTTAATANAGANGCTACACTNTANATGTANTAAGNACANGTNGTNTGNGNCCAATGTTNACAATATATAAAGTAGTGTTCACACACATTGATTCACACAATAATATTGTAATTNNCTATCATTAATTTATTAATTTTAATATTATAATTACAACATTAAAGCATGTAAGAATTAATAACGAAATAAAAAAATGAAAATTATAATAAATTTATTTTCTATTTAGGTAAAATGCTGACAGTAACAATCACCATAGTAACAGAGCCGGTACAATACGCTACTTACTCTCACGCCATTAAAGTGACAGTGGATGGACCAAGAGAACCAAGACGTAAGACACTGAGAGAGAGAGGGAGAGAGAGGGGGGGGGGGAGAGAGAGGGGGGATGTTAGGTGTATATACATTTTATGTACATGTAATGTGCAGTTGGTAATTTTTTGGTAAGATTAGCGGAAATTTTGGTCGTTGGCAGGTTCTTTTAATTACATGTACATGTACATGTATATATTTAATTTGGTGATTTTTGATGTCTGCATTACTTAATGTCTGTTACTGATTGTCTGCTTTTAAATATTGGAAAGAGATATACATGTACATCTAAAGACTTGAAATCAACAATCGTTCAATGTATATCCTCTACAATGTACATGTACTGTAGCTTATCCTTTACAAGTTCATGTACTGGACTCAGAGGAATTAAGATAATGATACATGAACTGTATGTGTGATCATCATTGATGTACTGTTCTGTATTGTTAGGTGAGATCAGGATGTACTGTGTTCCAATTGTCACCATTATATCATTATCATAATAATGAAATCTTTTGTACTTGTCTCTCTCCCCCTCCCTCCCTCCCTCTCTTGCACACATGAAATGTAAAATGCTTTCATGTTTAATGACCTGTGACTCAAATTGTAAAGAATGACTTTAAATAGCAACACACACACACACACACATGTGTACATGTACAATGTATAATGTATCCTATTGATAGAAAGTGTTTATTAATATTGTAATGTTTATTAAACAACACATTGCTTTATAATGGGAGACAAATCACAGCTACTACAACTAACAGTACATGTAGTGGGGTAGGGCAGGCCTAGTCTACTAGTTCTTTTATACTAATGGTACACTATCACAAGTCCTACATTCAATTCTTGTAGACTGTACGTGGACTTTAAACTGATGGTACACTATCACAAGTCTACATTCAATTGATATAGACTATACATGTANCTTTAANCTGTTGNATGTCCTNCTCNTCTNTTTCAGANNTAAGATATACATGTANNCCTGTCCTTAATACAGAAGTGTCCTCNTTNTTCANGAGGTACTTTGTATACATGTAGGCTTGTCCTCATTATAAAGGGGTCCTCATTATAGAGGTGTTATAAGGTGTCCTGAGATTAGTATTAATAATAAAGTATGTGACAATGTTAGCAGCTTTGATATGACAAGGTATTTCCTAATTCTGTGTACAAGTTTAATTTGAAATGTCACTAATGGCCTCTCACACTTTCTCTCTCTCTCTCTCTCTTTCTCTCTTTGTTTCTAGGTAACAGAGCCTCTACTAGATCAGATGATCATCCTTACCTAAGGCCTAATCCATTTATGGGTCATTTGTCACCAGCTGGAGCGGGTCAGGTCCCTCCATTAATTAAAGACACAAGGTGTCCTCCATCTGGCAGTATAGACCTTGAAGGTGCCATGGCAACCGATCCTTCTTGTAGACCACCTAGTATGTCTGATGTCTTCCCAGCTGGTGAGTATAGGATCATCTCTCTCTCTCTCTCTCTCTCTCTCTCTCTCTCTCTCTCTCTCTNTTANNANANNCTCNTCTCNNNNTCTNTNTNTGNCANTCTGTNTCTACNTGTATCNCACTNTANAATTTNAATCATNTGACATACAGTNACAAGTAATNTTGCTGNCTNGTTNCTATGTACNATTCTCTTACAATTNAAGTGATCTANANNNNCATNCTGTACTNAANTCTTNCTACTCTAAATATTGCTCTTTGNAGTCCNATGNAAATATTGTNATCATTGCNTTNCCCTGTCGNTNTTANTNCTNCCNTCNNTTTCTCTCCTACTTATTAATTTATTTTTGGTATTTTTAGAGTTTCTTTGTTATACCTTATAAGGAACCAGTCAAACTGGTTTTAATTAAACAGGCTTTGAACATAATCTACATCTTGTACGTGTACATTTAGCAGCTTTAGCGTCCATTAATATTATACAGACTAGCCTCTATATAATGGACACTTTGAAAGTGTCTCTGTTATACAGAGGTGTCCTTTATGTGGAGGTTATTTTATACAGNAAGGAATGTAATTGGTACACTAGGTGCTGTCCGTNAAATGGAGGTGTACGCTATAGAGAGTGTCCATTAAGAGAGCTAGGCTATACTAGTCTTGTAGTGAGTGTGTGTGATTTACTATGCTGCCCTCTTGTTATTGTGAGTATTTGACTGTGTCATGGAGTCTGTCCAATTTATGTTAACAAAAGGTTTTGTTTTGAGTGGGCGGGGCACTATACACTAGTCCATTAATATATTGCTACCATAAGAGTGTGTACATGTACATGTACTCTCCAGGTGTACATGTACATGTAAGTGCCCGCCCTTTTCTAATGATAATCGCTAATGCAGTTTGTATAACTACTAGTGAAAGACTAGAGCTACNGGTACATTATCTTTATCCTACTGGTTTACATGTTCATGAAGATAATCATCATNAGGTCACTTACATCTGCTGTACTCTCTCTCTCTCTGTTTCTTTTCTCATATTTATTCTTCTTGTTCTAAATTATCCTCTTTTACTCCTTCCCTTTTACTAAAGGAAGCCATTCTCTCTCCCTCCCTCTCTCTCTCACTCCCCCATAAAGTGAAGCCATCATGAATGCATTAATATACAGTACAGTACTACTGATACCTACTCCATACATTATGGAGGGCCAGCCCTAGTCCTTTAAAAACTACTTAGAGGGCTGGCCTGTGGTTTTGGGTTGACACTCTGAGGTTTTGATCTAGCTCTTCCTCCTCAGGCTCTGTACTGTACCTACACTCCTTCACTAATACTAGCAGCTAGTGTACTGTGTACTACTAACAGACCCTAGTGTACTGTGTACATTGAGCCATGGCTTCTTTTAGTACTGGCATTATATGATTATAATGCCTGTAGAGTGACTTATACCTTTTAATGGTTTCATATTATTGTACTGGAGGCAGTTAGACACTAAATTGTAATCTTGTTAAGTTAAGCCCTTGAACCACTGTGTCTTAGCATGAGGTTTTAGCCTCTCTTAATGGACACTCTCTATAGCATACACCTCCATTTAACGGACAGCACCTAGTGTACCAATTACATTCCTTACTGTATAAAATAACCTCCACATGAAGGACACCTCTGTATAACAGAGACACAGTGTCCATTATAGCAAGGCTAGTCTGTACATGTACATGTATACATGTTAGATTGTGTATGGTTAGTGTGAGACTGTTAGTGGAATATTTGTTTATTTATTAATTATATTTATTAGTTTATAATTCACAAGTGTATTTATCCATTATTAACAGTATCATCAACAATACACCAGCTCAATAAACACCTTCACTGTACATGTACATGTACATGTATAGGGGAAGAGCCTATGACAGTGTGTGTGTTCTTATTATTTATTATTGTAATGTGATACATATAACATATAACATATTGTGGGGTCCCTACCAAACACTACTGTAATGTTAATACCCATTACTAAGTACTGGTGTATACCTGGTACACTGCATTAGGGACATCTTTATAATGAGGACATTTTGTAATGTCCCAATTGGTACAAGCCTGTACAAAAGTACCTCTGAAATGAGGACACCTCTGTATTAAGGACAGGTAGTGTGGGCCCAATGGTGTCCTTAATAAAGAGGCTACACTGTACATGTATTAAGGACAGGTAGTGTGGGCCCAATGGTGTCCTTAATAAAGAGGCTACACTGTACATGTATTAAGGACAGGTAGTGTGGGCCCAATGGTGTCCTTAATAAAGAGGCTACACTGTACATGTATTAAGGACAGGTAGTGTGGGCCCAATGGTGTCCTTAATAAAGAGGCTACACTGTACATGTATTAAGGACAGGTAGTGTGGGCCCAATGGTGTCCTTAATAAAGAGGCTACACTGTACATGTGTATATTATTATTATTGGTCCTGGGGGAGTAACTTGATACATTTATTCACAAACTAATTGTATTCATTCTGTCCAGTAGCAGCTGTCCCTATTTCCCCCTCACNTCTTTCTTTGATGTATTCAAGTGGTTTCATTTTCTCTCTCTTTGTCTTCTCTAGCTTTTCAAACTAACGCTGACGTCCCTCTCCCAGCCTGGCCTCCATCATCTCCTCACATACCAGNGTAACANTTATCAATAATTATTAATAATAATGGAAGAGCTCATGATTTCTACGTACATGTAACATGTATCAGAAAATGTAATNAAAATTGAGAATTTTTACCTTTTCATTTCTGGTGAAATTTGGCCAAAGTCCCTGCGATTTTTGAGCNAACTTTTAACACTTGATATCAGTATCTTAATAGACACTCACTGAACTACATGTACANACACATGTACATATTCATATTCTACCTGNNTATATATANNCCGTATTACAGGTACATGTTCCTTTTTTAGGTATAAGGTCTCCGGTGTGGCAGTATCCAGGTGTCATTACCAGTCAGTCCCTCATTCCCTCTCAATTGGACACTTCAAGTACCAACTCAGTCCCCCAAACCAGTGCCGATAGTTTAAGCAATGGTAGCTCGACTCCGCCCAATGTCACTCAGAATGGTGACCACGCCCAGATTAATAACAGCACCGGGAATGTTAACGACTCCAAATTCCTATTCCCGTCAGGCAGTGCCATCCCACTTTCCCCTGGACTGTTCAACGCTCAGTCGTTCTTTAATCCAGGAGGAAGTAACAATATCCCTATAACACCAACACTACTAGCTCCTTCTGTCTCCTTCTCAGAATCCTACATTAGACCAGGTCAAATCTACTCTCCTTTCTCGTTCACGCCCCACGGCAGTCTCCATGGCAACCTCCCCCGTACCCCCACCCTCCCCCCTCCCTCTCCTCATGCCATTGTTAGTTGCTCCTCTTTCCCTGCTCTGACGGCCATTGCCCACCCCTCCTTCTCCACTAGTAGTCTTCAGTTTCAAAAAGGCGGTTCCTTCCTTGACGACATTACTAAGATCGGCTCCATATCTCCATTCATAGTCTCTCCCTCTCTCTCTCCTAATCGACGTCCAAATGGCACCACTATATTCTTTCCGACGACCATCACAGCTCAAGGTGAAGCTAAGTTCGCTACCATAGGAGAGGTGGGTATGGCCGGGTCTGTGGGCGGAGTCGAGAGGTTATCTGGTACGCCAGACGATGGTAGCATCCATTCTACCGATTCACCGTTGATCAAGAGAGAAGTGTCGTCACCACAACATTGTTACATAGACCAGGAGGCAGCCTAAGGAGGAGGAGAAATAATAATAATAATAATAATAATGACGATAATTTAATGATTATAAAGTGAGTTTTGAGTTTGTAGTAAATATATTGTGTCAATAATAATAATGAAATCTTTTNGTTTTTATTGCATTAAGCTTGATTTAATTGTACACGTACACGTACATTTAGCTCGTACACATGTCCAGTATATACCTTATATTAGATATATGGAGCAACACTGTGTGTGTGTGTGTTGTTAGTACAGTATATGTAGTGTGTGTATTATTAGTAGTAATACTGGGACCAGGTACTATTAGTATACTAGTGTCTTAAAATGGCTTCTAGTTACTACAGGACGCTGATGACCCAGTACTATACTTGTCAATCTTTGTGTCATTGTGTTTGTGTTTTGTAGTCTAGAGAGAGAGAGAGAGAGGATCAGGGGATTGTGTTCACACTCAGTATTATTATTATTATTTGTGTTGTTGGTTCTATTTGTGGTCTGNGTATCTANCGAAGTATCCAGCTGCTNCAGTGTGGNTTGNCTCTTCTTGTTAANTAATATAANACCTTTAGTGAATGNNTTCTCATCATTAATAATGACATCTCTCTCTCTCTCTCTCTCTYTAMTSWYTMTAAGATTACAATTCTTTCTCTTCATTCATTTCATATTTCTGACTGGCACCACAACCCCACACAATCACTCTCCATCCTACCACCACTCACAGGATAATTTCATGTCTCTGCTTCCCCTCTCTCCCTCCCTCCCTCCCTCCCTCCCTCTCTCTTATAAAGTCACTTGTTATCATTATTATTATTATTATTATTATTATTATTGTTGTTGTTGTTATTTTTTCATTCAAACTTTCTTTCTACAATCAGTGACATTCTTTTTTGTATAGTTTATTTTATTTTTGTATCTTCAATCTAGTAAATTTTGACAATCTCTCTCTCTCTCCCTCTTTCTCTCTCTCTTTTGTAATATATTAATAATTATTATTCTGATGTCATGACACCAATCTCTTTCTGTCTCTTGTGTTTGTTTATTATCATTATTATTATTAATATTATTATTATTTCAAACTTGATGAGTTTTCCATCTCTTTTCTCTCGTTTATAATATTATTATTTTCCAATCTGCTCAAAATAATGGAAATTAAACTGGCCAAACTTYCAAGCCACACTCTTCTCTCCCTCTCTCTCTCAGTTCAAAACTGTTTTTGTAACTATTGTAATTATTTCCTTGTTAATGATAATAATTAATATTAAAWMAAWRMWMAAAMWWWMWAMAWAAMATAAGGACAACAATATGTATCAGAAAAATGTAATAAAAATCTGGGGTGATTTTGGAGCCATTTTTCAAAAAACCATGGGCTATAGCCTTGGGTATTAAAAAAAATTTTGCATTTTCTTAAAGTGACTGTAACTTAAAGATTTCTTATCAGAATCAGCTCAAACTTTCTACACAACATAAGAACATGTATATGTATCAGGAAATGTAATAAAAGTATTCCTATAGTGTGTGTCTGGTCCCAGTGGAGCAATGCAATACTGAGAGGGTTAGGTAATGTAAAGTGTTCCTGAGATCATTGCATGTACATGTATGTGTGTCTGTACATGTATAATCCAATTATTATTATTATTTAGTAAATGATATCACTATATGTTTACTACAAGAAGACATCTGTCACTCACCTGCAGGAATGAGAATACACAAACAGGTGGTATCAGAGATTACTGGTACCAAATATGAGAGGTGGTATGGAAGATTGAGCATGGAACACATGATGCGTAGGAGAAATTTATTAATAATAATGATTATTCTTATCAGATCTTATAATCTAATCTGTTTGATAAAATTCTTGGATATCATTGGACCCACACTACCTGTCCTTAATACATGTACAGTGTAGCCTCTTTATTAAGGACACCATTGGGCCCACACTACCTGTCCTTAATACATGTACAGTGTAGCCTCTTTATTAAGGACACCATTGGACCCACACTACCCGTCCTTAATACATGTACAGTGTAGCCTCTTTATTAAGGACACCATTGGGCCCACACTACTTGTCCTTAATACATGTACAGTGTAGCCTCTTTATTAAGGACACCATTGGACCCACACTACCCGTCCTTAATACATGTACAGTGTAGCCTCTTTATTAAGGACACCATTGGGCCCACACTACCTGTCCTTAATACATGTACAGTGTAGCCTCTTTATTAAGGACACCATTGGGCCCACACTACCTGTCCTTAATACATGTACAGTATAGCCTCTTTATTAAGAACACCATTGGGCCCACACTACCTGTCCTTAATACAGAGGTGTCCTCATTTCAGAGGTACTTTTGTATAGGTTTGTACCAATTGGGACANTTNACNNAATGTCNTCATTATNAAGNTGTCCTCATTATAAAGGTGTATGTCCTACAGCTAGTATTTGATGTGTTACTGCATTCCGGTTGTTATTGTAGGCTGAGATATGAGTCCTTAGAAGTGACCTAGGAGACTTCCTGTCTGCCAGCTAGTGTTCTCAGCAGATCCTTTCTATTCCTGAAAGCCACAGCCTNCCCAGATACATTATTGTGATACTGTAGGACTGTTGTATGGGTTCACTCCATCAGTACAATGTGTATTGTAGAGATTCTCCTTTAATTGAATTATAGGATCATATTATACTAGACTAGTCTAACATCACAGAGTTAGTACACAACTTGTTAGCAAGTATACAAACATACAGCACTGATGCTGTACAAGTATAAGTACATCATTAATACACTGCAGTACATGATGTGTCACAATAGCTGCTGCAATCATTGTGTATCAGTAACTATTGCTTTATGTAAGATGTACATGTACAGACACACACAGTTATAGGAGATTATAACTAAATCCCTGTTAATGATAATAATAGATTGTGTAAGAGAAGCAGTGAGCAGTTACAAGAGGAGACTAGACGCACCACATGTAATGAAACATGTAACATGGAGAGGTAACATGTTCATGTATCATGTTATCTGGTCTGCCGTCAGTCCTTACTTGGTATCTGTTATATGTAGAACTCACCAGTGTGTGCTAATATGAGACCCTGATTGAGTTGATTATTAGGAGGTGCAATTAATGAAAATTGTAGAATCATTAATAGCTAAACTGTTTAAAATGCATGCAAAAATAATTGTGACAATTGGAGGCAAAACATAGCAATACTGTCACCACTATGAGGTGACAATTCATGGTTCCACTTTGCAGTAAAAATACCAATTTTTAGGTGAAATTTGTCCACCAACTAATTTTCCAGGGTTGTTCAAAATGGCTCTAATATTACAATTTTTATTACTATAAATCAAAGTGATTAAAAATAAGATAAATTGACACTAAAAATATAAACAATAATTTATTAAACACCTAAATACAGGAATGGCCTTCCTTCCAATAGTCTCCTATTATTATTATTATTATTATTACTATTAGGATCATTTTTCTTTTTTATGATTCTTAAAAGTCTCTTTGGTATCCTCTTCTTTATTCTTGATAGAGTCGGCCTTCTTCTTAAGTCCGTGGTCTGATCCATAACGAACTTCAGTCATTTTAATAATAAAATAAAAAAGCTGATTAACTAGAGGGAGAGAGAGAGAGATAGAGAGAGCAATAATTTATTTATTAATGAACAACTATACAATACCAAATAAGGAAATACTTAAAATCAACACACACACACACACACACACACAGAGAGTAAGTATAGTACGTTAAATATTTGCGAGTAATTATAATTAATTAATTTATTAAACAGCCACACACACACACACAACCATATATGGCAAAACTTTCATTATTTTAACAAATTTAAATCCAATCTTAAAATCAAAGTGTGTGCATTAATTAGAATAACCAGTACCTATTATTAATGAGCTGACCACACCCACTAGAGTGTACCACGCCCCCACATATAATAAATAAGAGGCCAGAGAGAGGATGGGTAGACCAACGAACAGTATCCAAATGGACATCACGACATTAGGCCAGGGACGGGTAGGGACCTGTATCTCGTGATAAGGAGAAGGAAAGACCTTGNNCTNGATANGANGATCCANCAGGTCATCNTGTGGAGAGATGTGGAATTATCAAACACTTTACAATACAGGACTTTTGTTTTTAAATGGTTATAACTTAAAATTTTTTGTCGGAATCAGCTCAAAGTTTCTACACAGCATAAGGGCATCAATATGTATCAGAAAATGTAATAAAAATTTGGGGTGATTTTGGAGCCATTTTTTGGAAAAAACCATGGGCTATAGCCTTGGGTATTTTTCAAAAATTGGCCGATTTCTTAAAATGGCCGTAACTTGAAAATTTCTTGTCGGAATCAGCTCAAACTTTCCACACAGCATAAGTACATCAATATGTATCAGAAAATGTAACAAAAATTTGGGATGATTTTTTAGCCATTTTTTGAAAAAAACCATGGACTATAGCCTTGGGTATTTTTCAGAATTTTATCATTTTCATAAAATGACTGTAACTTGAAAATTTTTTGTCGGAATCAGCTCAAACTTTCTACACAGCATAAGGACATCAATATGTATCAGAAAATGTAATAAAAATTTGGGGCAATTTTGGAGCCATTTTTTGGAAAAAACCATGGGCTATAGCCTTTTAAAAAGGGAACTGAGCCCTTAAATTAATTATTTTTAAAAGAAGACAAGACAAGAGAAATTAACAAGAAAAGAAAAAAGAAAGTGAAATAATAATATGTAGGAAGACAAAAATTATAAATATGATAATGATTAAGAATTAAAATGTGAGTCTGCCATGGCCCTTGAGAAGTCCGCTGAGTTCACATATCGGACCTTCGACCGTGTACCCCTGAGACATCTAAGTGCCGATCTTAAGATGGAAAATGAATATTGGCATCTCAGAAAGTTCATAGTTGTGTTGAAAGGTCTCTGATGTTTCTCTGATAAAATTAGTGCCAGCCTCTTAATGAATGACGTGGCTACCGGGCCACACCCTCCAGACGTTGAGAATATCAGTGGTGAAAAACTTGCATGTTCTACTTCCCTTAGCCTCTGCTCATATTTTCTCTTCTTCAGTGATTCATTGTCTGTATAGCATTTCTTTAGGGTCTTGCTCATGTTGGTAACCGATAGTGGACAGAATACTCGTACGTCAAAATATACCGTCTTGAACCGTTCTCCCCAAAATCCTTCAGCTGCAATGTCCAACCTGGCCTCATCCTCAGTGGATGCTGTCCTATATCTAAGAGATTCCCCTTTCAGGGGTTGTAAATTAGGTTCTCTTACGACATTTGGGCAAACATCTTGTAGCAAGGCAGCAGACAGATCCCTGATATCATTGTGTCTCTGTGTAACTAGCCCACCATAAAGACATGTCAGAGAGTGCTCGACTGAGAAAGGTGTGCCACATGTACAGGTCAATGGAAGGTCCAACGGGCGCCATCCATATCTGAGACACAAAGCATCCCTGAACTCCCCCTTACTCATATCAAATCCATACTCCTTAAGGGGGAGTGCAGTCAACCATGAGGACGCACCCTTCTCAGACGAGACATCAAGTAGGCGGAGACGGTCAGCCGGTAGCTCAGACCTTAGTGTAGACATTGTTGATCGGTATACTTCCTCCTTTTCCAACTTACATTTCTTCTTTGCTTTATCCATCTCCACCTCAACATCAGTCACAGAACCATTATCATTCCCCAACAACATATCAACAAGAGGCTGAGTGATAGCAACAGAACTTGATCTTTGACACCTAGCAAACCAACATGGATTGACCAGCCCCAAACCACCATATCGAGTAGGAAGAGAGAGCCAGTCCCTCTCCAACTCACTCACAGCATCTCTTCCCAAAAGAGAAGGAATAAAAACATTAAACAAACAGTCATTAAGGGGTTGGACCTGACACTCCTCAAATTGACAAGTACGAAACAGATAAGTCCATTCACTAAACAAACCATGAGTGAAGGCACTATAGGCAGACTGGGGCTGAGAAACAGCAACTTTAGATAACAGACATACTCTCTCACACCATACCTTAACCTTATTAGTCAACTCCTTGTTCACATACTCCTGGGTACCAACAGGACATCCCAGTACCTTAACACCTTCACACGTAATCATCACATCAGTACCGCGAAATAGCTCACGGGCCTCCTCCAAGTGATCTTTCTTTACTAGAAGAGTGGACTTGATGGCATTTGGCCAATAACCATAATAGACTCCTAGCTCCTTAATCCTGCCCCACCACTGTCTCAAACCACGCAGAGAGCCCATACCAGTTGCATCGTCTGCATACCAAAGNTGTCTTACATTAGGAATAGATGACAGTTCCCGAATCAGTGGAATTGATGCAACAGCATACATAACCATAGCCAATGGATCTCCCTGCGTGGTCCCCTCCTTAGATAATAAGGTATCACCACCAATAAAGAGATTACTCTCAATACGGTATAAATTAATCAGAACCCGACCAAGGGAAGGACAAAGATGAAGCACATTCCTCAATGCCATACCTCTGTTCAATGAATTGAAAGCATTAGTCGCATCAACCAATAGAAAGCCCTCAGCACCATCAGAATCATATACTTCACGAATCATNCANNGGCCACNGCCTCCACAAGCAGCACTCATACCAGCACACAATTGAGAGCAACCAGTCACCTCCATGATATCAGATTTAATCACATTAAGAATAGACTTACCGATGACACGCCTAATGACCTCTCCAATTCCAATCGGGCGGGATCCTCTAGAGTCTACCTGCAGCAATGAAGCTGCACTGCCGTCTGCCGCCTTCTGNNNNNCCCCCCCTTTTTTTTTTTTTTTTTTTTTTTTTTTTTTCTTTTNTTTTTTTTTCTCTTTTTTTTTTTTTTTTTTTTTTTTTTTTTTTTTTTTTTT

>OscRunx mRNA (partial contig assembled from ESTs)

AGCAGTGCAAGGGTGAAGTTGGGTTTAGTTTTGCCCACTGAAACCGCCTCGTCGGCATCC

CGCTAATTTTATCTGGTCAAGCCTCCGTCGTGTTGTGGTTTGTTCGCCTCGCCTACTCAA

CGTCGCCAATGCGTCTCATGATGGAAAGAGAGCCGGCCCCTAAACGCAGCAAGGATTCCC

TCGAGCTGTCATCTTCGATGGGAACGTTGTCGGCGTCAGCTTCGGCGGCGGCCGAGCACC

AGGGCGACCTCGTGAAGACTGACAATCCTAACTTTGTTTGTACAATCTTGCCGTCTCACT

GGCGCGTGAACAAGACGCTCCCAGTGCCCTTCAGGGTGCTCGCCGTTGGTGACATCTCCG

TTCCCGACGGCGTCAAGGTCACACTCAAAGCATTCAATGAGGAAACCGTCAGCGGAGAGC

TGCGCAACGCCACGGCCATCTTTCGAAACAACGTTGCCCGATTCAACGACCTTCGATTCG

TTGGTCGGTCCGGTCGAGGCAAATACTTCGACGTGTTGATCACCGTTCAAACGGACACTG

TTCAGAAGGCTATCTACAAGAAGGCGATTAAAGTGACCGTCGACGGACCTCGTGAACCTC

GACGACACAAAGTGAAGGAACGGCAGCTCTTGGCAGCGCATCAGCACCATCACTCACCCT

ATCACGGATATCCGAATCGCCAACATGTCCTCCCGCCAGATTTCATGCCACTCTCGTCAG

CTGCTGCGTCATCTCTGTCGTCGTCGTCCAGCTCTCTCGGATCCGCCGGCTGCGAAACTC

CGCAGCTCCAAAGAGCCATACACCGCGACAGCCTTTCCGCATTCAGCACGATTGCAACAG

AGCCAATCATGCGGCGACCGGCCTTCAGTCAGCCGATGGTGACAATGAACCACGTTCATT

ACACGCAAGAACCGACGACCATGTCGACGCAAATGGAGCAGTCTCATTGTCCGTCAATTC

CTCCATCCATCTCAATGCCGCCCACTTTCACATCCGAAGCGTCGTTGTCGATGTTGGCCG

GGCCAGCCTTCGCTCCCCGGCAATCCGTTCCCGGACACATCGAACAAGGCTTCGTCTTTC

CGCCACCCTTTCCCCTCCGATCGCCTACATCCGCCGCTGGATTCGCATTTCCTGGCGGTC

CTCCCGGCCTCGTGCCTCTCTCACCCATCCCTGGCGATTCAGCCTCTCCCTTCCCTCACC

GCAACTATCGACCTATCGCATGCCAGGTCAGCATTTCCAGTCATGGGAGATCGTCCGGCT

CAGGCTACGACGCGCCGGCACCGGCAATGCCCGGATTAGTCAGCACGTCCGACCTTTTCA

GTCTCCCCGTCACTCCGAGGACTCCCATTACGCCGACGGTCCGTTACGCTCAGATGCACG

CCGCTCAGGCGGCTGGACAACTGATGGCGACGAGCAGCTCCGACGCGCTTGGCTACGGCC

ACTTGACGGCGGCCATCGGTTCGTTTCCGTTCGACCAGTACGCCACTCATTTACAAGACA

TTCAACACATGCAACAGCACAGTGCGTCGATGACGTCACTTAATGGAGAGCCGTCGTCGT

CCGGCGTTACGATTACTCTGTCGCCTCCGCCTAACAAGGCGAAGCCGACGTTGAGTCGGA

GCAGTTCGTTTACGTCTGGAGCCGGAAATGGAAAGATGGATTCGGGAGAGACGGATGAGA

AGGAAGGACTTTGGAGGCCCTATTGATTAATTCAATGTCCTCGTCTCTTTGGAAATTTGA

TTTGGGTTTTTCTTCCAATTTGCAAAAAAATTGGAGGTGGACCTGATGAGACTCGTTTTA

TGATTCTTCTGATTAATATTAGTGCAAGTGTGTACCTTTTTCTTGTCCGCGGATAATATT

TTGGACTCGCGCAGAGCTTTACTTGAGTAGCAGTTTTTATGATCATTAGTTGTACCTACT

GCATGCATTTCACTTTTCTCCACTCTAAGTTTTGAACAGTTAAAAAAAAAAAAAAAAAAA

AAAAGCGGCC

>OscRunx predicted peptide translation MRLMMEREPAPKRSKDSLELSSSMGTLSASASAAAEHQGDLVKTDNPNFVCTILPSHWRV

NKTLPVPFRVLAVGDISVPDGVKVTLKAFNEETVSGELRNATAIFRNNVARFNDLRFVGR

SGRGKYFDVLITVQTDTVQKAIYKKAIKVTVDGPREPRRHKVKERQLLAAHQHHHSPYHG

YPNRQHVLPPDFMPLSSAAASSLSSSSSSLGSAGCETPQLQRAIHRDSLSAFSTIATEPI

MRRPAFSQPMVTMNHVHYTQEPTTMSTQMEQSHCPSIPPSISMPPTFTSEASLSMLAGPA

FAPRQSVPGHIEQGFVFPPPFPLRSPTSAAGFAFPGGPPGLVPLSPIPGDSASPFPHRNY

RPIACQVSISSHGRSSGSGYDAPAPAMPGLVSTSDLFSLPVTPRTPITPTVRYAQMHAAQ

AAGQLMATSSSDALGYGHLTAAIGSFPFDQYATHLQDIQHMQQHSASMTSLNGEPSSSGV

TITLSPPPNKAKPTLSRSSSFTSGAGNGKMDSGETDEKEGLWRPY
