# Supplementary material for: The evolution of Runx genes II. The C-terminal Groucho recruitment motif is present in both eumetazoans and homoscleromorphs but absent in a haplosclerid demosponge
Source: BMC Res Notes. 2009 Apr 17;2:59. doi: 10.1186/1756-0500-2-59 (PMC2674455; doi:10.1186/1756-0500-2-59)
Supplement: Additional File 3 — AmqGroucho sequence. This file provides an A. queenslandica genomic trace sequence that encodes peptides homologous to Groucho, identified by tBLASTn using the TLE-domain (pfam03920: TLE_N), and confirmed by reciprocal BLASTx. [file 1756-0500-2-59-S3.doc]

>BAYB653482.b1 (AmqGroucho fragment from genomic trace, containing two exons encoding the part of the N-terminal Q-rich oligomerization domain (pfam03920: TLE_N)

NNNGGTTTTGCANNNNACCAGATCTCTGTATGTGTTTTCTAGAAGTATCTCTTATAGTTC

ATATTGTACACACACACACACACACTCACTTACTCTGTTAAGTTTAGAAATGTGTCTTTT

GATTTCTAAACAAAGTATGCTCCCAGTGATAACAAGAGGCTATAAAACAAACAAGATTAG

TTTCAGATTCTCTCACTTTGTCAACACTGGAGTTAAATTGTACTAAAACCATTGCCCCTG

AGGAAATCTGACTTTTAGCAAAATGGCAACAATTTTTAATAATACATGCTCATGTTCCTT

TTGTCTCAATTTTCAGTTTAAAGCGAGAATTGGAAAAGATATCTAACGAGAAGACGGATA

TACATCGTCACTACATAATGGTGATAACCACTGTTAGATCTAGAGGGAGTTTAGGAGACT

TTTGGTCTCTCATATTTGGTCTAGGTCTCCTAAAATCTTCCATTTTTAGCTTTTTTAGTT

AGGTTGGTTTCCCAAATTTATTTATAAACCTAACAGTATGCATGTACATGTATGTGATAT

ACATGTACATGTATATTATGCATGTGAATATAGTGATTGTATTTGATACAATGTTAATGT

CTGTATGTTCTCTCTTACTGTAGTATTATGAAATGTCGTATGGACTGAATGTTGAGATGC

ACAAACAAACTGAAATAGCAAAGAGATTGAATGCAATATGTGCCCAAGTGATACCATTCC

TTTCTCAAGAAGTATGTCAACATAATAATAATATAATTTGTTAGTTTTTATTGCTATATT

ACTATTGTTGTAAATGTAAATATACATGTATATGTACACGTACATGTACATGCATTTTCT

TAATAATTAATAATTTTATGTTCATATGATCCTGTTTGTACTT
