# Supplementary material for: The evolution of Runx genes II. The C-terminal Groucho recruitment motif is present in both eumetazoans and homoscleromorphs but absent in a haplosclerid demosponge
Source: BMC Res Notes. 2009 Apr 17;2:59. doi: 10.1186/1756-0500-2-59 (PMC2674455; doi:10.1186/1756-0500-2-59)
Supplement: Additional File 4 — AmqRunx cDNA sequence. This file provides the cDNA sequence of AmqRunx containing the N-terminus, Runt domain, predicted C-terminus, and some 3' UTR, obtained by RT-PCR from adult and embryonic RNA. [file 1756-0500-2-59-S4.doc]

**Additional File 3**

>Amqrunx cDNA (with cds + 3' UTR)

ATGTCTTCTGAATCAATCCCACCTCCTGATGGTCCCCTACCCCCTCCCTCCTCCAAACGCTATCGAGGAGAAAGAACCTTCTCCGAACTATTAGCGGAATACCCTGGAGAACTCGTAACGACCGATAGTCCTAACTTTGTATGTACTATCCTTCCGTCCCACTGGAGATGTAATAAGACCTTGCCCGTACCGTTTAAAGTACTATCGCTCTCCGATATCACTGATGGTACCAAGGTGATACTAACAGCTGGTAATGATGAGAACTCAGCCGCTGAGCTTAGGAACGCCATAGCAACTTTTAAGAACCAAGTGGCTCGGTTTAATGATCTTCGCTTTGTCGGTCGATCGGGAAGAGGTAAAATGCTGACAGTAACAATCACCATAGTAACAGAGCCGGTACAATACGCTACTTACTCTCACGCAATTAAAGTGACAGTGGATGGACCAAGAGAACCAAGACGTAACAGAGCCTCTACTAGATCAGATGATCATCCTTACCTAAGGCCTAATCCATTTATGGGTCATTTGTCACCAGCTGGAGCGGGTCAGGTCCCTCCATTAATTAAAGACACAAGGTGTCCTCCATCTGGCAGTATAGACCTTGAAGGTGCCATGGCAACCGATCCTTCTTGTAGACCACCTAGTATGTCTGATGTCTTCCCAGCTGCTTTTCAAACTAACGCTGACGTACCCCTTCCTGCCTGGCCTCCATCATCTCCTCACATACCAGGTATAAGGTCTCCGGTGTGGCAGTATCCTGGTGTCATTACCAGTCAGTCCCTCATTCCCTCTCAATTGGATACCTCAAGTACCAACTCAGTCCCCCAAACCAGTGCCGATAGTTTAAGCAATGGTAGCTCGACTCCGCCCAACGTCACTCAGAACGGTGACCATGCCCAGATTAATAACAGCACCGGGAATGTTAACGACTCCAAATTCCTATTCCCGTCAGGCAGTGCCATCCCTCTTTCCCCTGGACTGTTCAATGCTCAGTCGTTCTTTAATCCTGGAGGAAGTAACAATATCCCTATAACACCAACGCTACTAGCTCCTTCTGTCTCCTTCTCAGAATCCTACATTAGACCAGGTCAAATCTACTCTCCTTTCTCGTTCACGCCCCATGGCAGTCTCCATGGCAACCTCCCCCGTACCCCCACCCTCCCCCCTCCTTCCCCTCATGCCATTGTTAGTTGCTCTTCTTTCCCTGCTCTAACGGCCATTGCCCACCCCTCCTTCTCCACTAGTAGTCTTCAATTTCAAAAAGGTGGTTCCTTCCTTGACGACATTACTAAGATTGGCTCCATATCTCCATTCATAGTCTCTCCATCTCTCTCTCCTAATCGACGTCCAAATGGCACCACAATATTCTTTCCGACGACCATCACAGCTCAAGGTGAAGCTAAGTTCGCTACCATAGGAGAGGTGGGTATGGCCGGGTCTGTGGGCGGAGTCGAGAGGTTATCTGGTACGCCAGACGATGGTAGCATCCATTCTACCGATTCACCGTTGATCAAGAGAGAAGTGTCGTCACCACAACATTGTTACATAGACCAGGAGGCAGCCTAAGGAGGAGGAGAAATAATAATAATAATAATAATAATGACGATAATTTAATGATTATAAAATTACAATTCTTTCTCTTCATTCATTTCATATTCTGACTGGCACCACAACCCCACACAATCACTCTCCATCCTACCACCACTCACAGGATAATTTCATGTCTCTGCTTCCCCTCTCTCCCTCCCTCCCTCCCTCCCTCCCTCTCTCTTATAAAGTCACTTGTTATCATTATTATTATTATTATTATTATTATTATTGTTGTTGTTGTTATTTTTTCATTCAAACTTTCTTTCTACAATCAGTGACATTCTTTTTTGTATAGTTTATTTTATTTTTGTATCTTCAATCTAGTAAATTTTGACAATCTCTCTCTCTCTCCCTCTTTCTCTCTTTTGTAATATATTAATAATTATTATTCTGATGTCATGACACCAATCTCTTTCTGTCTCTTGTGTTTGTTTATTATCATTATTATTATTAATATTATTATTATTTCAAACTTGATGAGTTTTCCATCTCTTTTCTCTCGTTTATAATATTATTATTTTCCAATCTGCTCAAAATAATGAAATTAAACTGGCCAAACTTCCAAGCCACACTCCTCTCTCTTTCTCGTTCAAAACTGTTTT
